# Non-prescription antibiotic use and its predictors among children in low- and middle-income countries: a systematic review and meta-analysis

**DOI:** 10.1186/s13052-024-01808-5

**Published:** 2024-12-18

**Authors:** Segenet Zewdie, Assefa Andargie Kassa, Ashagrachew Tewabe Yayehrad, Mekonnen Melkie Bizuneh, Wondim Ayenew, Melkamu Zewudie, Selomie Mulat, Bayih Endalew Bitew, Serkalem Zewudie, Birhanu Geta Meharie, Tegenu Chanie Tesfaye, Aregash Abebayehu Zerga, Fanos Yeshanew Ayele, Husein Nurahmed Toleha, Birhanu Demeke Workineh, Ewunetie Mekashaw Bayked

**Affiliations:** 1 Department of Pharmacy, College of medicine and health science, Injibara University, Injibara, Ethiopia; 2 Department of public health, College of medicine and health science, Injibara University, Injibara, Ethiopia; 3 Department of pharmacy, College of medicine and health science, Bahir Dar University, Bahir Dar, Ethiopia; 4 Department of Reproductive Health, College of medicine and health science, Bahir Dar University, Bahir Dar, Ethiopia; 5 Department of social and administrative pharmacy, College of medicine and health science, University of Gondar, Gondar, Ethiopia; 6 Department of pediatrics, Tibebe Ghion Specialized Hospital, Bahir Dar, Ethiopia; 7 Department of Medicine, Saint Paul Specialized Hospital, Addis Ababa, Ethiopia; 8 Department of Pharmacy, College of medicine and health science, Debre Tabor University, Debre Tabor, Ethiopia; 9 Department of Medicine, Felege Hiwot Regional Referral Hospital, Bahir Dar, Ethiopia; 10 Department of Pharmacy, College of medicine and health science, Debre Markos University, Debre Markos, Ethiopia; 11 Department of Nutrition, College of medicine and health science, Wollo University, Dessie, Ethiopia; 12 Department of pharmacy, College of medicine and health science, Wollo University, Dessie, Ethiopia

**Keywords:** Prevalence, Non-prescription, Antibiotics, Children, LMIC

## Abstract

Globally antibiotics are among the most commonly used drugs. Non-prescription use of antibiotics is a major factor for the emergence and spread of antimicrobial resistance one of the top global public health and development threats. This systematic review and meta-analysis aim to assess non-prescription antibiotic use and predictors among children in Low and middle-income countries.

A comprehensive search of electronic databases was conducted from PubMed, Scopus and HINARI to identify primary studies published between 2000 and 2024. Observational studies conducted among children ≤ 18 years old and published in English language were included in the review. After screening, the studies were assessed using Joanna Briggs Institute (JBI) critical appraisal tool and data were extracted using a checklist. Heterogeneity was assessed using forest plot, Chocran’s Q Test and I^2^. The random effects meta-analysis model was employed to pool the prevalence of non-prescription antibiotic use among children in low-and middle-income countries. Sub-group analysis and meta-regression were performed to identify the sources of heterogeneity. Publication bias was assessed using funnel plots with Egger’s test.

The review was conducted among 32 cross-sectional studies with a sample size of 80,133 participants. The pooled prevalence of non-prescription antibiotic use among children in low-and middle-income countries was 38.86% (95% CI 34.32, 43.40; *P* < 0.0001) with high heterogeneity (*I*^2^ = 99.38%, *p <* 0.001). The prevalence of non-prescribed antibiotic use among studies conducted in upper middle-income countries (30.85% (24.49%, 37.21%)) was low when compared to studies conducted in LMICs (44.00% (37.72%, 52.09%). Penicillin was the most often antibiotic class used without prescription, while upper respiratory infections were the most prevalent illness/symptoms that prompted non-prescription antibiotic use.

The pooled prevalence of non-prescription antibiotic use among children in low-and middle-income countries is high indicating that two out of five children used non-prescribed antibiotics. This review is important for international organizations, ministry of health of the low-and middle- income countries, regulatory bodies and researchers.

**Supplementary Information:**

The online version contains supplementary material available at 10.1186/s13052-024-01808-5.

## Introduction

Antibiotics are among the most widely used medications in the world. Antibiotics have been a lifesaver since the 20th century, especially in low-and middle-income (LMIC) countries where infectious disease are the main cause of morbidity and mortality among children [1]. In LMIC, antibiotic consumption increased by 114%, from 11.4 to 24.5 billion defined daily doses between 2000 and 2015 years [2].The non-prescription use of antibiotics appears to be endangering this therapeutic value of antibiotics [3].

The World Health Organization (WHO) alerts that around 80% of antibiotics are utilized in LMICs for community-based medical care [2]. Additionally, it has been documented more than two-thirds of antibiotics available in the pharmaceutical sector in LMICs are used without prescription [4]. Throughout the world, community medicine retail outlets (CMROs) are the main sources of antimicrobials [5]. According to the recent multi-country public awareness survey conducted by WHO, 93% of people got their most recently taken antimicrobial from a pharmacy and drug store [6].

Non-prescription antibiotic use in children is influenced by a number of factors, such as the mildness of the illness, accessibility, price, and healthcare-seeking habits [7]. The most common infections in the community including viral origin respiratory tract infections are the causes of non-prescription antibiotic use among children [8, 9]. Children are primarily affected by self-limiting diseases and infections of diverse etiology. Antibiotics are therefore known to be given to children more frequently than any other type of medication [10, 11].

Non-prescription use of antibiotics is defined as intermittent or continuous use of antibacterial agents to treat self-diagnosed diseases or symptoms without medical guidance. Despite being prescription-only drugs (POMs), antibiotics are widely used without a prescription all over the world.

Globally non-prescription use of antibiotics among children ranges from 1% [12] to 93.5% [13]. According to a review conducted, non-prescription use of antibiotics among adults in LMIC ranges from 50% to 93,8% with a pooled prevalence of 78% [14]. Particularly in LMICs, non-prescription antibiotic use is worsened by a number of factors including weak regulatory frameworks, inadequate healthcare infrastructure, limited access to high-quality medical services, limited diagnostic capabilities, and low levels of awareness and education about appropriate use of antibiotics [15–18].

Non-prescription use of antibiotics poses a great risk to the global public health in general and to the person taking it in particular [19]. It is the major factor for the emergence and spread of AMR (antimicrobial resistance) which is one of the top global public health and development threats.

AMR affects countries in all regions and at all income levels even though, its drivers and consequences are exacerbated by poverty and inequality, and LMIC are most affected. AMR has detrimental effects on both health and the economy. AMR has led to adverse consequences, including severe illnesses, more prolonged hospital admissions, increased healthcare costs, an overburdened public health system, higher costs in second-line-drugs, treatment failures, and even increased mortality rates [20–25].

Globally around 700,000 deaths per year have been triggered due to antibiotic resistance of which around 200,000 are newborns. It is estimated that bacterial AMR was directly responsible for 1.27 million global deaths in 2019 and contributed to 4.95 million deaths [26]. Antibacterial resistance may have detrimental effects that result in up to 10 million deaths by 2050, and associated expenses could reach up to USD100 trillion worldwide [27]. AMR has significant economic costs. The World Bank estimates that AMR might result in USD 1 trillion to USD 3.4 trillion gross domestic product (GDP) losses per year in 2030 and USD 1 trillion additional healthcare costs in 2050 [28, 29].

Reducing non-prescription use of antibiotics among children is one of the key issues of the general public in the fight against antimicrobial resistance [30]. The WHO and the United Nations (UN) General Assembly approved the introduction of AMS (antimicrobial stewardship) programs internationally and at the institutional level in 2016 in an effort to combat AMR caused by inappropriate antibiotic use. Though AMS programs have proven to improve antibiotic use in developed countries, AMS strategies are unsuccessfully executed in LMIC. As a result, there is a pressing need to create, carry out, and assess successful AMS programs in these regions. WHO recently released an AMS toolkit for developing countries, emphasizing the significance of local context in the design and execution of AMS initiatives [31].

Despite AMR due to inappropriate antibiotic use is rapidly growing at alarming rate in LMICs along with high morbidity and mortality [32–36], it continues to receive a relatively low public health priority. Even though, many countries developed national AMR action plans, the implementation is still inadequate [37–39]. Although there are Antimicrobial Stewardship Program committees in hospitals, it is not functioning according to standard requirements [40]. This may be because of limited resources and lack of awareness among prescribers, policy makers, the general public and international private or public health agencies regarding the prevalence and economic as well as clinical impact of AMR [41]. While a single systematic review and meta-analysis of children’s non-prescription antibiotic use in LMICs has been done, the majority of the included studies used simulated patients. As a result, it depicts the practice of community pharmacies providing antibiotics without a prescription rather than the actual non-prescription use of antibiotics among children. In addition, ten primary studies included in the current review were carried out after the publication of the previous review. Therefore, there is no previous SRMA that actually shows non-prescription use of antibiotics among children in LMICs.

Determining the pooled prevalence of non-prescription use of antibiotics among children can play a crucial role in figuring out the magnitude and to develop different interventional strategies useful in tackling its consequence. AMR which is attributed to non-prescribed use of antibiotics is a growing public health problem that adversely affects the lives of millions of individuals around the world. Therefore, it provides evidence to policy makers in developing strategies and regulations to prevent non-prescription antibiotic use in children. In addition, this study will contribute its part in improving the quality of health care in children. Moreover, it will specifically provide the necessary information for regulatory bodies and for appropriate intervention, monitoring and evaluation to prevent non-prescription use and dispensing of antibiotics and decrease the development of AMR. Lastly, the findings from this study can be used as evidence for researchers in the urge to conduct further investigations. Therefore, this review aimed to estimate the pooled prevalence of non-prescription use of antibiotics and its associated factors among children in LMICs.

## Methods

### Study design and search strategy

A systematic review and meta-analysis of published studies were used to determine the pooled prevalence of non-prescription use of antibiotics among children in low-and middle-income countries. The Preferred Reporting Items for Systematic Reviews and Meta-Analyses (PRISMA) guideline was applied to report this review [42]. Primary studies were extensively searched from databases such as PubMed, Scopus and HINARI. In addition, we have used the advanced form of Google Scholar and citation tracking. The key terms used in searching studies were non-prescription, without prescription, over the counter, self-medication, self-prescription, antibiotics, anti-infectives, child, children, young, pediatrics, under five by a combination of Boolean operators “AND” or “OR” as applicable and the search was made by two authors independently (SZ and AA). The search was restricted to only ‘human studies’ and ‘published in English language’. An additional file shows search strategy in detail (Supplementary Material 1).

### Eligibility criteria

The inclusion criteria were delimited using the CoCoPop components.

Condition: Non-prescription antibiotic use.

Context: LMIC.

Population: Children who are 18 years old or younger.

Studies: Observational primary studies including cross-sectional and cohort studies.

Publication status: Published.

Time period: Studies conducted from 2000 to 2024.

Language: Studies published in English language.

Repeated publications, preprints, studies with incomplete information, studies did not report the outcome of interest and studies conducted among children who have history of self-medication were excluded from the review.

### Primary outcome

Non-prescription use of antibiotics: defined as taking any type of antibacterial drugs without prescription of a physician.

### Study selection, quality appraisal, and data extraction

The article screening activity was done by SZ and WA. Articles searched from different sources were exported to EndNote V.20, and then duplicates were identified and dropped. The titles of the remaining articles were evaluated such that studies with irrelevant titles were rejected and the abstracts and full texts of the remaining studies were reviewed. Two independent reviewers (SZ and ATY) performed the quality assessment appraisal. The quality of each article was assessed using the standardized Joanna Briggs Institute (JBI) critical appraisal tool prepared for cross-sectional [43]. The tool has ‘Yes’, ‘No’, ‘Unclear’, or ‘Not applicable’ types of questions, and scores were given 1 for ‘Yes’ and 0 for ‘No’ and ‘Unclear’ responses, respectively. Scores were summed and transformed into percentages. Those studies that scored ≥ 50% were taken for both systematic review and meta-analysis of non-prescription use of antibiotics among children. When there were any scoring disagreements between the assessors, the sources of discrepancy were investigated by a thorough discussion. For persistent disagreements despite the detailed review, a third independent reviewer (BDW) was assigned as arbitrator.

We developed a data extraction sheet using a Microsoft Excel worksheet which was then, pre-tested on five randomly selected included studies and the checklist was modified accordingly. Information such as the name of the first author, publication year, study design and setting, the country the study was conducted, income level of countries, sample size, response rate, prevalence of non-prescription use of antibiotics, mean age, male/female ratio, recall period and major illness were included in the data extraction tool. One reviewer author (SZ) extracted the data from included studies and the last author (EMB) checked the extracted data.

### Statistical methods and analysis

The extracted data was exported to STATA/SE V.17 for further analysis. Forest plots were used to present the prevalence and predictors of non-prescription use of antibiotics among children. It provides a visual inspection of the confidence intervals of effect sizes of individual studies. The existence of heterogeneity among studies was assessed using the forest plot, the Cochrane Q statistics and the I^2^. The presence of non-overlapping intervals suggests heterogeneity. Significance of heterogeneity was declared using Q statistics at p-value *<* 0.1. Heterogeneity test (I^2^) of ≥ 50% and a p-value of < 0.05 was declared as the presence of heterogeneity [44]. The confidence intervals were computed using the exact method. The DerSimonian and Laird (D-L) method for the random effects model was applied in the meta-analysis of the prevalence of non-prescription use of antibiotics. A funnel plot was used to detect and examine publication and small study biases. The funnel plot asymmetry was statistically checked using Egger’s test [45]. Accordingly, asymmetry of the funnel plot and/or statistical significance of Egger’s regression test (p-value *<* 0.05) were suggestive of publication or small study bias.

Subgroup analysis was performed by using study year, income level of countries, region the study conducted, study setting and recall period as grouping variables and sources of variation. Meta-regression was also conducted for the prevalence of non-prescription use of antibiotics using sample size as covariate. To check the influence of a single study on the effect size, a sensitivity analysis was performed using the random effects model. Moreover, sensitivity analysis was performed by changing random effect model into fixed effect model and excluding studies with small sample size.

## Results

### Study selection

**Fig. 1:**
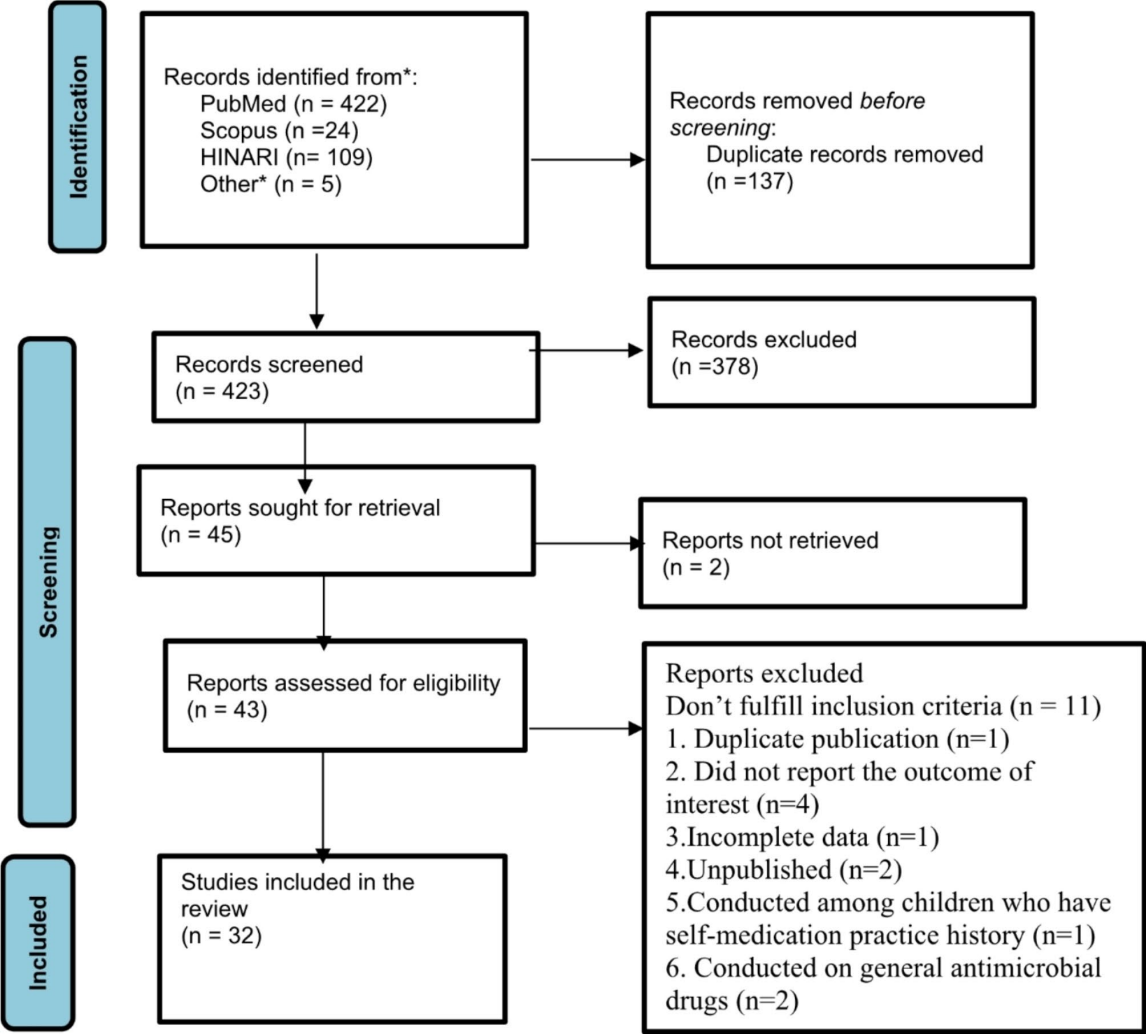
PRISMA flow diagram of included studies in the systematic review and meta-analysis of non-prescription antibiotic use and its predictor among children in LMIC 2000 to 2024

Electronic searches throughout all databases, search engines, and citation tracking turned up a total of 560 studies. After 137 duplicates were eliminated, 423 studies were selected for screening by looking at their abstract and title. Of those, 378 studies were eliminated since they were not related with the aim of the study. As a result, 45 studies were sought for retrieval. The full-text of 2 studies cannot be retrieved. Therefore, 43 full-text publications were evaluated for eligibility; of these, 11 did not meet the requirements for inclusion and were excluded in the study. Of the studies that were not included in the review, one was repeated publication [46] and four failed to report the desired outcome [47–50], two were conducted on general antimicrobial drugs [51, 52], two were not published [53, 54], one was letter to editor [55], one was conducted among children who are self-medicated any type of drug [56]. The remaining 32 met the inclusion criteria and were included in the review (Fig. 1).

### Study characteristics

A total of 32 [57–88] studies were included in the systematic review and meta-analysis. All the included studies were cross-sectional and 11 were institution based [58, 60, 67, 68, 71, 72, 75, 81, 82, 86, 88]. Eleven of the included studies conducted in China [57, 61, 66, 69, 73, 74, 76, 77, 79, 83, 84], 2 were conducted in Iran [64, 80], two were conducted in Peru [62, 71], two were conducted in Tanzania [72, 82], two were conducted in Uganda [63, 81] and the remaining 14 studies were each from Jordan [70], Iraq [75], Pakistan [85], Philippines [67], Yemen [58], Mongolia [59], Ecuador [65], Bangladesh [87], Morrocco [88], Egypt [86], Tunisia [78], Nigeria [60] and Camerron [68]. Twelve studies were conducted among children five years and younger [59, 60, 62, 63, 65, 72, 76, 77, 80, 81, 86] and three studies were conducted among children under 15 years old [58, 68, 85]. The sample size of the included studies ranged from 100 [86] to 39,224 [61] with a total sample size of 80,133 participants. Of the included studies eighteen [58, 60, 61, 63, 64, 66, 68–71, 73, 74, 76, 78, 79, 81, 85, 87] reported the sex ratio of the participants and more than half of them were females (35,969). In sixteen studies [59, 60, 62, 65, 67, 68, 72, 75, 76, 78–81, 84–86], the mean age of the respondents ranged from 30.0 [65] to 37.5 [86]. In nine of the included studies [57, 64, 69, 71, 72, 80, 83, 84, 88], the recall period was 1 year whereas 5 studies used 1 month [63, 73, 74, 77, 81] and the recall period of 4 included studies were 6 months [59, 66, 76, 86]. Majority [24] of the included studies [57–59, 62, 66–72, 76–80, 82–85, 87–89] assessed non-prescription antibiotic use for any type of illness (Table 1).

**Table 1:** Characteristics of studies included in the systematic review and meta-analysis of the prevalence and predictors of non-prescription antibiotic use among children in LMIC from 2000 to 2024

Author, pub year	Country	Income level	Study design	Child Age (year)	Sample size	Sex (male/ female)	#Np AB use	Np AB use (%)	Recall period	Major illness
P. Bi et al., 2000	China	UMIC	C.CS	2 to 18	1459	N/R	521	35.7	12 months	Any illness
Mohanna, 2010	Yemen	LIC	I.CS	≤ 15	2000	1110/ 890	1200	60.0	15 days	Any illness
Togoobaatar et al., 2010	Mongolia	LMIC	C.CS	< 5	503	N/R	212	42.3	6 months	Any illness
Ekwochi et al., 2013	Nigeria	LMIC	I.CS	< 5	210	124/ 86	98	46.7	N/R	Diarrhea
Ecker et al., 2015	Peru	UMIC	C.CS	≤ 5	1200	N/R	165	13.8	N/R	Any illness
Li et al., 2016	China	UMIC	C.CS	≤ 6	39,224	20,796/ 18,428	13,768	35.1	N/R	Diarrhea
Kibuule et al., 2016	Uganda	LIC	C.CS	< 5	199	92/ 107	86	43.0	1 month	URTIs
Zeinali, et al., 2016	Iran	LMIC	C.CS	7 to 12	372	201/ 171	197	53.0	12 months	Seasonal Cold
Quizhpe A et al., 2017	Ecuador	UMIC	C.CS	< 5	947	N/R	304	32.1	N/R	URTIs
Chang et al., 2018	China	UMIC	C.CS	< 7	3358	1119/ 2239	1617	48.2	6 months	Any illness
Bulario et al., 2018	Philippines	LMIC	I.CS	< 18	390	N/R	164	42.1	N/R	Any illness
Elong Ekambi et al., 2019	Cameroon	LMIC	I.CS	< 15	402	209/ 193	158	39.3	During data collect	Any illness
Sun C et al., 2019	China	UMIC	C.CS	< 13	9526	2243/ 7283	1927	20.2	12 months	Any illness
Tareq et al., 2019	Jordan	LMIC	C.CS	1 to 12	846	134/ 712	332	39.2	N/R	Any illness
Paredes et al., 2019	Peru	UMIC	I.CS	< 3	224	187/37	53	23.5	12 months	Any illness
Simon and Kazaura, 2020	Tanzania	LMIC	C.CS	< 5	730	N/R	292	40.0	12 months	Any illness
Xu et al., 2020	China	UMIC	C.CS	< 13	1275	N/R	410	32.2	1 month	Any illness
L. Lin et al., 2020	China	UMIC	C.CS	≤ 13	3188	1623/ 1565	594	18.6	1 month	URTIs
Shawq et al., 2020	Iraq	UMIC	I.CS	< 18	225	N/R	124	55.1	N/R	Any illness
J. Wu et al., 2021	China	UMIC	C.CS	< 5	1188	364/ 824	172	14.5	6 months	Any illness
Zhu Y et al., 2021	China	UMIC	C.CS	< 5	487		91	18.7	1 month	Cough
Mabrouk et al., 2021	Tunisia	LMIC	I.CS	< 18	354	36/ 318	73	20.6	N/R	Any illness
Wang, N.C et al., 2022	China	UMIC	C.CS	3 to 10	3056	986/ 1959	1161	38.0	N/R	Any illness
Nazari et al., 2022	Iran	LMIC	C.CS	< 6	1483	N/R	914	61.6	12 months	Any illness
Nyeko et al., 2022	Uganda	LIC	I.CS	6 m to 5	210	118 /92	83	39.5	1 month	Febrile illness
Mutagonda et al., 2022	Tanzania	LMIC	I.CS	< 5	2775	N/R	916	33.0	N/R	Any illness
Qu et al., 2023	China	UMIC	C.CS	7 to 14	1699	N/R	396	23.3	12 months	Any illness
Pei, D et al., 2023	China	UMIC	C.CS	6 to 12	961	N/R	568	66.5	12 months	Any illness
A. Salam et al., 2023	Pakistan	LMIC	C.CS	1 to 14	376	147/ 229	164	43.6	N/R	Any illness
S.H. Hafez et al., 2024	Egypt	LMIC	I.CS	< 5	100	N/R	41	41.0	6 months	Any illness
Islam et al., 2024	Bangladesh	LMIC	C.CS	< 18	704	153/ 551	408	58.0	N/R	Any illness
Elhaddadi et al., 2024	Morrocco	LMIC	I.CS	< 16	460	175/ 285	313	68.0	12months	Any illness

AB: Antibiotic, C.CS: Community based cross-sectional, I.CS: Institution based cross-sectional, LMIC: Low-and middle-income country, N/R: Not reported, Np: Non-prescription

Table 1. Characteristics of studies included in the systematic review and meta-analysis of non-prescription antibiotic use and its predictors among children in LMIC from 2000 to 2024.

### Risk of bias within studies

SZ and AA independently assessed the quality of individual studies using the JBI checklist. The check list has different type of questions, such as appropriateness of the sampling technique, adequacy of the sample size, validity of the measurement tool, adequacy of the response rate, appropriateness of method of analysis, and identification and handling strategies of confounding factors. The sample size of some of included studies was not adequate [63, 68, 71, 75, 81, 86] In some studies, the authors did not address the issue of non-responders [64, 66, 80, 85] and the selection of the participants is not representative of the source populations because convenience sampling was used [58, 65, 67, 84, 85]. Some of the studies included in the review did not describe the participants in detail [60, 70, 75, 78, 79, 84]. There was no persistent disagreement in appraising the studies. All included studies fulfilled the 50% quality assessment score for the review. An additional Excel file shows this in detail (Supplementary Material 2).

### Prevalence of non-prescription antibiotic use

**Fig. 2:**
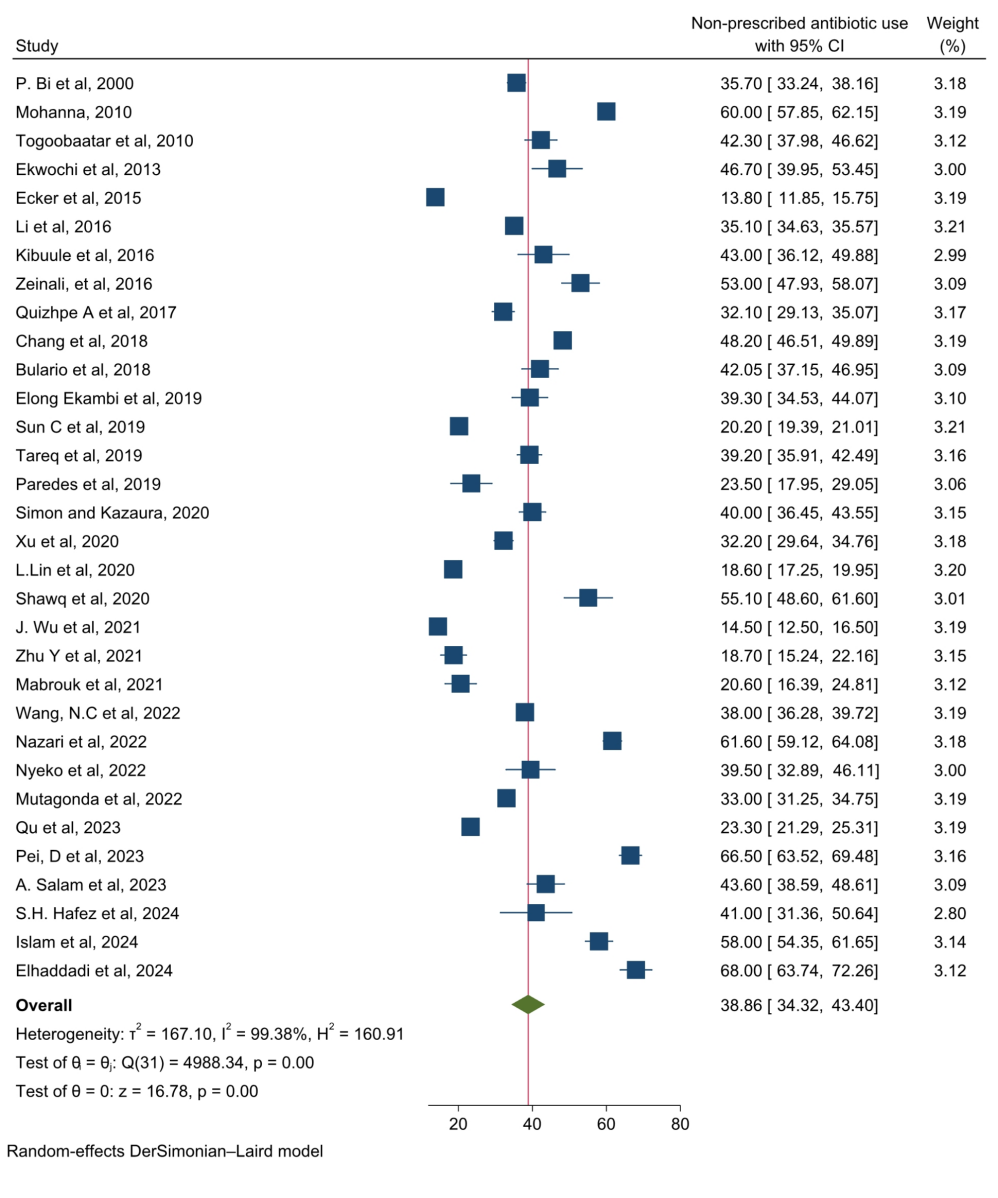
Forest plot of non-prescription antibiotic use and its predictor among children in LMIC, 2000 to 2024

A total of 27,522 children in LMIC used non-prescribed antibiotics. The lowest prevalence of non-prescription use of antibiotics among children were 13.6% [62] reported from Peru and the highest were 68% [88] reported from Morrocco. The pooled prevalence of non-prescription use of antibiotics among children LMIC were 38.86% ( 95% CI 34.32, 43.40; *P* < 0.0001). There was high heterogeneity between studies as evidenced by a significant heterogeneity chi-squared statistic (*Q* = 4988.34 (d.f. = 31), *p* value *<* 0.001) and *I*2 = 99.38% with *p* value *<* 0.001 (Fig. 2).

### Publication bias

The presence of publication bias was assessed using a funnel plot and Egger’s statistical test at a.

**Fig. 3:**
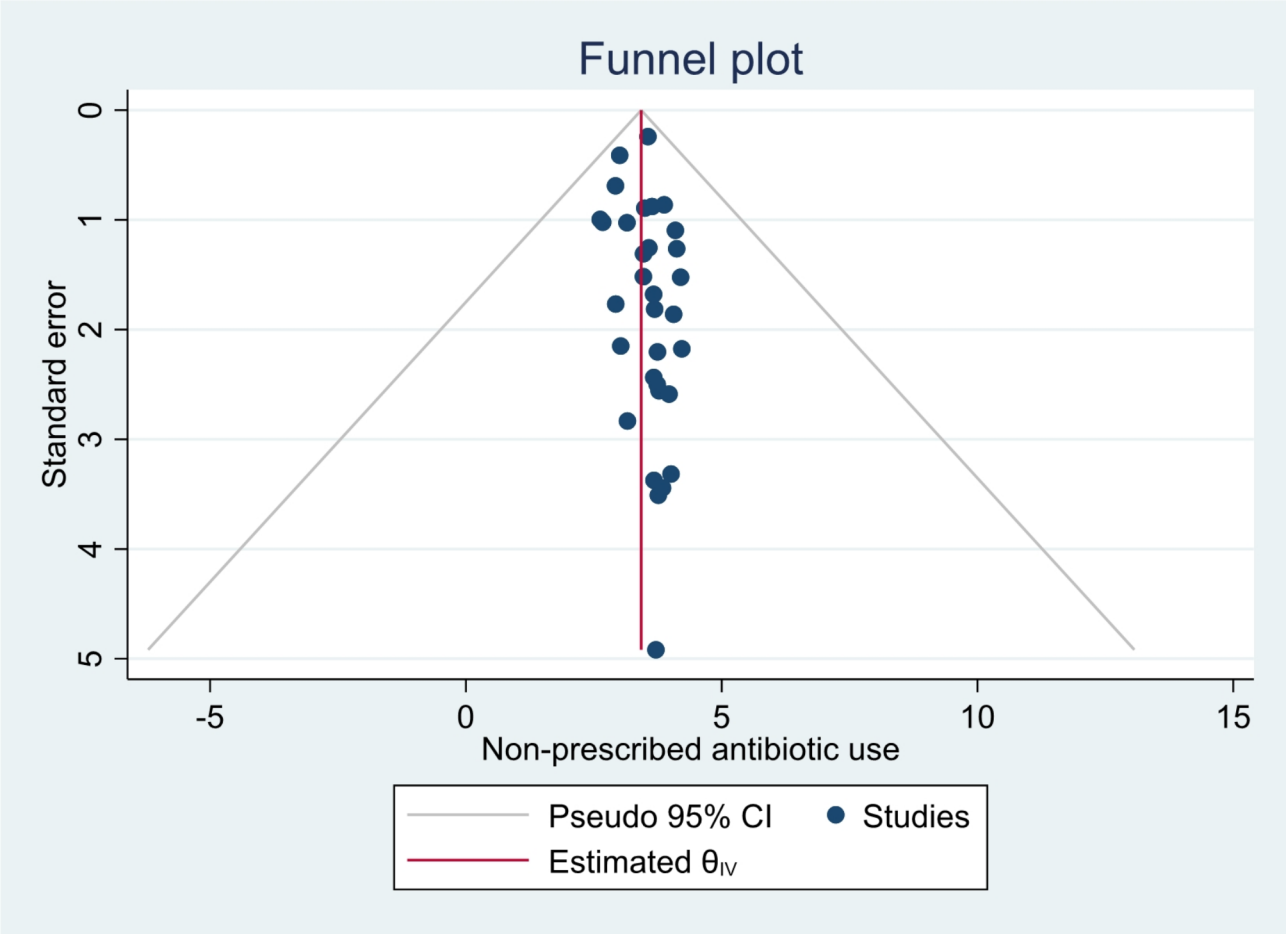
Funnel plot with 95% confidence interval of non-prescription antibiotic use and its predictors among children in LMIC, 2000 to 2024

5% level of significance. The funnel plot was performed by labeling the prevalence of non-prescription use of antibiotics (the effect size) on the x-axis and the standard error of prevalence of non-prescription use of antibiotics on the y-axis. The funnel plot results were asymmetric, indicating the presence of publication bias among the studies included (Fig. 3). But there was no significant publication or small study effect as evidenced by insignificant Egger’s test (*p* = 0.0876).

### Sensitivity analysis

**Fig. 4:**
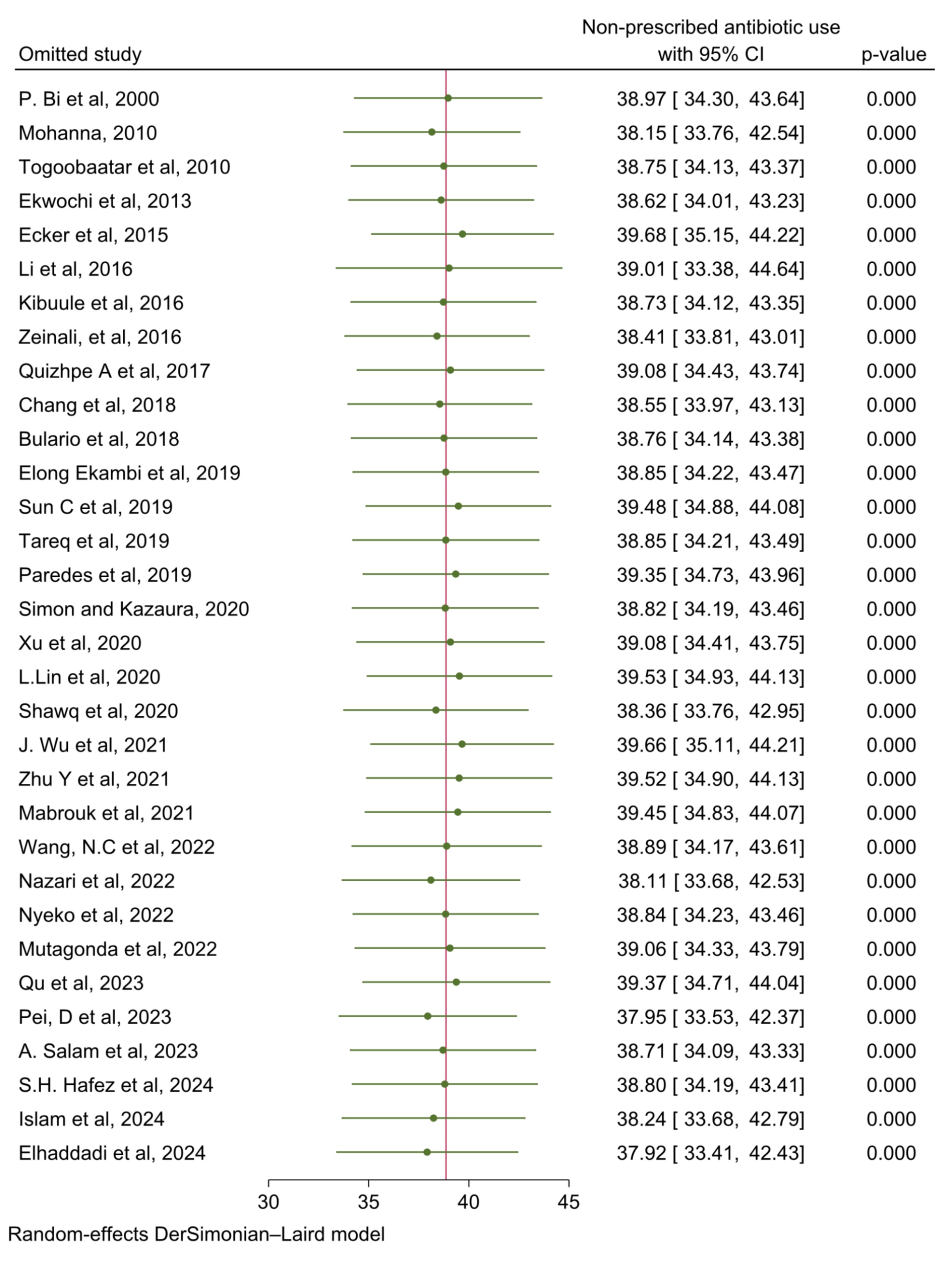
Sensitivity analysis of non-prescription antibiotic use and its predictors among children in LMIC, 2000 to 2024

To find the potential source of heterogeneity seen in the pooled prevalence of non-prescription use of antibiotics, we conducted a leave-one-out sensitivity analysis. The result of the sensitivity analysis found that the finding did not rely on a particular study. Furthermore, sensitivity analysis was performed by using fixed effect model and excluding studies with small sample size but there was no significant difference in the prevalence of non-prescription use of antibiotics (Fig. 4).

### Sub-group analysis

To detect the source of heterogeneity, subgroup analyses were done by study year, income level of countries, region the study was conducted, study setting and recall period for non-prescribed use of antibiotics among children. According to the subgroup analysis, the high heterogeneity was explained by income category of countries studies were conducted. The prevalence of non-prescribed antibiotic use among studies conducted in upper middle-income countries (30.85% (24.49%, 37.21%)) was low when compared to studies conducted in LMICs (44.00% (37.72%, 52.09%). (Table 2)

**Table 2:** Sub-group analysis of non-prescription antibiotic use and its predictors among children in LMIC, 2000 to 2024

Subgroup	No. of studies	Total sample size	Prevalence (95% CI)	Heterogeneity	
				I^2^(%)	*P* value
Study Year					
Before 2020	15	60,860	38.20 (32.01, 44.39)	99.44	< 0.001
2020 and above	17	19,271	39.48 (31.44, 47.52)	99.36	< 0.001
Income level					
UMIC	15	68,017	31.60 (25.76, 37.44)	99.54	< 0.001
LMIC	14	9,705	44.90 (37.72, 52.09)	98.07	< 0.001
LIC	3	2,409	47.76 (2.93, 62.59)	96.02	< 0.001
Region					
Asia	20	72,230	40.20 (34.41, 45.99)	99.55	< 0.001
Africa	9	5,440	41.19 (32.49, 49.89)	97.3	< 0.001
South America	3	2,371	23.10 (10.06, 36.13)	98.97	< 0.001
Study setting					
Community	21	72,781	36.94 (31.60, 42.28)	99.49	< 0.001
Institution	11	7,350	42.63 (32.90, 52.36)	98.51	< 0.001
Recall period					
12 months	9	16,914	43.52 (30.17, 58.88)	99.64	< 0.001
6 months	4	5,149	38.43 (15.87, 57.20)	99.54	< 0.001
<=1 month	6	7,359	35.29 (18.92, 51.68)	99.54	< 0.001
Not specify	13	50,709	37.92 (33.01, 42.83)	98.39	< 0.001

### Meta-regression

Further we investigated the heterogeneity using different statistical techniques to identify the source of heterogeneity. A meta-regression was performed by specifying the Der Simonian–Laird method for estimating the between-study variance. Sample size and response rate were used as covariates in the Meta regression analysis and none of them were significant and did not explain the source of heterogeneity (Table 3).

**Table 3:** Meta-regression of heterogeneity test for non-prescription antibiotic use and its predictors among children in LMIC, 2000 to 2024

Variables	Coefficients (95%CI)	Standard error	*P* value
Sample size	-0.153421 (-0.8106057, 0.5037638)	0.3353045	0.647
Response rate	-0.0002273 (-0.0010677, 0.000613)	0.0004288	0.596

### Perceived illnesses/symptoms that led to non-prescribed antibiotic use

Sixteen studies [57–59, 61, 63, 66, 67, 69, 70, 72, 77, 78, 83, 85, 86, 88] reported common illness/symptoms for which non-prescribed antibiotics were used. Of these, one study was conducted among children with diarrhea. The most common perceived illness/symptoms that led to non-prescribed antibiotic use among children were URTI, gastrointestinal symptoms and fever (Table 4).

**Table 4:** Perceived illnesses/symptoms for which non-prescribed antibiotics were used among children

Author, pub year	No. participants used Np antibiotics	Major Illness	Frequency (%)
P. Bi et al., 2000	521	Common cold Diarrhea Skin disease	222 (42.5) 200 (38.1) 94 (18.1)
Mohanna, 2010	1200	Respiratory Gastrointestinal	960 (80) 156 (13)
Togoobaatar et al., 2010	212	Cough Fever Throat symptoms	178 (84) 140 (66) 127 (60)
Li et al. 2016	13,768	Diarrhea	13,768 (100)
Kibuule et al., 2016	86	Common colds Common colds with coughs Common cold, sinusitis, and cough Common cold, cough, and throat infection Common cold cough with sinusitis and pneumonia	36 (41.9) 40 (45.5) 53 (61) 43 (50) 86(100)
J. Chang et al., 2018	1617	Cough Fever Running nose Sore throat Bronchitis Nasal obstruction	1240 (76.7) 661 (40.9) 555 (34.3) 520 (32.2) 474 (29.3) 351 (21.7)
Sun C et al., 2019	1927	Cold, sore throat, fever	
Tareq et al., 2019	332	Fever Cough and common cold Dysphagia Ear pain Other	137 (41.2) 44 (13.3) 47 (14.2) 42 (12.7) 62 (18.6)
Simon and Kazaura, 2020	292	Cough Fever Cold Diarrhea Headache	235 (80.5) 153 (52.4) 124 (42.5) 33 (11.3) 20 (6.8)
Zhu Y et al., 2021	91	Cough	100
Mabrouk et al., 2021	73	Sore throat High grade fever Flu-like symptoms Cough Fever	44 (60.3) 25 (34.2) 17 (23.3) 16 (21.9) 11 (15.1)
Qu et al., 2023	396	Cough Cold Throat pain Fever Diarrhea Bronchitis Pneumonia	236 (59.6) 205 (51.8) 166 (41.9) 148 (37.4) 73(18.4) 64(16.2) 27(6.8)
A. Salam et al., 2023	164	Sore throat Seasonal fever Abdominal pain	32 (19.7) 262 (16)
S.H. Hafez et al., 2024	41	Fever Cough Vomiting and diarrhea Sore throat Runny nose	41 (100) 21 (53) 31 (76) 41 (100) 26 (64)
Bulario et al., 2018	164	Cough Wound Sore throat Common cold Diarrhea Fever Vomiting	55 (33.6) 47 (28.7) 44 (26.8) 10 (6.1) 5 (3.0) 3 (1.8) 2 (1.2)
Elhaddadi et al., 2024	313	Cough Fever Sore throat Otalgia Diarrhea Abdominal pain Headache	135 (43) 75 (24) 28 (9) 28 (9) 28 (9) 16 (5) 3 (41)

Np: Non-prescription

Table 4: Perceived illnesses/symptoms for which non-prescribed antibiotics were used among children.

Six studies [59, 66, 70, 75, 78, 86] reported reasons for which non-prescribed antibiotics were used among children. The most common reasons for using antibiotics without prescription were previous experience with similar symptoms and drug, perceived mildness of illness, time and cost saving and inaccessibility of health care (Table 5).

**Table 5:** Reasons of non-prescribed antibiotics use among children

Author, pub year	No. participants Np AB use	Reasons	Frequency (%)
Togoobaatar et al., 2010	216	Mild illness/symptoms Same antibiotic prescribed for similar symptoms previously	151 (70) 32 (15)
J. Chang et al., 2018	1617	Follow previous prescription’ Mild symptoms	1253 (77.5) 584 (36.1)
Tareq et al., 2019	332	Mild illness Previous experience with the drug Lack of time Lack of money Others	121 (36.4) 121 (36.4) 17 (5.1) 48 (14.4) 26 (7.7)
Shawq et al., 2020	124	Low financial state Mild symptoms/illness Not availability of health care services Previous experiences Same medication always prescribed	15 (12) 24 (19.1) 32 (26.2) 33 (26.7) 21 (16.7)
Mabrouk et al., 2021	73	Same antibiotic prescribed to similar symptoms Lack of time Financial problems Self-medication was only a temporary solution.	43 (58.9) 16 (21.9) 15 (20.5) 14 (19.2)
S.H. Hafez et al., 2024	41	Previous experience with the disease Lack of time Cost saving Lack of accessibility to the health care service	27 (65) 19 (45) 25 (62) 27 (67)

### Common antibiotics used without prescription

Thirteen studies [53, 58, 59, 63, 64, 67, 77, 78, 81, 83–85, 88] with a total sample size of 3843 participants (who used antibiotics) reported common antibiotics used among children without prescription. Penicillin was the most often antibiotic class used without prescription, followed by cephalosporines, for children. Of the total participants (3843) in sixteen included studies which used non-prescription antibiotics, 1219 (32%) individuals used WHO ‘Watch Group’ antibiotics (Table 6).

**Table 6:** Common antibiotics used without prescription among children

Author, pub year	No. participants Np AB use	Antibiotics/Group of antibiotics	Frequency (%)
Mohanna, 2010	1200	Amoxicillin Amoxicillin-clavulanic acid Trimethoprim-sulfamethoxazole Other	360(30) 240(20) 420(35) 180(15)
Togoobaatar et al., 2010	216	Amoxicillin Ampicillin Erythromycin Chloramphenicol Trimethoprim–sulfamethoxazole	125 (58) 54 (25) 13 (6) 11 (5) 11 (5)
Kibuule et al., 2016	86	Penicillin Sulfonamides Macrolides Aminoglycoside Amphenicol	37 43) 34 (40) 8 (9) 4 (5) 2(3)
Zeinali, et al., 2016	197	Amoxicillin Cephalexin Cefixime	80 (40.6) 75 (37.9) 24 (12.1)
Simon and Kazaura, 2020	292	Amoxicillin Cotrimoxazole Ampicillin/cloxacillin Cephalexin Erythromycin	181 (62.0) 36 (12.3) 30 (10.3) 27 (9.2) 23(7.9)
Zhu Y et al., 2021	91	Cephalosporins Penicillin Macrolides	48 (52.8) 28 (30.3) 15(16.8)
Mabrouk et al., 2021	73	Amoxicillin Amoxicillin and clavulanic acid Azithromycin Oxacillin Cefixime Pristinamcin	52(72.6) 9(12.3) 5 (6.9) 2 (2.7) 1 (1.4) 1 1.4)
Nyeko et al., 2022	83	Amoxicillin Erythromycin Ciprofloxacin Ampicillin	33(39.8) 18(21.7) 13(15.7) 6 (7.2)
Qu et al., 2023	396	Penicillin’s Cephalosporins Macrolides Quinolones Sulfonamides	338 (85.4) 296 (74.7) 215(54.3) 90(22.7) 39(9.8)
Pei, D et al., 2023	568	Amoxicillin Cephradine Azithromycin Cefalexin Erythromycin Norfloxacin Penicillin Streptomycin Levofloxacin Chloramphenicol	308 (54.2) 177 (31.2) 143 (25.3) 106 (18.7) 71 (12.5) 45 (7.9) 40 (7.1) 35 (6.1) 31 (5.4) 2 (0.3)
A. Salam et al., 2023	164	Amoxicillin Azithromycin Cephalexin	52 (32) 21(12.6) 16 (9.6)
Bulario et al., 2018	164	Amoxicillin Cephalexin Co-amoxiclav Erythromycin Co-trimoxazole Cloxacillin Cefuroxime Penicillin	82 (50.3) 14 (8.5) 10 (6.2) 8 (4.9) 7 (4.1) 6 (3.6) 3 (1.8) 3 (1.8)
Elhaddadi et al., 2024	313	Amoxicillin-clavunilic acid Amoxicillin Trimethoprim-sulfamethoxazole Azithromycin Other	150 (48) 75 (24) 46 (15) 26 (8) 16 (5)

AB: Antibiotic use, Np: Non-prescription

Table 6: Common antibiotics used without prescription among children.

### Sources of antibiotics

Eight studies [58, 59, 65–67, 70, 72, 81] reported the sources of antibiotics used without prescription. The major sources of antibiotics were community pharmacies/ drug stores followed by leftovers and previous prescription (Table 7).

**Table 7:** Sources of antibiotics used without prescription among children

Author, pub year	No. participants Np AB use	Source of Antibiotics	Frequency (%)
Mohanna, 2010	1200	Pharmacies and drug stores Previous prescription	888 (74) 312 (26)
Togoobaatar et al., 2010	216	Pharmacy	186 (86)
Quizhpe A et al., 2017	304	Pharmacy	267 (87.8)
J. Chang et al., 2018	1617	Pharmacy Left over	943 (58.3) 707 (43.7)
Tareq et al., 2019	332	Pharmacy Left over Others	289 (87.0) 33 (9.9) 999(3.1)
Simon and Kazaura, 2020	292	Drug stores	291(99.7)
Nyeko et al., 2022	83	Pharmacy or drug shops Issuance from clinic Leftover Neighbor Other	30 (36.1) 28 (33.7) 10(12.0) 6 (7.2) 9 (0.8)
Bulario et al., 2018	164	Pharmacies Health centers	140 (85.4) 38 (23.2)

### Predictors of non-prescription antibiotic use

In the current systematic review, the following factors were found to be predictors of non-prescription use of antibiotics among children in low and middle-income countries. Respondents female sex [76, 82, 88], parents young age [72], parents old age [67, 78], male child sex [53, 80, 81], older child age [57], distance to health facility [72], educational status of mother/caregiver [57, 63, 71, 82], rural / semi urban residence [70, 71, 76, 81], comorbidity [70], keeping antibiotics at home [59, 66, 69], easy access to antibiotics [74], long duration of symptom [77, 83], low annual income [72, 77], higher perceived barrier [76], parent’s ability to identify/name antibiotics [78] and having children’s health insurance [57] shows association with non-prescribed use of antibiotics. We cannot include many factors in the meta-analysis because studies used different classification of variables. Therefore, in the current meta-analysis we use only four factors including respondents/caregivers’ sex, child sex, residence and comorbidity but none of them did not show association with non-prescription antibiotic use among children (Table 8).

**Table 8:** The association of child sex, respondents’ sex, residence and comorbidity with non-prescription antibiotic use among children

Variable	Odds ratio(95%CI)
Child sex (Male)	1.16 (0.79, 1.71)
Respondents/ caregivers sex (Female)	0.82 (0.59, 1.14)
Residence (Rural)	0.19 (-0.24, 0.63)
Comorbidity	0.86 (0.38, 1.95)

## Discussions

The current systematic review and meta-analysis aimed at determining the pooled prevalence of non-prescribed antibiotic use among children in low- and middle-income countries. Non-prescribed antibiotic use is a major cause of irrational use of antibiotics, AMR, high hospitalization rate, and high economic as well as clinical burden to the individual and community at large. The pooled estimate of non-prescribed use of antibiotics among children in low- and middle-income countries was 38.86% ( 95% CI 34.32, 43.40; *P* < 0.0001). There are numerous explanations given for why children in low and middle income countries highly used antbiotics without prescription. Some of the factors attributed non-prescription use of antibiotics include weak regulatory system against dispensingof antibiotics without prescription, inaccessability of healthcare, high prevalence of childhood infection, poverity, lack of and ineffective health insurance and poor understanding towards the impact of non-prescription use of antibiotics [81, 90–98]. This implies that low and middle income countries should strengthen their regulatory system, prevent childhood infection, increase health care accessibility, improve health insurance coverage and awareness of the public towards the impact of non-prescription antibiotic use among children.

The result of the current review’s align with earlier systematic review and meta-analysis conducted on self-medication with antimicrobials in developing countries (38.8%) [8], a previous review conducted among university students in LMIC regarding antibiotic self-medication (46%) [89] and a review conducted in Ethiopia among adults (46.1%) [99].

However, the finding of our review is lower than the result of a previous review conducted in LMIC regarding self-medication practice towards antibiotics among adults (78%) [100]. The possible justification for this difference may be due to a previous systematic review and meta-analysis were included only 11 studies, conducted among adults with a total sample size of 5080. In addition, our finding was lower than the result of a previous systematic review and meta-analysis conducted in Eastern Mediterranean WHO region 49.7% [101]. The possible justification for this may be due to difference in the number of studies included, study period, socio-economic status of countries, accessibility of health care services and regulatory systems of countries.

However, our finding was higher than the result of a study conducted among under five children using DHS (demographic and health survey) data of 45 LMIC [102]. According to this study, only 16.9% under five children in 45 LMIC used non-prescribed antibiotics. The possible justification for this discrepancy may be due to a difference in the type of data used, the sample size, the denominator of the studies included in our review to that of a study used DHS.

The result of this systematic review and meta-analysis indicated that non-prescribed antibiotic use among children was 388 per 1000 varied from 136 per1000 to 680 per1000. Our finding was higher than a previous study conducted in nineteen European countries in which the prevalence of self-medication with antimicrobials varied from 1 to 210 per 1,000 [103]. Difference in accessibility of healthcare services, infection/disease prevalence, regulatory system, availability of antibiotics without prescription and knowledge towards the impact of non-prescription antibiotic use may be a possible justification for the discrepancy.

In our study the most frequently reported illness/symptoms for which antibiotics were used without prescription were URTIs followed by GIT problems and febrile illness. This finding is in line with the result of reviews conducted in Africa [104], developing countries [8], among the lay public in LMIC [105], Middle East [106], LMIC [100], Ethiopia [99] and a study conducted in Europe [103]. In all of this reviews URTI including cough and common cold were the most common reported illness/symptoms for which antibiotics were used without prescription among children despite bacteria being not the common cause of cough and common cold. This implies that health education targeted or emphasized at commonly reported indications including their common etiology and management should be given to the community of LMIC.

Penicillin was the most common class of antibiotics used without prescription among children in LMIC according to the review. This finding is consistent with previous studies and reviews conducted in Europe [103], developing countries [8], LMIC [100], Africa [104] and Ethiopia [99]. This may be because the most common indication was URTI and frequently reported reason was previous experience. Since penicillin including amoxicillin is the most common prescribed drug for URTI based on their previous experience with similar symptoms and drugs, individuals may prefer penicillin for non-prescription use. Moreover, in our study 1219 (32%) participants used WHO ‘Watch Group’ antibiotics. This implies that intervention strategies including health education emphasized at the impact of using antibiotics on individual child, parents, community, health care system and to the whole world should be implemented in LMIC.

Previous experience, perceived mildness of illness/symptoms, cost and time saving were the most frequently reported reasons for non-prescription use of antibiotics in LMIC which aligns with the findings of other reviews conducted in WHO Eastern Mediterranean region [101], Africa [104], and Ethiopia [99].

In the current review, the most common sources for antibiotics used without prescription among children in LMIC were community pharmacies. Similar finding was reported by previous reviews conducted in Europe [103], WHO Eastern Mediterranean region [101], Middle east [106], LMIC [100], developing countries [8], Africa [104] and Ethiopia [99]. This shows that community pharmacists are responsible for the extensive non-prescription antibiotic use among children in LMIC. Therefore, the laws and regulations LMIC should be strictly implemented in the community pharmacies. Because lax regulations or enforcement might allow pharmacies to dispense antibiotics without proper prescriptions, contributing to their frequent use as sources for self-medication.

However, the current systematic review and meta-analysis were not done without limitations. For instance, the current systematic review and meta-analysis estimated the pooled prevalence of non-prescription antibiotic use among children in the presence of high heterogeneity and including studies with different setting and recall period. Furthermore, only studies published in English language were included in this review, and some of the included studies used small sample sizes. Moreover, the result of the current review should be generalized cautiously because the included studies were only from nineteen countries which may not be representative to the whole LMIC. However, to the best of our knowledge, this is the first meta-analysis and systematic review on the prevalence and predictors of non-prescription use of antibiotics among children that tries to show the actual use in LMIC. As a result, this study has shed light on the issue and suggests more research to ascertain the causes of non-prescription antibiotic use as well as to develop interventional ways useful for prevention.

## Conclusion and recommendation

The pooled prevalence of non-prescription use of antibiotics among children in LMIC is high. The heterogeneity of this review is explained by income level of countries studies were conducted. The result of this study will be important to WHO, MOH of countries and other non-governmental organizations to develop different strategies that tackle against non-prescription use of antibiotics among children. Moreover, the finding is crucial for policy makers in giving aggregate data. Different interventional strategies should be adopted by WHO and health policy makers of countries in collaboration with other governmental and non-governmental organizations to prevent non-prescription antibiotic use through the implementation of educational initiatives and regulatory system promotion. Further research is required because this review determined the pooled prevalence of non-prescription antibiotic use using studies only from nineteen LMIC.

## Electronic supplementary material

Below is the link to the electronic supplementary material.

Supplementary Material 1

Supplementary Material 2

## Data Availability

All data generated or analyzed during this study are included within the paper and its supplementary information files.

## Declarations

### Registration

The review protocol was developed and registered in International Prospective Register of systematic review with registration number CRD42024563761 and available online at https://www.crd.york.ac.uk/prospero/#recordDetails.

## Abbreviations

AMR: Antimicrobial resistance
AMS: Antimicrobial stewardship
CI: Confidence interval
CMROs: Community medicine retail outlets
D-L: Der Simonian-Laird
DHS: Demographic health survey
GIT: Gastrointestinal tract
GDP: Gross domestic product
JBI: Joanna Briggs Institute
LIC: Low-income country
LMIC: Low- and middle-income country
MOH: Ministry of health
POMs: Prescription only medications
PRISMA: Preferred Reporting Items for Systematic Reviews and Meta-Analyses
SRMA: Systematic reviews and meta-analysis
UMIC: Upper middle-income country
URTI: Upper respiratory tract infection
USD: United states dollar
UN: United Nations
WHO: World health organization
