# Supplementary material for: Non-prescription antibiotic use and its predictors among children in low- and middle-income countries: a systematic review and meta-analysis
Source: Ital J Pediatr. 2024 Dec 18;50:260. doi: 10.1186/s13052-024-01808-5 (PMC11658204; doi:10.1186/s13052-024-01808-5)
Supplement: Supplementary file 1 — Supplementary Material 1 [file 13052_2024_1808_MOESM1_ESM.docx]

PuBmed(293)

((((Non-prescription) OR (Without prescription)) OR (over the counter)) AND ((Antibiotics) OR (Anti-infectives))) AND (((((((pediatrics) OR (Children)) OR (Child)) OR (under five)) OR (adolescent)) OR (School age)) OR (under 18))

1: Wang H, Wang H, Yu X, Zhou H, Li B, Chen G, Ye Z, Wang Y, Cui X, Zheng Y,

Zhao R, Yang H, Wang Z, Wang P, Yang C, Liu L. Impact of antimicrobial

stewardship managed by clinical pharmacists on antibiotic use and drug

resistance in a Chinese hospital, 2010-2016: a retrospective observational

study. BMJ Open. 2019 Aug 2;9(8):e026072. doi: 10.1136/bmjopen-2018-026072.

PMID: 31377693; PMCID: PMC6687004.

2: Carbonell-Duacastella C, Rubio-Valera M, Marqués-Ercilla S, Peñarrubia-María

MT, Gil-Girbau M, Garcia-Cardenas V, Pasarín MI, Parody-Rúa E, Aznar-Lou I.

Pediatric Medication Noninitiation in Spain. Pediatrics. 2022 Jan

1;149(1):e2020034371. doi: 10.1542/peds.2020-034371. PMID: 34957504; PMCID:

PMC9647521.

3: Kleinert E, Hillermann N, Jablonka A, Happle C, Müller F, Simmenroth A.

Prescription of antibiotics in the medical care of newly arrived refugees and

migrants. Pharmacoepidemiol Drug Saf. 2021 Aug;30(8):1074-1083. doi:

10.1002/pds.5254. Epub 2021 May 4. PMID: 33886141.

4: Vendrik KEW, Kuijper EJ, Dimmendaal M, Silvis W, Denie-Verhaegh E, de Boer A,

Postma B, Schoffelen AF, Ruijs WLM, Koene FMHPA, Petrignani M, Hooiveld M,

Witteveen S, Schouls LM, Notermans DW; MRSA consortium. An unusual outbreak in

the Netherlands: community-onset impetigo caused by a meticillin-resistant

<i>Staphylococcus aureus</i> with additional resistance to fusidic acid, June

2018 to January 2020. Euro Surveill. 2022 Dec;27(49):2200245. doi:

10.2807/1560-7917.ES.2022.27.49.2200245. PMID: 36695440; PMCID: PMC9732922.

5: Lee JH, Choi A, Noh Y, Oh IS, Jeon JY, Yoo HJ, Shin JY, Son SW. Real-world

treatment patterns for atopic dermatitis in South Korea. Sci Rep. 2022 Aug

10;12(1):13626. doi: 10.1038/s41598-022-17222-y. PMID: 35948589; PMCID:

PMC9365828.

6: Dutcher L, Li Y, Lee G, Grundmeier R, Hamilton KW, Gerber JS. COVID-19 and

Antibiotic Prescribing in Pediatric Primary Care. Pediatrics. 2022 Feb

1;149(2):e2021053079. doi: 10.1542/peds.2021-053079. PMID: 35102416; PMCID:

PMC9825803.

7: Pérez Solís D, Gómez de Oña C, Nicieza García ML, Suárez Gil P, Pérez Solís

P, Suárez Mier B, Rolle Sóñora V. Use of antibiotics in Paediatric Primary

Health Care before and during the COVID-19 pandemic. Enferm Infecc Microbiol

Clin (Engl Ed). 2023 Nov;41(9):529-534. doi: 10.1016/j.eimce.2022.12.004. Epub

2022 Dec 27. PMID: 36624035; PMCID: PMC9792423.

8: Vandenhaute J, Tsakeu E, Chevalier P, Pawaskar M, Benčina G, Vertriest J.

Assessing the use of antibiotics and the burden of varicella in Belgium using a

retrospective GP database analysis. BMC Infect Dis. 2021 Nov 11;21(1):1150. doi:

10.1186/s12879-021-06848-4. PMID: 34758734; PMCID: PMC8582146.

9: García-Moreno FJ, Escobar-Castellanos M, Marañón R, Rivas-García A, Manrique-

Rodríguez S, Mora-Capín A, Fernández-Llamazares CM. Adecuacy of pediatric

antimicrobial prescribing in the Emergency Department at discharge. An Pediatr

(Engl Ed). 2022 Mar;96(3):179-189. doi: 10.1016/j.anpede.2020.11.015. Epub 2022

Feb 28. PMID: 35241404.

10: Victoria M, Elena VB, Amparo GN, María JA, Adriana GV, Irene AC, Alejandra

YM, Janeth BB, María AG. Gut microbiota alterations in critically ill older

patients: a multicenter study. BMC Geriatr. 2022 Apr 28;22(1):373. doi:

10.1186/s12877-022-02981-0. PMID: 35484500; PMCID: PMC9047279.

11: Currie CC, Stone SJ, Brocklehurst P, Slade G, Durham J, Pearce MS. Dental

Attendances to General Medical Practitioners in Wales: A 44 Year-Analysis. J

Dent Res. 2022 Apr;101(4):407-413. doi: 10.1177/00220345211044108. Epub 2021 Sep

28. PMID: 34582311; PMCID: PMC8935529.

12: Beatriz GC, María José O, Inés JL, Yolanda HG, Concha ÁD, Javier TS, Cecilia

M FL. Medication errors in children visiting pediatric emergency departments.

Farm Hosp. 2023 Jul-Aug;47(4):T141-T147. English, Spanish. doi:

10.1016/j.farma.2023.06.001. Epub 2023 Jul 13. PMID: 37453917.

13: Beatriz GC, María José O, Inés JL, Yolanda HG, Concha ÁD, Javier TS, Cecilia

M FL. Medication errors in children visiting pediatric emergency departments.

Farm Hosp. 2023 Jul-Aug;47(4):141-147. English, Spanish. doi:

10.1016/j.farma.2023.03.006. Epub 2023 May 9. PMID: 37164795.

14: Tan CD, Hagedoorn NN, Dewez JE, Borensztajn DM, von Both U, Carrol ED,

Emonts M, van der Flier M, de Groot R, Herberg J, Kohlmaier B, Levin M, Lim E,

Maconochie IK, Martinon-Torres F, Nijman RG, Pokorn M, Rivero-Calle I, Strle F,

Tsolia M, Vermont CL, Yeung S, Zachariasse JM, Zenz W, Zavadska D, Moll HA;

PERFORM Consortium. Rapid Viral Testing and Antibiotic Prescription in Febrile

Children With Respiratory Symptoms Visiting Emergency Departments in Europe.

Pediatr Infect Dis J. 2022 Jan 1;41(1):39-44. doi: 10.1097/INF.0000000000003326.

PMID: 34862345.

15: Otake S, Kusama Y, Tsuzuki S, Myojin S, Kimura M, Kamiyoshi N, Takumi T,

Ishida A, Kasai M. Comparing the effects of antimicrobial stewardship at primary

emergency centers. Pediatr Int. 2023 Jan-Dec;65(1):e15614. doi:

10.1111/ped.15614. PMID: 37658628.

16: Pintado-Álvarez A, Yunquera-Romero L, Márquez-Gómez I, Asensi-Díez R. Does

the use of new cephalosporins follow the authorised, financed and approved

indications? A study of their use in routine clinical practice in a tertiary

hospital. Eur J Hosp Pharm. 2022 Mar;29(e1):e52-e56. doi:

10.1136/ejhpharm-2021-002972. Epub 2021 Dec 21. PMID: 34933886; PMCID:

PMC8899678.

17: Block JP, Bailey LC, Gillman MW, Lunsford D, Boone-Heinonen J, Cleveland LP,

Finkelstein J, Horgan CE, Jay M, Reynolds JS, Sturtevant JL, Forrest CB; PCORnet

Antibiotics Childhood Growth Study Group. PCORnet Antibiotics and Childhood

Growth Study: Process for Cohort Creation and Cohort Description. Acad Pediatr.

2018 Jul;18(5):569-576. doi: 10.1016/j.acap.2018.02.008. Epub 2018 Mar 15. PMID:

29477481; PMCID: PMC9746871.

18: Liang D, Wang ME, Dahlen A, Liao Y, Saunders AC, Coon ER, Schroeder AR.

Incidence of Pediatric Urinary Tract Infections Before and During the COVID-19

Pandemic. JAMA Netw Open. 2024 Jan 2;7(1):e2350061. doi:

10.1001/jamanetworkopen.2023.50061. PMID: 38170521; PMCID: PMC10765266.

19: Okoye BI, Udemba JC, Ndugba CA, Okonkwo JI, Obed EA. Evaluation of rational

prescribing in a hospital paediatric outpatient clinic in Nigeria. BMJ Paediatr

Open. 2022 Oct;6(1):e001585. doi: 10.1136/bmjpo-2022-001585. PMID: 36645753;

PMCID: PMC9562308.

20: Malaeb D, Hallit S, Sacre H, Rahme C, Malaeb B, Hallit R, Salameh P.

Preconception exposure to over-the-counter medications and antibiotics and the

risk of childhood asthma in Lebanon: A cross-sectional study. Allergol

Immunopathol (Madr). 2021 Mar 1;49(2):104-112. doi: 10.15586/aei.v49i2.46. PMID:

33641301.

21: Costenaro P, Cantarutti A, Barbieri E, Scamarcia A, Oletto A, Sacerdoti P,

Lundin R, Cantarutti L, Giaquinto C, Donà D. Antibiotic Prescriptions for

Children With Community-acquired Pneumonia: Findings From Italy. Pediatr Infect

Dis J. 2021 Feb 1;40(2):130-136. doi: 10.1097/INF.0000000000002934. PMID:

33055500.

22: Krueger C, Alqurashi W, Barrowman N, Litwinska M, Le Saux N. The long and

the short of pediatric emergency department antibiotic prescribing: A

retrospective observational study. Am J Emerg Med. 2024 Jan;75:131-136. doi:

10.1016/j.ajem.2023.10.052. Epub 2023 Nov 5. PMID: 37950980.

23: Baraka V, Nhama A, Aide P, Bassat Q, David A, Gesase S, Gwasupika J,

Hachizovu S, Makenga G, Ntizimira CR, Obunge O, Tshefu KA, Cousin M, Otsyula N,

Pathan R, Risterucci C, Su G, Manyando C. Prescription patterns and compliance

with World Health Organization recommendations for the management of

uncomplicated and severe malaria: A prospective, real-world study in sub-Saharan

Africa. Malar J. 2023 Jul 25;22(1):215. doi: 10.1186/s12936-023-04650-y. PMID:

37491295; PMCID: PMC10367305.

24: da Silva RMR, de Mendonça SCB, Leão IN, Dos Santos QN, Batista AM, Melo MS,

Xavier MDM, Quintans Júnior LJ, da Silva WB, Lobo IMF. Use of monitoring

indicators in hospital management of antimicrobials. BMC Infect Dis. 2021 Aug

17;21(1):827. doi: 10.1186/s12879-021-06542-5. PMID: 34404348; PMCID:

PMC8369325.

25: Bronnimann D, Garcia-Hermoso D, Dromer F, Lanternier F; French Mycoses Study

Group; Characterization of the isolates at the NRCMA.

Scedosporiosis/lomentosporiosis observational study (SOS): Clinical significance

of Scedosporium species identification. Med Mycol. 2021 May 4;59(5):486-497.

doi: 10.1093/mmy/myaa086. PMID: 33037432.

26: Gammoh Y, Abdu M. Contact lens procurement and usage habits among adults in

Sudan. PLoS One. 2021 May 19;16(5):e0251987. doi: 10.1371/journal.pone.0251987.

PMID: 34010356; PMCID: PMC8133405.

27: Moore M. Exploring Diagnostic Strategies for Streptococcal Throat Infection

Remotely: A Feasibility Study. Ann Fam Med. 2023 Jan 1;21(21 Suppl 1):4003. doi:

10.1370/afm.21.s1.4003. PMID: 36944082; PMCID: PMC10549346.

28: Thong DW, Kim J, Dobson B, Cheung H, Arthur T; QUEST Collaboration.

Variation in anti-microbial prescription and complications post emergency

appendicectomy in Australia: do we follow recommended guidelines? ANZ J Surg.

2020 Mar;90(3):251-256. doi: 10.1111/ans.15099. Epub 2019 Feb 18. PMID:

30776854.

29: Ashton PM, Chunga Chirambo A, Meiring JE, Patel PD, Mbewe M, Silungwe N,

Chizani K, Banda H, Heyderman RS, Dyson ZA, MacPherson P, Henrion MYR; STRATAA

Study Group; Holt KE, Gordon MA. Evaluating the relationship between

ciprofloxacin prescription and non-susceptibility in Salmonella Typhi in

Blantyre, Malawi: an observational study. Lancet Microbe. 2024

Mar;5(3):e226-e234. doi: 10.1016/S2666-5247(23)00327-0. Epub 2024 Feb 19. PMID:

38387472; PMCID: PMC10914669.

30: Brivio A, Orenti A, Barbisan M, Buonpensiero P, Ros M, Gambazza S. Home

physiotherapists assisting follow-up treatment in cystic fibrosis: a multicenter

observational study. Monaldi Arch Chest Dis. 2021 Apr 15;91(2). doi:

10.4081/monaldi.2021.1619. PMID: 33926178.

31: Chokephaibulkit K, Samant S, Chaisavaneeyakorn S, Kamolratanakul S,

Limpadanai S, Kebede N, Stephens J, Sukarom I, Pawaskar M. Antimicrobial use for

the management of varicella in Thailand: a retrospective observational study.

Curr Med Res Opin. 2023 Jun;39(6):873-880. doi: 10.1080/03007995.2023.2200123.

Epub 2023 Apr 27. PMID: 37057414.

32: Denny KJ, Gartside JG, Alcorn K, Cross JW, Maloney S, Keijzers G.

Appropriateness of antibiotic prescribing in the Emergency Department. J

Antimicrob Chemother. 2019 Feb 1;74(2):515-520. doi: 10.1093/jac/dky447. PMID:

30445465; PMCID: PMC6337898.

33: Pandey S , Yadav CK , Ghimire P , Shrestha AC . Prescription Pattern

Monitoring and Off-label Use of Medicines in the Pediatric Department at

Tertiary Care Teaching Hospital. Kathmandu Univ Med J (KUMJ). 2020

Oct.-Dec.;18(72):367-371. PMID: 34165093.

34: Pierantoni L, Lo Vecchio A, Lenzi J, Corsi V, Campana L, Luca Trobia G,

Amendolea A, Di Felice B, Alighieri G, Fabrizio GC, Bisceglia M, Peia F, Chiale

F, Bartolomei B, Siciliano C, Di Battista C, Passone E, di Giovanni C,

Piergentili E, Donà D, Buonsenso D; on behalf of the Surveys in Pediatric

Infectious Diseases study group. Parents' Perspective of Antibiotic Usage in

Children: A Nationwide Survey in Italy. Pediatr Infect Dis J. 2021 Oct

1;40(10):906-911. doi: 10.1097/INF.0000000000003221. PMID: 34437339.

35: Brugueras S, Orcau À, Millet JP, Espinosa L, de Andrés A, Gorrindo P, Caylà

JA. Tuberculosis clinical units improve contact tracing. Int J Tuberc Lung Dis.

2016 Dec 1;20(12):1572-1579. doi: 10.5588/ijtld.16.0147. PMID: 28000581.

36: Zalla LC, Cole SR, Eron JJ, Adimora AA, Vines AI, Althoff KN, Silverberg MJ,

Horberg MA, Marconi VC, Coburn SB, Lang R, Williams EC, Gill MJ, Gebo KA, Klein

M, Sterling TR, Rebeiro PF, Mayor AM, Moore RD, Edwards JK. Association of Race

and Ethnicity With Initial Prescription of Antiretroviral Therapy Among People

With HIV in the US. JAMA. 2023 Jan 3;329(1):52-62. doi: 10.1001/jama.2022.23617.

PMID: 36594946; PMCID: PMC9856806.

37: Hu L, Fu M, Wushouer H, Ni B, Li H, Guan X, Shi L. The Impact of Sanming

Healthcare Reform on Antibiotic Appropriate Use in County Hospitals in China.

Front Public Health. 2022 Jun 27;10:936719. doi: 10.3389/fpubh.2022.936719.

PMID: 35832279; PMCID: PMC9271699.

38: Boone K, Morris SK, Doshi S, Black J, Mohsin M, Ahmed T, Al Mahmud A, Roth

DE. Antimicrobial Prescribing during Infant Hospital Admissions in a Birth

Cohort in Dhaka, Bangladesh. J Trop Pediatr. 2021 Jul 2;67(3):fmaa093. doi:

10.1093/tropej/fmaa093. PMID: 33221898; PMCID: PMC8319631.

39: Hughes S, Heard K, Mughal N, Moore LSP. Burden of enteral supplement

interactions with common antimicrobial agents: a single-centre observational

analysis. Eur J Hosp Pharm. 2022 Sep;29(5):280-283. doi:

10.1136/ejhpharm-2020-002445. Epub 2021 Jan 7. PMID: 33414257; PMCID:

PMC9660598.

40: Chang CT, Liu SP, Muo CH, Tsai CH, Huang YF. Dental Prophylaxis and

Osteoradionecrosis: A Population-Based Study. J Dent Res. 2017

May;96(5):531-538. doi: 10.1177/0022034516687282. Epub 2017 Jan 17. PMID:

28095728.

41: Chávez-Íñiguez JS, Maggiani-Aguilera P, Pérez-Flores C, Claure-Del Granado

R, De la Torre-Quiroga AE, Martínez-Gallardo González A, Navarro-Blackaller G,

Medina-González R, Raimann JG, Yanowsky-Escatell FG, García-García G.

Nephrologist Interventions to Avoid Kidney Replacement Therapy in Acute Kidney

Injury. Kidney Blood Press Res. 2021;46(5):629-638. doi: 10.1159/000517615. Epub

2021 Jul 27. PMID: 34315155.

42: Miao R, Wan C, Wang Z, Zhu Y, Zhao Y, Zhang L, Liu J, Qin J, Xia J, Yan H.

Inappropriate antibiotic prescriptions among pediatric inpatients in different

type hospitals. Medicine (Baltimore). 2020 Jan;99(2):e18714. doi:

10.1097/MD.0000000000018714. PMID: 31914082; PMCID: PMC6959949.

43: Zver S, Avcin S, Bedreag O, Bizilj S, Ecrulj V, Jazbec J, Puconja N, Stanic

R, Jereb M. Micafungin for Candida infections in Slovenia and Romania: A

multicenter, observational, prospective study. J Infect Dev Ctries. 2021 Jun

30;15(6):877-888. doi: 10.3855/jidc.12755. PMID: 34242200.

44: Garioud ALB, Skoven FH, Gregersen R, Lange T, Buchvald F, Greisen G. The

increased susceptibility to airway infections after preterm birth does not

persist into adolescence. PLoS One. 2020 Sep 3;15(9):e0238382. doi:

10.1371/journal.pone.0238382. Erratum in: PLoS One. 2020 Dec 31;15(12):e0244952.

doi: 10.1371/journal.pone.0244952. PMID: 32881953; PMCID: PMC7470365.

45: Korppi M, Heikkilä P, Palmu S, Huhtala H, Csonka P. Antibiotic prescribing

for children with upper respiratory tract infection: a Finnish nationwide 7-year

observational study. Eur J Pediatr. 2022 Aug;181(8):2981-2990. doi:

10.1007/s00431-022-04512-w. Epub 2022 May 23. PMID: 35606593; PMCID: PMC9126572.

46: Gonzalez ML, Aristizabal P, Loera-Reyna A, Torres D, Ornelas-Sánchez M,

Nuño-Vázquez L, Aguilera M, Sánchez A, Romano M, Rivera-Gómez R, Relyea G,

Friedrich P, Caniza MA. The Golden Hour: Sustainability and Clinical Outcomes of

Adequate Time to Antibiotic Administration in Children with Cancer and Febrile

Neutropenia in Northwestern Mexico. JCO Glob Oncol. 2021 May;7:659-670. doi:

10.1200/GO.20.00578. PMID: 33974443; PMCID: PMC8162497.

47: Cizeron A, Saunier F, Gagneux-Brunon A, Pillet S, Cantais A, Botelho-Nevers

E. Low rate of oseltamivir prescription among adults and children with confirmed

influenza illness in France during the 2018-19 influenza season. J Antimicrob

Chemother. 2021 Mar 12;76(4):1057-1062. doi: 10.1093/jac/dkaa539. PMID:

33406225.

48: Milic J, Novella A, Meschiari M, Menozzi M, Santoro A, Bedini A, Cuomo G,

Franceschini E, Digaetano M, Carli F, Ciusa G, Volpi S, Bacca E, Franceschi G,

Yaacoub D, Rogati C, Tutone M, Burastero G, Faltoni M, Iadisernia V, Dolci G,

Cossarizza A, Mussini C, Pasina L, Guaraldi G. Darunavir/Cobicistat Is

Associated with Negative Outcomes in HIV-Negative Patients with Severe COVID-19

Pneumonia. AIDS Res Hum Retroviruses. 2021 Apr;37(4):283-291. doi:

10.1089/AID.2020.0305. PMID: 33619997.

49: Sutton SS, Hyche S, Magagnoli J, Hardin JW. Appraisal of the cardiovascular

risks of azithromycin: an observational analysis. J Comp Eff Res. 2017

Sep;6(6):509-517. doi: 10.2217/cer-2016-0080. Epub 2017 Sep 29. PMID: 28960092.

50: Stern JA, Beijers R, Ehrlich KB, Cassidy J, de Weerth C. Beyond Early

Adversity: The Role of Parenting in Infant Physical Health. J Dev Behav Pediatr.

2020 Aug;41(6):452-460. doi: 10.1097/DBP.0000000000000804. PMID: 32271266;

PMCID: PMC9364161.

51: Clark AW, Durkin MJ, Olsen MA, Keller M, Ma Y, O'Neil CA, Butler AM. Rural-

urban differences in antibiotic prescribing for uncomplicated urinary tract

infection. Infect Control Hosp Epidemiol. 2021 Dec;42(12):1437-1444. doi:

10.1017/ice.2021.21. Epub 2021 Feb 24. PMID: 33622432; PMCID: PMC8382778.

52: Manen S, Bost-Bru C, Wroblewski I, De Crescenzo M, Mortamet G.

Aminoglycoside prescription: compliance with national guidelines in a pediatric

hospital. Arch Pediatr. 2021 Oct;28(7):583-586. doi:

10.1016/j.arcped.2021.06.003. Epub 2021 Aug 13. PMID: 34393024.

53: Hullegie S, Schilder AGM, Marchisio P, de Sévaux JLH, van der Velden AW, van

de Pol AC, Boeijen JA, Platteel TN, Torretta S, Damoiseaux RAMJ, Venekamp RP. A

Strong Decline in the Incidence of Childhood Otitis Media During the COVID-19

Pandemic in the Netherlands. Front Cell Infect Microbiol. 2021 Nov 1;11:768377.

doi: 10.3389/fcimb.2021.768377. PMID: 34790591; PMCID: PMC8591181.

54: Roblin DW, Liu H, Cromwell LF, Robbins M, Robinson BE, Auerbach D, Mehrotra

A. Provider type and management of common visits in primary care. Am J Manag

Care. 2017 Apr;23(4):225-231. PMID: 28554207.

55: van Aerde KJ, de Haan L, van Leur M, Gerrits GP, Schers H, Moll HA,

Hagedoorn NN, Herberg JA, Levin M, Rivero-Calle I, de Jonge MI, de Groot R, van

der Flier M; PERFORM Consortium. Respiratory Tract Infection Management and

Antibiotic Prescription in Children: A Unique Study Comparing Three Levels of

Healthcare in The Netherlands. Pediatr Infect Dis J. 2021 Mar 1;40(3):e100-e105.

doi: 10.1097/INF.0000000000003019. Erratum in: Pediatr Infect Dis J. 2022 Mar

1;41(3):274. doi: 10.1097/INF.0000000000003427. PMID: 33395212.

56: Dassner AM, Girotto JE. Evaluation of a Second-Sign Process for

Antimicrobial Prior Authorization. J Pediatric Infect Dis Soc. 2018 May

15;7(2):113-118. doi: 10.1093/jpids/pix015. PMID: 28407067.

57: Wathne JS, Harthug S, Kleppe LKS, Blix HS, Nilsen RM, Charani E, Smith I.

The association between adherence to national antibiotic guidelines and

mortality, readmission and length of stay in hospital inpatients: results from a

Norwegian multicentre, observational cohort study. Antimicrob Resist Infect

Control. 2019 Apr 15;8:63. doi: 10.1186/s13756-019-0515-5. PMID: 31011417;

PMCID: PMC6466722.

58: Di Mario S, Gagliotti C, Buttazzi R, Marchetti F, Dodi I, Barbieri L, Moro

ML. Reducing antibiotic prescriptions in children is not associated with higher

rate of complications. Eur J Pediatr. 2021 Apr;180(4):1185-1192. doi:

10.1007/s00431-020-03861-8. Epub 2020 Nov 3. PMID: 33145703.

59: Peña-Vélez R, Dzul-Pech FM, Salgado-Valencia J, Calva R, Gil-Vargas M. Acute

pancreatitis in children and adolescents: diagnostic and therapeutic approach

according to management guidelines in a group of pediatricians. Bol Med Hosp

Infant Mex. 2024;81(2):85-89. English. doi: 10.24875/BMHIM.22000157. PMID:

38768511.

60: Rastogi R, Martinez KA, Gupta N, Rood M, Rothberg MB. Management of Urinary

Tract Infections in Direct to Consumer Telemedicine. J Gen Intern Med. 2020

Mar;35(3):643-648. doi: 10.1007/s11606-019-05415-7. Epub 2019 Oct 30. PMID:

31667749; PMCID: PMC7080949.

61: Eijsvoogel NB, Verstegen RHJ, van Well GTJ, van Hout RWNM, de Vries E.

Increased rate of respiratory symptoms in children with Down syndrome: a 2-year

web-based parent-reported prospective study. Eur J Pediatr. 2022

Dec;181(12):4079-4089. doi: 10.1007/s00431-022-04634-1. Epub 2022 Oct 3. PMID:

36192547; PMCID: PMC9649482.

62: Fominykh V, Averchenkov D, Volik A, Popova E, Bryukhov V, Nazarov V,

Moshnikova A, Arzumanian N, Tatarenko A, Nechaev V, Manuylova O, Lapin S, Brylev

L, Guekht A. Levamisole-associated multifocal inflammatory encephalopathy:

clinical and MRI characteristics, and diagnostic algorithm. Mult Scler Relat

Disord. 2023 Jan;69:104418. doi: 10.1016/j.msard.2022.104418. Epub 2022 Nov 17.

PMID: 36450175.

63: Mendoza-Palomar N, Garcia-Palop B, Melendo S, Martín MT, Renedo-Miró B,

Soler-Palacin P, Fernández-Polo A. Antifungal stewardship in a tertiary care

paediatric hospital: the PROAFUNGI study. BMC Infect Dis. 2021 Jan 22;21(1):100.

doi: 10.1186/s12879-021-05774-9. PMID: 33482749; PMCID: PMC7821674.

64: Tan CD, van der Walle EEPL, Vermont CL, von Both U, Carrol ED, Eleftheriou

I, Emonts M, van der Flier M, de Groot R, Herberg J, Kohlmaier B, Levin M, Lim

E, Maconochie IK, Martinon-Torres F, Nijman RG, Pokorn M, Rivero-Calle I, Tsolia

M, Yeung S, Zenz W, Zavadska D, Moll HA; PERFORM consortium (Personalised Risk

assessment in febrile children to optimize Real-life Management across the

European Union). Guideline adherence in febrile children below 3 months visiting

European Emergency Departments: an observational multicenter study. Eur J

Pediatr. 2022 Dec;181(12):4199-4209. doi: 10.1007/s00431-022-04606-5. Epub 2022

Sep 30. Erratum in: Eur J Pediatr. 2022 Dec;181(12):4211-4214. doi:

10.1007/s00431-022-04664-9. PMID: 36178539; PMCID: PMC9649464.

65: Yi S, Ramachandran A, Epps L, Mayah A, Burkholder TW, Jaung MS, Haider A,

Whesseh P, Shakpeh J, Enriquez K, Bills C. Emergency department antimicrobial

use in a low-resource setting: results from a retrospective observational study

at a referral hospital in Liberia. BMJ Open. 2022 Apr 18;12(4):e056709. doi:

10.1136/bmjopen-2021-056709. PMID: 35437249; PMCID: PMC9016394.

66: Robert S, Ménétré S, Schweitzer C, Demoré B. Observational study of drug-

related problems and clinical pharmacists' interventions in a French paediatric

hospital. Eur J Hosp Pharm. 2021 Nov;28(Suppl 2):e85-e91. doi:

10.1136/ejhpharm-2020-002319. Epub 2020 Oct 28. PMID: 33115799; PMCID:

PMC8640428.

67: Hodgson KA, Huynh J, Ibrahim LF, Sacks B, Golshevsky D, Layley M, Spagnolo

M, Raymundo CM, Bryant PA. The use, appropriateness and outcomes of outpatient

parenteral antimicrobial therapy. Arch Dis Child. 2016 Oct;101(10):886-93. doi:

10.1136/archdischild-2015-309731. Epub 2016 May 10. PMID: 27166221.

68: MacKenzie EL, Murillo C, Bartlett AH, Marrs R, Landon EM, Ridgway JP.

<i>Clostridioides difficile</i> colonization and the frequency of subsequent

treatment for <i>C. difficile</i> infection in critically ill patients. Infect

Control Hosp Epidemiol. 2023 Nov;44(11):1782-1787. doi: 10.1017/ice.2022.240.

Epub 2023 Jan 20. PMID: 36658099.

69: Agarwal A, Chakma N, Manchanda V, Dabas A. Virological profile of upper

respiratory tract infections in children under 5 years of age- a cross sectional

study in a tertiary care hospital in North India. Indian J Med Microbiol. 2023

Jul-Aug;44:100378. doi: 10.1016/j.ijmmb.2023.100378. Epub 2023 May 5. PMID:

37356837.

70: Alrabiah Z, Arafah A, Rehman MU, Babelghaith SD, Syed W, Alrashidi FK,

Aldajaani FF, Alsufayan MA, Arifi MNA. Prevalence and Self-Medication for Acne

among Students of Health-Related Science Colleges at King Saud University in

Riyadh Region Saudi Arabia. Medicina (Kaunas). 2022 Dec 27;59(1):52. doi:

10.3390/medicina59010052. PMID: 36676676; PMCID: PMC9863386.

71: van de Maat J, van de Voort E, Mintegi S, Gervaix A, Nieboer D, Moll H,

Oostenbrink R; Research in European Pediatric Emergency Medicine study group.

Antibiotic prescription for febrile children in European emergency departments:

a cross-sectional, observational study. Lancet Infect Dis. 2019

Apr;19(4):382-391. doi: 10.1016/S1473-3099(18)30672-8. Epub 2019 Feb 28. PMID:

30827808.

72: Benevent J, Hurault-Delarue C, Araujo M, Montastruc JL, Lacroix I, Damase-

Michel C. POMME: The New Cohort to Evaluate Long-Term Effects After Prenatal

Medicine Exposure. Drug Saf. 2019 Jan;42(1):45-54. doi:

10.1007/s40264-018-0712-9. PMID: 30121741.

73: Picca M, Carrozzo R, Milani GP, Corsello A, Macchi M, Buzzetti R, Marchisio

P, Mameli C. Leading reasons for antibiotic prescriptions in pediatric

respiratory infections: influence of fever in a primary care setting. Ital J

Pediatr. 2023 Sep 29;49(1):131. doi: 10.1186/s13052-023-01533-5. PMID: 37775784;

PMCID: PMC10541709.

74: Mounzer KC, Fusco JS, Hsu RK, Brunet L, Vannappagari V, Frost KR, Shaefer

MS, Rinehart A, Rawlings K, Fusco GP. Are We Hitting the Target? HIV Pre-

Exposure Prophylaxis from 2012 to 2020 in the OPERA Cohort. AIDS Patient Care

STDS. 2021 Nov;35(11):419-427. doi: 10.1089/apc.2021.0064. Epub 2021 Oct 4.

PMID: 34609897.

75: Lalande J, Vrignaud B, Navas D, Levieux K, Herbreteau B, Guillou A, Gras-Le

Guen C, Launay E. A prospective observational study of medication errors in a

pediatric emergency department. Arch Pediatr. 2018 Aug;25(6):355-358. doi:

10.1016/j.arcped.2018.06.005. Epub 2018 Jul 29. PMID: 30064711.

76: Campitelli MA, Bronskill SE, Maclagan LC, Harris DA, Cotton CA, Tadrous M,

Gruneir A, Hogan DB, Maxwell CJ. Comparison of Medication Prescribing Before and

After the COVID-19 Pandemic Among Nursing Home Residents in Ontario, Canada.

JAMA Netw Open. 2021 Aug 2;4(8):e2118441. doi:

10.1001/jamanetworkopen.2021.18441. PMID: 34338794; PMCID: PMC8329744.

77: Shu Z, Zhou Y, Chang K, Liu J, Min X, Zhang Q, Sun J, Xiong Y, Zou Q, Zheng

Q, Ji J, Poon J, Liu B, Zhou X, Li X. Clinical features and the traditional

Chinese medicine therapeutic characteristics of 293 COVID-19 inpatient cases.

Front Med. 2020 Dec;14(6):760-775. doi: 10.1007/s11684-020-0803-8. Epub 2020 Sep

14. PMID: 32926319; PMCID: PMC7488634.

78: Naito T, Mori H, Fujibayashi K, Fukushima S, Yuda M, Fukui N, Tsukamoto S,

Suzuki M, Goto-Hirano K, Kuwatsuru R. Analysis of antiretroviral therapy switch

rate and switching pattern for people living with HIV from a national database

in Japan. Sci Rep. 2022 Feb 2;12(1):1732. doi: 10.1038/s41598-022-05816-5. PMID:

35110641; PMCID: PMC8810755.

79: Mehra A, Semwal P, Bhat NK, Bolia R. A Prospective Observational Study of

Hepatic Dysfunction in Children on Antitubercular Drugs. Indian J Pediatr. 2022

Nov;89(11):1126-1128. doi: 10.1007/s12098-022-04317-7. Epub 2022 Jul 22. PMID:

35867272.

80: Kenga DB, Gebretsadik T, Simbine S, Maússe FE, Charles P, Zaqueu E, Fernando

HF, Manjate A, Sacarlal J, Moon TD. Community-acquired bacteremia among HIV-

infected and HIV-exposed uninfected children hospitalized with fever in

Mozambique. Int J Infect Dis. 2021 Aug;109:99-107. doi:

10.1016/j.ijid.2021.06.047. Epub 2021 Jun 24. PMID: 34174435; PMCID: PMC8437032.

81: Duncan ADS, Hapca S, De Souza N, Morales D, Bell S. Quinine exposure and the

risk of acute kidney injury: a population-based observational study of older

people. Age Ageing. 2020 Oct 23;49(6):1042-1047. doi: 10.1093/ageing/afaa079.

PMID: 32463438; PMCID: PMC7583521.

82: Norikoshi Y, Ikeda T, Sasahara K, Hamada M, Torigoe E, Nagae M, Tashiro T,

Horio F, Saruwatari J, Uchida Y, Anraku M. A Comparison of the Frequency of

Prescription and Pharmacy Revisits between Baloxavir Marboxil and Neuraminidase

Inhibitors in Influenza-Infected Pediatric Patients during the 2019-2020

Influenza Season. Biol Pharm Bull. 2020;43(12):1960-1965. doi:

10.1248/bpb.b20-00543. PMID: 33268716.

83: Thomson KM, Dyer C, Liu F, Sands K, Portal E, Carvalho MJ, Barrell M,

Boostrom I, Dunachie S, Farzana R, Ferreira A, Frayne F, Hassan B, Jones E,

Jones L, Mathias J, Milton R, Rees J, Chan GJ, Bekele D, Mahlet A, Basu S, Nandy

RK, Saha B, Iregbu K, Modibbo F, Uwaezuoke S, Zahra R, Shirazi H, Syed NU,

Mazarati JB, Rucogoza A, Gaju L, Mehtar S, Bulabula ANH, Whitelaw A, van Hasselt

JGC, Walsh TR; BARNARDS Group. Effects of antibiotic resistance, drug target

attainment, bacterial pathogenicity and virulence, and antibiotic access and

affordability on outcomes in neonatal sepsis: an international microbiology and

drug evaluation prospective substudy (BARNARDS). Lancet Infect Dis. 2021

Dec;21(12):1677-1688. doi: 10.1016/S1473-3099(21)00050-5. Epub 2021 Aug 9. PMID:

34384533; PMCID: PMC8612937.

84: Ali AM, Mohamed AN, Mohamed YG, Keleşoğlu Sİ. Clinical presentation and

surgical management of perforated peptic ulcer in a tertiary hospital in

Mogadishu, Somalia: a 5-year retrospective study. World J Emerg Surg. 2022 May

16;17(1):23. doi: 10.1186/s13017-022-00428-w. PMID: 35578285; PMCID: PMC9112500.

85: Braeken DCW, Spruit MA, Houben-Wilke S, Smid DE, Rohde GGU, Wouters EFM,

Franssen FME. Impact of exacerbations on adherence and outcomes of pulmonary

rehabilitation in patients with COPD. Respirology. 2017 Jul;22(5):942-949. doi:

10.1111/resp.12987. Epub 2017 Jan 31. PMID: 28139873.

86: Williams MR, Greene G, Naik G, Hughes K, Butler CC, Hay AD. Antibiotic

prescribing quality for children in primary care: an observational study. Br J

Gen Pract. 2018 Feb;68(667):e90-e96. doi: 10.3399/bjgp18X694409. Epub 2018 Jan

15. PMID: 29335323; PMCID: PMC5774968.

87: Brauer R, Wong ICK, Man KK, Pratt NL, Park RW, Cho SY, Li YJ, Iqbal U,

Nguyen PA, Schuemie M. Application of a Common Data Model (CDM) to rank the

paediatric user and prescription prevalence of 15 different drug classes in

South Korea, Hong Kong, Taiwan, Japan and Australia: an observational,

descriptive study. BMJ Open. 2020 Jan 13;10(1):e032426. doi:

10.1136/bmjopen-2019-032426. PMID: 31937652; PMCID: PMC7044847.

88: Spigelmyer A, Howard C, Rybakov I, Burwell S, Slain D. Impact of clinical

pharmacist discharge prescription review on the appropriateness of antibiotic

therapy: a retrospective comparison. Int J Clin Pharm. 2023 Jun;45(3):769-773.

doi: 10.1007/s11096-022-01503-7. Epub 2022 Nov 23. PMID: 36418632; PMCID:

PMC10250257.

89: Iskandar K, Hanna PA, Salameh P, Raad EB. Antibiotic consumption in non-

teaching Lebanese hospitals: A cross-sectional study. J Infect Public Health.

2016 Sep-Oct;9(5):618-25. doi: 10.1016/j.jiph.2015.12.013. Epub 2016 Jan 21.

PMID: 26806876.

90: Tsuzuki S, Kimura Y, Ishikane M, Kusama Y, Ohmagari N. Cost of inappropriate

antimicrobial use for upper respiratory infection in Japan. BMC Health Serv Res.

2020 Feb 28;20(1):153. doi: 10.1186/s12913-020-5021-1. PMID: 32111202; PMCID:

PMC7048145.

91: Pinto SM, Twichell MF, Henry LC. Predictors of Pharmacological Intervention

in Adolescents With Protracted Symptoms After Sports-Related Concussion. PM R.

2017 Sep;9(9):847-855. doi: 10.1016/j.pmrj.2016.12.009. Epub 2017 Jan 14. PMID:

28093374.

92: Kusturica MP, Tomic Z, Bukumiric Z, Ninkovic L, Tomas A, Stilinovic N, Sabo

A. Home pharmacies in Serbia: an insight into self-medication practice. Int J

Clin Pharm. 2015 Apr;37(2):373-8. doi: 10.1007/s11096-015-0071-x. Epub 2015 Jan

24. PMID: 25616627.

93: Devillers L, Sicsic J, Delbarre A, Le Bel J, Ferrat E, Saint Lary O. General

Practitioner trainers prescribe fewer antibiotics in primary care: Evidence from

France. PLoS One. 2018 Jan 25;13(1):e0190522. doi: 10.1371/journal.pone.0190522.

PMID: 29370178; PMCID: PMC5784911.

94: Soo JEJ, Chan MY, Bte Adb Rashid NAB, Bte Mohamad Yusri LI, Wynn YY, Noda M,

Tewani K. Medication chart review at end of life of paediatric palliative

patients. J Paediatr Child Health. 2022 Mar;58(3):392-396. doi:

10.1111/jpc.15719. Epub 2021 Sep 23. PMID: 34553811.

95: Taylor S, Taylor RJ, Lustig RL, Schuck-Paim C, Haguinet F, Webb DJ, Logie J,

Matias G, Fleming DM. Modelling estimates of the burden of respiratory syncytial

virus infection in children in the UK. BMJ Open. 2016 Jun 2;6(6):e009337. doi:

10.1136/bmjopen-2015-009337. PMID: 27256085; PMCID: PMC4893852.

96: Barbieri E, Donà D, Cantarutti A, Lundin R, Scamarcia A, Corrao G,

Cantarutti L, Giaquinto C. Antibiotic prescriptions in acute otitis media and

pharyngitis in Italian pediatric outpatients. Ital J Pediatr. 2019 Aug

17;45(1):103. doi: 10.1186/s13052-019-0696-9. PMID: 31420054; PMCID: PMC6697973.

97: Okubo Y, Nariai H, Michels KB, Kim-Farley RJ, Nishi A, Arah OA, Kinoshita N,

Uda K, Miyairi I. Change in clinical practice variations for antibiotic

prescriptions across different pediatric clinics: A Japan's nationwide

observational study. J Infect Chemother. 2021 Nov;27(11):1621-1625. doi:

10.1016/j.jiac.2021.07.020. Epub 2021 Aug 8. PMID: 34376349.

98: Watson JR, Wang L, Klima J, Moore-Clingenpeel M, Gleeson S, Kelleher K,

Jaggi P. Healthcare Claims Data: An Underutilized Tool for Pediatric Outpatient

Antimicrobial Stewardship. Clin Infect Dis. 2017 Jun 1;64(11):1479-1485. doi:

10.1093/cid/cix195. Erratum in: Clin Infect Dis. 2017 Oct 15;65(8):1431-1433.

doi: 10.1093/cid/cix563. PMID: 28329388.

99: Skender K, Singh V, Stalsby-Lundborg C, Sharma M. Trends and patterns of

antibiotic prescribing at orthopedic inpatient departments of two private-sector

hospitals in Central India: A 10-year observational study. PLoS One. 2021 Jan

27;16(1):e0245902. doi: 10.1371/journal.pone.0245902. PMID: 33503028; PMCID:

PMC7840031.

100: Sabry NA, Farid SF, Dawoud DM. Antibiotic dispensing in Egyptian community

pharmacies: an observational study. Res Social Adm Pharm. 2014 Jan-

Feb;10(1):168-84. doi: 10.1016/j.sapharm.2013.03.004. Epub 2013 May 10. PMID:

23665078.

101: Kissler SM, Wang B, Mehrotra A, Barnett M, Grad YH. Impact of Respiratory

Infection and Chronic Comorbidities on Early Pediatric Antibiotic Dispensing in

the United States. Clin Infect Dis. 2023 Feb 8;76(3):382-388. doi:

10.1093/cid/ciac811. PMID: 36196577; PMCID: PMC9907510.

102: Flannery DD, Passarella M, Mukhopadhyay S, Dhudasia MB, Gerber JS, Lorch

SA, Hennessy S, Puopolo KM. Early childhood antibiotic utilization for infants

discharged from the neonatal intensive care unit. J Perinatol. 2022

Jul;42(7):953-958. doi: 10.1038/s41372-022-01380-y. PMID: 35383276; PMCID:

PMC9262761.

103: Christensen MB, Nørøxe KB, Moth G, Vedsted P, Huibers L. Drug prescriptions

in Danish out-of-hours primary care: a 1-yearpopulation-based study. Scand J

Prim Health Care. 2016 Dec;34(4):453-458. doi: 10.1080/02813432.2016.1248622.

Epub 2016 Nov 2. PMID: 27804314; PMCID: PMC5217277.

104: de Witte LD, Munk Laursen T, Corcoran CM, Munk-Olsen T, Bergink V.

Association between doxycycline use and long-term functioning in patients with

schizophrenia. Brain Behav Immun. 2024 Mar;117:66-69. doi:

10.1016/j.bbi.2023.12.036. Epub 2023 Dec 31. PMID: 38169245; PMCID: PMC10932900.

105: Mundkur ML, Franklin J, Huybrechts KF, Fischer MA, Kesselheim AS, Linder

JA, Landon J, Patorno E. Changes in Outpatient Use of Antibiotics by Adults in

the United States, 2006-2015. Drug Saf. 2018 Dec;41(12):1333-1342. doi:

10.1007/s40264-018-0697-4. PMID: 29987757.

106: Malo S, José Rabanaque M, Feja C, Jesús Lallana M, Aguilar I, Bjerrum L.

High antibiotic consumption: a characterization of heavy users in Spain. Basic

Clin Pharmacol Toxicol. 2014 Sep;115(3):231-6. doi: 10.1111/bcpt.12211. Epub

2014 Mar 7. PMID: 24517562.

107: Ivanovska V, Hek K, Mantel Teeuwisse AK, Leufkens HG, Nielen MM, van Dijk

L. Antibiotic prescribing for children in primary care and adherence to

treatment guidelines. J Antimicrob Chemother. 2016 Jun;71(6):1707-14. doi:

10.1093/jac/dkw030. Epub 2016 Mar 5. PMID: 26945710.

108: Muraki Y, Kusama Y, Tanabe M, Hayakawa K, Gu Y, Ishikane M, Yamasaki D,

Yagi T, Ohmagari N. Impact of antimicrobial stewardship fee on prescribing for

Japanese pediatric patients with upper respiratory infections. BMC Health Serv

Res. 2020 May 11;20(1):399. doi: 10.1186/s12913-020-05288-1. PMID: 32393267;

PMCID: PMC7212615.

109: Meier GC, Watkins J, McEwan P, Pockett RD. Resource use and direct medical

costs of acute respiratory illness in the UK based on linked primary and

secondary care records from 2001 to 2009. PLoS One. 2020 Aug 6;15(8):e0236472.

doi: 10.1371/journal.pone.0236472. PMID: 32760071; PMCID: PMC7410242.

110: Hagen TL, Hertz MA, Uhrin GB, Dalager-Pedersen M, Schønheyder HC, Nielsen

H. Adherence to local antimicrobial guidelines for initial treatment of

community-acquired infections. Dan Med J. 2017 Jun;64(6):A5381. PMID: 28566116.

111: Siffel C, Hennies N, Joseph C, Lascano V, Horvat P, Scheider M, Ganzera F.

Burden of dry eye disease in Germany: a retrospective observational study using

German claims data. Acta Ophthalmol. 2020 Jun;98(4):e504-e512. doi:

10.1111/aos.14300. Epub 2019 Nov 17. PMID: 31736282.

112: Ekwochi U, Chinawa JM, Osuorah CD, Odetunde OI, Obu HA, Agwu S. The use of

unprescribed antibiotics in management of upper respiratory tract infection in

children in Enugu, South East Nigeria. J Trop Pediatr. 2014 Jun;60(3):249-52.

doi: 10.1093/tropej/fmt111. Epub 2014 Jan 15. PMID: 24436341.

113: Law AV, Sakharkar P, Zargarzadeh A, Tai BW, Hess K, Hata M, Mireles R, Ha

C, Park TJ. Taking stock of medication wastage: Unused medications in US

households. Res Social Adm Pharm. 2015 Jul-Aug;11(4):571-8. doi:

10.1016/j.sapharm.2014.10.003. Epub 2014 Oct 17. PMID: 25487420.

114: Giacobbe DR, Saffioti C, Losito AR, Rinaldi M, Aurilio C, Bolla C, Boni S,

Borgia G, Carannante N, Cassola G, Ceccarelli G, Corcione S, Dalla Gasperina D,

De Rosa FG, Dentone C, Di Bella S, Di Lauria N, Feasi M, Fiore M, Fossati S,

Franceschini E, Gori A, Granata G, Grignolo S, Grossi PA, Guadagnino G, Lagi F,

Maraolo AE, Marinò V, Mazzitelli M, Mularoni A, Oliva A, Pace MC, Parisini A,

Patti F, Petrosillo N, Pota V, Raffaelli F, Rossi M, Santoro A, Tascini C, Torti

C, Trecarichi EM, Venditti M, Viale P, Signori A, Bassetti M, Del Bono V,

Giannella M, Mikulska M, Tumbarello M, Viscoli C; SITA GIOVANI (Young

Investigators Group of the Società Italiana Terapia Antinfettiva) and the COLI-

CROSS Study Group. Use of colistin in adult patients: A cross-sectional study. J

Glob Antimicrob Resist. 2020 Mar;20:43-49. doi: 10.1016/j.jgar.2019.06.009. Epub

2019 Jun 15. PMID: 31207379.

115: Li C, Ong C, Morris A, Woollons I, Ashfaq A, Jagatia R. Evaluating the

Appropriateness of Antibiotic Treatment of Tonsillitis during COVID-19 in the

North Wale Primary Healthcare Setting. J Prim Care Community Health. 2021 Jan-

Dec;12:21501327211003687. doi: 10.1177/21501327211003687. PMID: 33733905; PMCID:

PMC7983464.

116: Sugita H, Okada N, Okamoto M, Abe M, Sekido M, Tanaka M, Tamatukuri T,

Naito Y, Yoshikawa M, Inoue E, Tanaka H. Evaluation of the initial timing of

infection control pharmacist-driven audit and monitoring of vancomycin therapy

in patients with infectious diseases: A retrospective observational study. PLoS

One. 2023 Aug 31;18(8):e0291096. doi: 10.1371/journal.pone.0291096. PMID:

37651455; PMCID: PMC10470910.

117: Brestrich G, Hagemann C, Diesing J, Kossack N, Stark JH, Pilz A, Angulo FJ,

Yu H, Suess J. Incidence of Lyme Borreliosis in Germany: A retrospective

observational healthcare claims study. Ticks Tick Borne Dis. 2024

May;15(3):102326. doi: 10.1016/j.ttbdis.2024.102326. Epub 2024 Feb 27. PMID:

38417196.

118: De Rop L, De Burghgraeve T, De Sutter A, Buntinx F, Verbakel JY. Point-of-

care C-reactive protein test results in acute infections in children in primary

care: an observational study. BMC Pediatr. 2022 Nov 4;22(1):633. doi:

10.1186/s12887-022-03677-5. PMID: 36333682; PMCID: PMC9635070.

119: Koyama T, Hagiya H, Teratani Y, Tatebe Y, Ohshima A, Adachi M, Funahashi T,

Zamami Y, Tanaka HY, Tasaka K, Shinomiya K, Kitamura Y, Sendo T, Hinotsu S, Kano

MR. Antibiotic prescriptions for Japanese outpatients with acute respiratory

tract infections (2013-2015): A retrospective Observational Study. J Infect

Chemother. 2020 Jul;26(7):660-666. doi: 10.1016/j.jiac.2020.02.001. Epub 2020

Mar 12. PMID: 32173283.

120: Fink G, D'Acremont V, Leslie HH, Cohen J. Antibiotic exposure among

children younger than 5 years in low-income and middle-income countries: a

cross-sectional study of nationally representative facility-based and household-

based surveys. Lancet Infect Dis. 2020 Feb;20(2):179-187. doi:

10.1016/S1473-3099(19)30572-9. Epub 2019 Dec 13. PMID: 31843383.

121: de Barros Fernandes T, Ramos SF, Leitzke LRF, Júnior RGA, de Araújo JM, de

Souza Júnior AS, da Silva ARO, Heineck I, de França Fonteles MM, Bracken LE,

Peak M, de Lyra Junior DP, Osorio-de-Castro CGS, Lima EC. Use of antimicrobials

in pediatric wards of five Brazilian hospitals. BMC Pediatr. 2024 Mar

13;24(1):177. doi: 10.1186/s12887-024-04655-9. PMID: 38481225; PMCID:

PMC10936065.

122: Karinauske E, Kasciuskeviciute S, Morkuniene V, Garuoliene K, Kadusevicius

E. Antibiotic prescribing trends in a pediatric population in Lithuania in

2003-2012: Observational study. Medicine (Baltimore). 2019 Nov;98(46):e17220.

doi: 10.1097/MD.0000000000017220. PMID: 31725600; PMCID: PMC6867790.

123: Momenzadeh A, Shumway M, Dong BJ, Dilley J, Nye J, Mangurian C. Patterns of

Prescribing Antiretroviral Therapy Upon Discharge to Psychiatry Inpatients With

HIV/AIDS at a Large Urban Hospital. Ann Pharmacother. 2021 Apr;55(4):452-458.

doi: 10.1177/1060028020954924. Epub 2020 Sep 4. PMID: 32885983.

124: Coleman BL, Hassan K, Green K, Gubbay JB, Katz K, Mazzulli T, McNeil S,

Muller M, Powis J, Richardson D, Simor A; TIBDN; McGeer AJ. Pre-and post-

pandemic trends in antiviral use in hospitalized patients with laboratory-

confirmed influenza: 2004/05-2013/14, Toronto, Canada. Antiviral Res. 2017

Apr;140:158-163. doi: 10.1016/j.antiviral.2017.01.025. Epub 2017 Feb 6. PMID:

28179155.

125: Godoy BZ, Faintuch J, Marin ML, Nogueira MA, Pinto VB, Pollara WM. Off

label pharmacological therapy in patients with short bowel syndrome. Eur Rev Med

Pharmacol Sci. 2013 Dec;17(24):3285-90. PMID: 24379057.

126: Cassy A, Saifodine A, Candrinho B, Martins MDR, da Cunha S, Pereira FM,

Samo Gudo E. Care-seeking behaviour and treatment practices for malaria in

children under 5 years in Mozambique: a secondary analysis of 2011 DHS and 2015

IMASIDA datasets. Malar J. 2019 Apr 2;18(1):115. doi: 10.1186/s12936-019-2751-9.

PMID: 30940127; PMCID: PMC6444821.

127: Pilmis B, Delory T, Groh M, Weiss E, Emirian A, Lecuyer H, Lesprit P, Zahar

JR. Extended-spectrum beta-lactamase-producing Enterobacteriaceae (ESBL-PE)

infections: are carbapenem alternatives achievable in daily practice? Int J

Infect Dis. 2015 Oct;39:62-7. doi: 10.1016/j.ijid.2015.08.011. Epub 2015 Sep 12.

PMID: 26327124.

128: Rystedt K, Hedin K, Tyrstrup M, Skoog-Ståhlgren G, Edlund C, Giske CG,

Gunnarsson R, Sundvall PD. Agreement between rapid antigen detection test and

culture for group A streptococcus in patients recently treated for

pharyngotonsillitis - a prospective observational study in primary care. Scand J

Prim Health Care. 2023 Mar;41(1):91-97. doi: 10.1080/02813432.2023.2182631. Epub

2023 Mar 7. PMID: 36880344; PMCID: PMC10088972.

129: Nakajima K, Akebo H, Tsugihashi Y, Ishimaru H, Sada R. Association of

physician experience with a higher prescription rate of anti-influenza agents in

low-risk patients. Intern Emerg Med. 2021 Aug;16(5):1215-1221. doi:

10.1007/s11739-020-02570-2. Epub 2021 Jan 2. PMID: 33389450.

130: López JJ, Cortázar Y, Acosta Á, Vargas-Peláez CM, Rossi F. Drug utilization

study of two generic antibiotics in a tertiary hospital in Bogotá. Biomedica.

2018 Sep 1;38(3):398-406. doi: 10.7705/biomedica.v38i4.3831. PMID: 30335245.

131: Bruggink SC, Eekhof JA, Egberts PF, van Blijswijk SC, Assendelft WJ,

Gussekloo J. Natural course of cutaneous warts among primary schoolchildren: a

prospective cohort study. Ann Fam Med. 2013 Sep-Oct;11(5):437-41. doi:

10.1370/afm.1508. PMID: 24019275; PMCID: PMC3767712.

132: Njei B, Esserman D, Krishnan S, Ohl M, Tate JP, Hauser RG, Taddei T, Lim J,

Justice AC. Regional and Rural-Urban Differences in the Use of Direct-acting

Antiviral Agents for Hepatitis C Virus: The Veteran Birth Cohort. Med Care. 2019

Apr;57(4):279-285. doi: 10.1097/MLR.0000000000001071. PMID: 30807449; PMCID:

PMC6436819.

133: Minotti C, Mengato D, De Pieri M, Trivellato S, Francavilla A, Di Chiara C,

Liberati C, Mattera R, Biffi A, Giaquinto C, Venturini F, Donà D. Early

Treatments of Fragile Children with COVID-19-Results of CLEVER (Children COVID

Early Treatment), a Retrospective, Observational Study. Viruses. 2023 Jan

10;15(1):192. doi: 10.3390/v15010192. PMID: 36680232; PMCID: PMC9867507.

134: Fishman E, Crawford G, DeVries A, Hackell J, Haynes K, Helm M, Wall E,

Agiro A. Association between early-childhood antibiotic exposure and subsequent

asthma in the US Medicaid population. Ann Allergy Asthma Immunol. 2019

Aug;123(2):186-192.e9. doi: 10.1016/j.anai.2019.05.018. Epub 2019 May 31. PMID:

31158472.

135: Dekker AR, Verheij TJ, van der Velden AW. Inappropriate antibiotic

prescription for respiratory tract indications: most prominent in adult

patients. Fam Pract. 2015 Aug;32(4):401-7. doi: 10.1093/fampra/cmv019. Epub 2015

Apr 24. PMID: 25911505.

136: Covino M, Buonsenso D, Gatto A, Morello R, Curatole A, Simeoni B,

Franceschi F, Chiaretti A. Determinants of antibiotic prescriptions in a large

cohort of children discharged from a pediatric emergency department. Eur J

Pediatr. 2022 May;181(5):2017-2030. doi: 10.1007/s00431-022-04386-y. Epub 2022

Feb 4. PMID: 35118518; PMCID: PMC8813572.

137: Fukuchi T, Iwata K, Ohji G. Failure of early diagnosis of infective

endocarditis in Japan--a retrospective descriptive analysis. Medicine

(Baltimore). 2014 Dec;93(27):e237. doi: 10.1097/MD.0000000000000237. PMID:

25501088; PMCID: PMC4602777.

138: Schot MJC, Dekker ARJ, van Werkhoven CH, van der Velden AW, Cals JWL,

Broekhuizen BDL, Hopstaken RM, de Wit NJ, Verheij TJM. Burden of disease in

children with respiratory tract infections in primary care: diary-based cohort

study. Fam Pract. 2019 Nov 18;36(6):723-729. doi: 10.1093/fampra/cmz024. PMID:

31166598; PMCID: PMC7006994.

139: Luo J, Gagne JJ, Landon J, Avorn J, Kesselheim AS. Comparative

effectiveness and safety of thalidomide and lenalidomide in patients with

multiple myeloma in the United States of America: A population-based cohort

study. Eur J Cancer. 2017 Jan;70:22-33. doi: 10.1016/j.ejca.2016.10.018. Epub

2016 Nov 17. PMID: 27866096.

140: Palin V, Mölter A, Belmonte M, Ashcroft DM, White A, Welfare W, van Staa T.

Antibiotic prescribing for common infections in UK general practice: variability

and drivers. J Antimicrob Chemother. 2019 Aug 1;74(8):2440-2450. doi:

10.1093/jac/dkz163. PMID: 31038162; PMCID: PMC6640319.

141: Dekker ARJ, Verheij TJM, van der Velden AW. Antibiotic management of

children with infectious diseases in Dutch Primary Care. Fam Pract. 2017 Apr

1;34(2):169-174. doi: 10.1093/fampra/cmw125. PMID: 28122841.

142: Zhao SR, Griffin MR, Patterson BL, Mace RL, Wyatt D, Zhu Y, Talbot HK. Risk

Factors for Outpatient Use of Antibiotics in Children with Acute Respiratory

Illnesses. South Med J. 2017 Mar;110(3):172-180. doi:

10.14423/SMJ.0000000000000622. PMID: 28257541; PMCID: PMC5338648.

143: Roggen I, van Berlaer G, Gordts F, Pierard D, Hubloue I. Acute sore throat

in children at the emergency department: best medical practice? Eur J Emerg Med.

2015 Oct;22(5):343-7. doi: 10.1097/MEJ.0000000000000175. PMID: 24999672.

144: Ruzicka DJ, Imai K, Takahashi K, Naito T. Comorbidities and the use of

comedications in people living with HIV on antiretroviral therapy in Japan: a

cross-sectional study using a hospital claims database. BMJ Open. 2018 Jun

14;8(6):e019985. doi: 10.1136/bmjopen-2017-019985. PMID: 29903786; PMCID:

PMC6009456.

145: La Vecchia A, Teklie BG, Mulu DA, Toitole KK, Montalbetti F, Agostoni C,

Hessebo TT, Tsegaye A, Pietravalle A, Manenti F, Tognon F, Pisani L, Hagos E.

Adherence to WHO guidelines on severe pneumonia management in children and its

impact on outcome: an observational study at Jinka General Hospital in Ethiopia.

Front Public Health. 2023 Jul 27;11:1189684. doi: 10.3389/fpubh.2023.1189684.

PMID: 37575120; PMCID: PMC10415009.

146: Rydland E, Høye S, Størdal K. Antibiotic use for airway infections in

Norwegian children-A national register-based study. Acta Paediatr. 2024

Mar;113(3):537-543. doi: 10.1111/apa.17052. Epub 2023 Nov 29. PMID: 38031498.

147: Alejandre C, Guitart C, Balaguer M, Torrús I, Bobillo-Perez S, Cambra FJ,

Jordan I. Use of procalcitonin and C-reactive protein in the diagnosis of

bacterial infection in infants with severe bronchiolitis. Eur J Pediatr. 2021

Mar;180(3):833-842. doi: 10.1007/s00431-020-03790-6. Epub 2020 Sep 14. PMID:

32929531.

148: Sigurdsson EL, Blondal AB, Jonsson JS, Tomasdottir MO, Hrafnkelsson H,

Linnet K, Sigurdsson JA. How primary healthcare in Iceland swiftly changed its

strategy in response to the COVID-19 pandemic. BMJ Open. 2020 Dec

7;10(12):e043151. doi: 10.1136/bmjopen-2020-043151. PMID: 33293329; PMCID:

PMC7722808.

149: Therkelsen LH, Skov ST, Laursen M, Lange J. Percutaneous needle fasciotomy

in Dupuytren contracture: a register-based, observational cohort study on

complications in 3,331 treated fingers in 2,257 patients. Acta Orthop. 2020

Jun;91(3):326-330. doi: 10.1080/17453674.2020.1726057. Epub 2020 Feb 14. PMID:

32056475; PMCID: PMC8023958.

150: Goupil B, Balusson F, Naudet F, Esvan M, Bastian B, Chapron A, Frouard P.

Association between gifts from pharmaceutical companies to French general

practitioners and their drug prescribing patterns in 2016: retrospective study

using the French Transparency in Healthcare and National Health Data System

databases. BMJ. 2019 Nov 5;367:l6015. doi: 10.1136/bmj.l6015. Erratum in: BMJ.

2019 Nov 21;367:l6613. doi: 10.1136/bmj.l6613. PMID: 31690553; PMCID:

PMC6830500.

151: de Jong J, Bos JH, de Vries TW, de Jong-van den Berg LT. Use of antibiotics

in rural and urban regions in The Netherlands: an observational drug utilization

study. BMC Public Health. 2014 Jul 3;14:677. doi: 10.1186/1471-2458-14-677.

PMID: 24992967; PMCID: PMC4090652.

152: Rebnord IK, Sandvik H, Mjelle AB, Hunskaar S. Out-of-hours antibiotic

prescription after screening with C reactive protein: a randomised controlled

study. BMJ Open. 2016 May 12;6(5):e011231. doi: 10.1136/bmjopen-2016-011231.

Erratum in: BMJ Open. 2016 Sep 09;6(9):e011231corr1. doi:

10.1136/bmjopen-2016-011231corr1. PMID: 27173814; PMCID: PMC4874126.

153: Dimopoulou D, Vourli S, Douros K, Pournaras S, Papaevangelou V. Use of

point-of-care molecular tests reduces hospitalization and oseltamivir

administration in children presenting with influenza-like illness. J Med Virol.

2021 Jun;93(6):3944-3948. doi: 10.1002/jmv.26538. Epub 2020 Oct 8. PMID:

32965697.

154: Baliatsas C, Dückers M, Smit L, Heederik D, Yzermans J. Morbidity Rates in

an Area with High Livestock Density: A Registry-Based Study Including Different

Groups of Patients with Respiratory Health Problems. Int J Environ Res Public

Health. 2020 Mar 1;17(5):1591. doi: 10.3390/ijerph17051591. PMID: 32121551;

PMCID: PMC7084699.

155: Hagedoorn NN, Borensztajn D, Nijman RG, Nieboer D, Herberg JA, Balode A,

von Both U, Carrol E, Eleftheriou I, Emonts M, van der Flier M, de Groot R,

Kohlmaier B, Lim E, Maconochie I, Martinón-Torres F, Pokorn M, Strle F, Tsolia

M, Zavadska D, Zenz W, Levin M, Vermont C, Moll HA. Development and validation

of a prediction model for invasive bacterial infections in febrile children at

European Emergency Departments: MOFICHE, a prospective observational study. Arch

Dis Child. 2021 Jul;106(7):641-647. doi: 10.1136/archdischild-2020-319794. Epub

2020 Nov 18. PMID: 33208397; PMCID: PMC8237171.

156: Sviestina I, Mozgis D. Observational Study of Antibiotic Usage at the

Children's Clinical University Hospital in Riga, Latvia. Medicina (Kaunas). 2018

Oct 23;54(5):74. doi: 10.3390/medicina54050074. PMID: 30360478; PMCID:

PMC6262611.

157: Schmitz T, Beynon F, Musard C, Kwiatkowski M, Landi M, Ishaya D, Zira J,

Muazu M, Renner C, Emmanuel E, Bulus SG, Rossi R. Effectiveness of an electronic

clinical decision support system in improving the management of childhood

illness in primary care in rural Nigeria: an observational study. BMJ Open. 2022

Jul 21;12(7):e055315. doi: 10.1136/bmjopen-2021-055315. PMID: 35863838; PMCID:

PMC9310162.

158: Chiappini E, Lisi C, Giacomet V, Erba P, Bernardi S, Zangari P, Di Biagio

A, Taramasso L, Giaquinto C, Rampon O, Gabiano C, Garazzino S, Tagliabue C,

Esposito S, Bruzzese E, Badolato R, Zanaboni D, Cellini M, Dedoni M, Mazza A,

Pession A, Giannini AM, Salvini F, Dodi I, Carloni I, Cazzato S, Tovo PA, de

Martino M, Galli L; Italian Register for HIV Infection in Children. Off-label

use of combined antiretroviral therapy, analysis of data collected by the

Italian Register for HIV-1 infection in paediatrics in a large cohort of

children. BMC Infect Dis. 2022 Jan 15;22(1):55. doi: 10.1186/s12879-022-07026-w.

PMID: 35033018; PMCID: PMC8760752.

159: Shekhawat NS, Shtein RM, Blachley TS, Stein JD. Antibiotic Prescription

Fills for Acute Conjunctivitis among Enrollees in a Large United States Managed

Care Network. Ophthalmology. 2017 Aug;124(8):1099-1107. doi:

10.1016/j.ophtha.2017.04.034. Epub 2017 Jun 16. PMID: 28624168; PMCID:

PMC9482449.

160: Nwankwo L, Butt Z, Schelenz S. Experience of Ceftazidime/avibactam in a UK

tertiary cardiopulmonary specialist center. Expert Rev Anti Infect Ther. 2021

Jan;19(1):101-108. doi: 10.1080/14787210.2020.1810568. Epub 2020 Sep 2. PMID:

32799594.

161: Conterno LO, Trabasso P, Resende MR, Paschoali PS, Pascucci LB, Moretti ML.

Six-year experience with GM test in hematological patients in a public Brazilian

tertiary hospital. Braz J Infect Dis. 2024 Jan-Feb;28(1):103718. doi:

10.1016/j.bjid.2024.103718. Epub 2024 Feb 6. PMID: 38336355; PMCID: PMC10901827.

162: Garber C, Plotnikova N, Au SC, Sorensen EP, Gottlieb A. Biologic and

Conventional Systemic Therapies Show Similar Safety and Efficacy in Elderly and

Adult Patients With Moderate to Severe Psoriasis. J Drugs Dermatol. 2015

Aug;14(8):846-52. PMID: 26267729.

163: El Halabi J, Palmer N, McDuffie M, Golub JJ, Fox K, Kohane I, Farhat MR.

Measuring health-care delays among privately insured patients with tuberculosis

in the USA: an observational cohort study. Lancet Infect Dis. 2021

Aug;21(8):1175-1183. doi: 10.1016/S1473-3099(20)30732-5. Epub 2021 Mar 23. PMID:

33770534.

164: Senn N, Rarau P, Salib M, Manong D, Siba P, Rogerson S, Mueller I, Genton

B. Use of antibiotics within the IMCI guidelines in outpatient settings in Papua

New Guinean children: an observational and effectiveness study. PLoS One. 2014

Mar 13;9(3):e90990. doi: 10.1371/journal.pone.0090990. PMID: 24626194; PMCID:

PMC3953204.

165: Özger HS, Fakıoğlu DM, Erbay K, Albayrak A, Hızel K. Inapropriate use of

antibiotics effective against gram positive microorganisms despite restrictive

antibiotic policies in ICUs: a prospective observational study. BMC Infect Dis.

2020 Apr 19;20(1):289. doi: 10.1186/s12879-020-05005-7. PMID: 32306946; PMCID:

PMC7169036.

166: Glanzmann C, Frey B, Meier CR, Vonbach P. Analysis of medication

prescribing errors in critically ill children. Eur J Pediatr. 2015

Oct;174(10):1347-55. doi: 10.1007/s00431-015-2542-4. Epub 2015 Apr 22. PMID:

25899070.

167: Risgaard B, Winkel BG, Jabbari R, Lynge TH, Wissenberg M, Glinge C, Haunsø

S, Behr ER, Fink-Jensen A, Gislason GH, Tfelt-Hansen J. Sudden Cardiac Death:

Pharmacotherapy and Proarrhythmic Drugs: A Nationwide Cohort Study in Denmark.

JACC Clin Electrophysiol. 2017 May;3(5):473-481. doi:

10.1016/j.jacep.2016.12.023. Epub 2017 Mar 29. PMID: 29759603.

168: Camporesi A, Yock-Corrales A, Gomez-Vargas J, Roland D, Gonzalez M,

Barreiro S, Morello R, Brizuela M, Buonsenso D. Management and outcomes of

bronchiolitis in Italy and Latin America: a multi-center, prospective,

observational study. Eur J Pediatr. 2024 Jun;183(6):2733-2742. doi:

10.1007/s00431-024-05530-6. Epub 2024 Mar 30. PMID: 38554172; PMCID:

PMC11098874.

169: Lawes T, Lopez-Lozano JM, Nebot CA, Macartney G, Subbarao-Sharma R, Wares

KD, Sinclair C, Gould IM. Effect of a national 4C antibiotic stewardship

intervention on the clinical and molecular epidemiology of Clostridium difficile

infections in a region of Scotland: a non-linear time-series analysis. Lancet

Infect Dis. 2017 Feb;17(2):194-206. doi: 10.1016/S1473-3099(16)30397-8. Epub

2016 Nov 4. PMID: 27825595.

170: Abramavicius S, Stundziene A, Jankauskaite L, Vitkauskiene A, Kowalski IM,

Wojtkiewicz J, Stankevicius E. Novel approach towards antimicrobial chemotherapy

optimization in lower respiratory tract infections in children: An observational

study. Medicine (Baltimore). 2021 Oct 1;100(39):e26585. doi:

10.1097/MD.0000000000026585. PMID: 34596107; PMCID: PMC8483859.

171: Bimba HV, Roy V, Batta A, Daga MK. Drug utilization, rationality, and cost

analysis of antimicrobial medicines in a tertiary care teaching hospital of

Northern India: A prospective, observational study. Indian J Pharmacol. 2020

May-Jun;52(3):179-188. doi: 10.4103/ijp.IJP_225_19. Epub 2020 Aug 4. PMID:

32874000; PMCID: PMC7446674.

172: Tefera GM, Feyisa BB, Kebede TM. Antimicrobial use-related problems and

their costs in surgery ward of Jimma University Medical Center: Prospective

observational study. PLoS One. 2019 May 17;14(5):e0216770. doi:

10.1371/journal.pone.0216770. PMID: 31100088; PMCID: PMC6524801.

173: Kornfält Isberg H, Melander E, Hedin K, Mölstad S, Beckman A. Uncomplicated

urinary tract infections in Swedish primary care; etiology, resistance and

treatment. BMC Infect Dis. 2019 Feb 13;19(1):155. doi:

10.1186/s12879-019-3785-x. PMID: 30760219; PMCID: PMC6375206.

174: Pedersen CJ, Rogan DT, Yang S, Quinn JV. Using a novel rapid viral test to

improve triage of emergency department patients with acute respiratory illness

during flu season. J Clin Virol. 2018 Nov;108:72-76. doi:

10.1016/j.jcv.2018.09.008. Epub 2018 Sep 15. PMID: 30261422; PMCID: PMC7106347.

175: Leopoldino RD, Santos MT, Costa TX, Martins RR, Oliveira AG. Drug related

problems in the neonatal intensive care unit: incidence, characterization and

clinical relevance. BMC Pediatr. 2019 Apr 26;19(1):134. doi:

10.1186/s12887-019-1499-2. PMID: 31027487; PMCID: PMC6485091.

176: Bhattacharya D, Belperio PS, Shahoumian TA, Loomis TP, Goetz MB, Mole LA,

Backus LI. Effectiveness of All-Oral Antiviral Regimens in 996 Human

Immunodeficiency Virus/Hepatitis C Virus Genotype 1-Coinfected Patients Treated

in Routine Practice. Clin Infect Dis. 2017 Jun 15;64(12):1711-1720. doi:

10.1093/cid/cix111. PMID: 28199525.

177: Bollestad M, Grude N, Solhaug S, Raffelsberger N, Handal N, Nilsen HS,

Romstad MR, Emmert A, Tveten Y, Søraas A, Jenum PA, Jenum S, Møller-Stray J,

Weme ET, Lindbaek M, Simonsen GS; (the Norwegian ESBL UTI study group). Clinical

and bacteriological efficacy of pivmecillinam treatment for uncomplicated

urinary tract infections caused by ESBL-producing Escherichia coli: a

prospective, multicentre, observational cohort study. J Antimicrob Chemother.

2018 Sep 1;73(9):2503-2509. doi: 10.1093/jac/dky230. PMID: 29982514.

178: Kornelius E, Chiou JY, Yang YS, Lo SC, Peng CH, Lai YR, Huang CN. Iodinated

Contrast Media-Induced Thyroid Dysfunction in Euthyroid Nodular Goiter Patients.

Thyroid. 2016 Aug;26(8):1030-8. doi: 10.1089/thy.2016.0051. PMID: 27315873.

179: Hagedoorn NN, Wagenaar JHL, Nieboer D, Bath D, Von Both U, Carrol ED,

Eleftheriou I, Emonts M, Van Der Flier M, De Groot R, Herberg J, Kohlmaier B,

Levin M, Lim E, Maconochie I, Martinon-Torres F, Nijman R, Pokorn M, Rivero

Calle I, Tsolia M, Yeung S, Zavadska D, Zenz W, Vermont CL, Oostenbrink R, Moll

HA; PERFORM consortium. Impact of a clinical decision rule on antibiotic

prescription for children with suspected lower respiratory tract infections

presenting to European emergency departments: a simulation study based on

routine data. J Antimicrob Chemother. 2021 Apr 13;76(5):1349-1357. doi:

10.1093/jac/dkab023. PMID: 33564871.

180: Rolle CP, Nguyen V, Patel K, Cruz D, DeJesus E, Hinestrosa F. Real-world

efficacy and safety of switching to bictegravir/emtricitabine/tenofovir

alafenamide in older people living with HIV. Medicine (Baltimore). 2021 Sep

24;100(38):e27330. doi: 10.1097/MD.0000000000027330. PMID: 34559154; PMCID:

PMC8462546.

181: Williamson E, Denaxas S, Morris S, Clarke CS, Thomas M, Evans H, Direk K,

Gonzalez-Izquierdo A, Little P, Lund V, Blackshaw H, Schilder A, Philpott C,

Hopkins C, Carpenter J, Programme Team OBOTM. Risk of mortality and

cardiovascular events following macrolide prescription in chronic rhinosinusitis

patients: a cohort study using linked primary care electronic health records.

Rhinology. 2019 Aug 1;57(4):252-260. doi: 10.4193/Rhin18.237. PMID: 30928998.

182: Joseph Davey DL, Nyemba DC, Mvududu R, Mashele N, Johnson L, Bekker LG,

Dean SS, Bheemraj K, Coates TJ, Myer L. Pregnancy outcomes following self-

reported and objective-measured exposure to oral preexposure prophylaxis in

South Africa. AIDS. 2024 Jan 1;38(1):75-83. doi: 10.1097/QAD.0000000000003729.

Epub 2023 Nov 22. PMID: 37720980; PMCID: PMC10715696.

183: Valeur KS, Hertel SA, Lundstrøm KE, Holst H. The Cumulative Daily Tolerance

Levels of Potentially Toxic Excipients Ethanol and Propylene Glycol Are Commonly

Exceeded in Neonates and Infants. Basic Clin Pharmacol Toxicol. 2018

May;122(5):523-530. doi: 10.1111/bcpt.12950. Epub 2018 Jan 17. PMID: 29281181.

184: Kamikawa J, Granato CF, Bellei N. Viral aetiology of common colds of

outpatient children at primary care level and the use of antibiotics. Mem Inst

Oswaldo Cruz. 2015 Nov;110(7):884-9. doi: 10.1590/0074-02760150154. PMID:

26560978; PMCID: PMC4660617.

185: Depew RE, Gonzales G. Differences in US antibiotic prescription use by

facility and patient characteristics: evidence from the National Ambulatory

Medical Care Survey. Fam Pract. 2020 Mar 25;37(2):180-186. doi:

10.1093/fampra/cmz049. PMID: 31536616.

186: Al-Hadithi D, Al-Zakwani I, Balkhair A, Al Suleimani YM. Evaluation of the

appropriateness of meropenem prescribing at a tertiary care hospital: A

retrospective study in Oman. Int J Infect Dis. 2020 Jul;96:180-186. doi:

10.1016/j.ijid.2020.04.045. Epub 2020 Apr 24. PMID: 32339716.

187: Teratani Y, Hagiya H, Koyama T, Adachi M, Ohshima A, Zamami Y, Tanaka HY,

Tatebe Y, Tasaka K, Mikami N, Shinomiya K, Kitamura Y, Kano MR, Hinotsu S, Sendo

T. Pattern of antibiotic prescriptions for outpatients with acute respiratory

tract infections in Japan, 2013-15: a retrospective observational study. Fam

Pract. 2019 Jul 31;36(4):402-409. doi: 10.1093/fampra/cmy094. PMID: 30272148.

188: Teratani Y, Hagiya H, Koyama T, Ohshima A, Zamami Y, Tatebe Y, Tasaka K,

Shinomiya K, Kitamura Y, Sendo T, Hinotsu S, Kano MR. Association between rapid

antigen detection tests and antibiotics for acute pharyngitis in Japan: A

retrospective observational study. J Infect Chemother. 2019 Apr;25(4):267-272.

doi: 10.1016/j.jiac.2018.12.005. Epub 2019 Jan 12. PMID: 30642770.

189: Hassen-Khodja C, Gras G, Grammatico-Guillon L, Dupuy C, Gomez JF, Freslon

L, Dailloux JF, Soufflet A, Bernard L. Hospital and ambulatory management, and

compliance to treatment in HIV infection: regional health insurance agency

analysis. Med Mal Infect. 2014 Sep;44(9):423-8. doi:

10.1016/j.medmal.2014.08.004. Epub 2014 Sep 16. PMID: 25234379.

190: Härtel C, Hartz A, Bahr L, Gille C, Gortner L, Simon A, Orlikowsky T,

Müller A, Körner T, Henneke P, Haase R, Zemlin M, Viemann D, Gebauer C, Thome U,

Ziegler A, Rupp J, Herting E, Göpel W; German Neonatal Network. Media Stories on

NICU Outbreaks Lead to an Increased Prescription Rate of Third-Line Antibiotics

in the Community of Neonatal Care. Infect Control Hosp Epidemiol. 2016

Aug;37(8):924-930. doi: 10.1017/ice.2016.95. Epub 2016 May 4. PMID: 27143176.

191: Amadieu R, Brehin C, Chahine A, Grouteau E, Dubois D, Munzer C, Flumian C,

Brissaud O, Ros B, Jean G, Brotelande C, Travert B, Savy N, Boeuf B, Ghostine G,

Popov I, Duport P, Wolff R, Maurice L, Dauger S, Breinig S. Compliance with

antibiotic therapy guidelines in french paediatric intensive care units: a

multicentre observational study. BMC Infect Dis. 2024 Jun 12;24(1):582. doi:

10.1186/s12879-024-09472-0. PMID: 38867164; PMCID: PMC11170905.

192: Hernandez-Santiago V, Davey PG, Nathwani D, Marwick CA, Guthrie B. Changes

in resistance among coliform bacteraemia associated with a primary care

antimicrobial stewardship intervention: A population-based interrupted time

series study. PLoS Med. 2019 Jun 7;16(6):e1002825. doi:

10.1371/journal.pmed.1002825. PMID: 31173597; PMCID: PMC6555503.

193: Usonis V, Jackowska T, Petraitiene S, Sapala A, Neculau A, Stryjewska I,

Devadiga R, Tafalla M, Holl K. Incidence of acute otitis media in children below

6 years of age seen in medical practices in five East European countries. BMC

Pediatr. 2016 Jul 26;16:108. doi: 10.1186/s12887-016-0638-2. PMID: 27457584;

PMCID: PMC4960887.

194: Fukuda A, Otake S, Kimura M, Natsuki A, Ishida A, Kasai M. Trend of oral

antimicrobial use after removal of broad-spectrum antimicrobials from the

formulary at a pediatric primary emergency medical center. J Infect Chemother.

2023 May;29(5):502-507. doi: 10.1016/j.jiac.2023.01.002. Epub 2023 Jan 5. PMID:

36621765.

195: Mula CT, Middleton L, Human N, Varga C. Assessment of factors that

influence timely administration of initial antibiotic dose using collaborative

process mapping at a referral hospital in Malawi: a case study of pneumonia

patients. BMC Infect Dis. 2018 Dec 27;18(1):697. doi: 10.1186/s12879-018-3620-9.

PMID: 30587155; PMCID: PMC6307292.

196: Yoon YK, Park GC, An H, Chun BC, Sohn JW, Kim MJ. Trends of Antibiotic

Consumption in Korea According to National Reimbursement Data (2008-2012): A

Population-Based Epidemiologic Study. Medicine (Baltimore). 2015

Nov;94(46):e2100. doi: 10.1097/MD.0000000000002100. PMID: 26579825; PMCID:

PMC4652834.

197: de Bont EG, Lepot JM, Hendrix DA, Loonen N, Guldemond-Hecker Y, Dinant GJ,

Cals JW. Workload and management of childhood fever at general practice out-of-

hours care: an observational cohort study. BMJ Open. 2015 May 19;5(5):e007365.

doi: 10.1136/bmjopen-2014-007365. PMID: 25991452; PMCID: PMC4442146.

198: Zablotska IB, Selvey C, Guy R, Price K, Holden J, Schmidt HM, McNulty A,

Smith D, Jin F, Amin J, Cooper DA, Grulich AE; EPIC-NSW study group. Expanded

HIV pre-exposure prophylaxis (PrEP) implementation in communities in New South

Wales, Australia (EPIC-NSW): design of an open label, single arm implementation

trial. BMC Public Health. 2018 Feb 2;18(1):210. doi: 10.1186/s12889-017-5018-9.

Erratum in: BMC Public Health. 2018 Feb 28;18(1):297. doi:

10.1186/s12889-018-5173-7. PMID: 29394918; PMCID: PMC5797394.

199: Miyawaki A, Kitajima K, Iwata A, Sato D, Tsugawa Y. Physician

characteristics associated with antiviral prescriptions for older adults with

COVID-19 in Japan: an observational study. BMJ Open. 2024 Mar 15;14(3):e083342.

doi: 10.1136/bmjopen-2023-083342. PMID: 38490659; PMCID: PMC10946352.

200: Gordon SC, Muir AJ, Lim JK, Pearlman B, Argo CK, Ramani A, Maliakkal B,

Alam I, Stewart TG, Vainorius M, Peter J, Nelson DR, Fried MW, Reddy KR; HCV-

TARGET study group. Safety profile of boceprevir and telaprevir in chronic

hepatitis C: real world experience from HCV-TARGET. J Hepatol. 2015

Feb;62(2):286-93. doi: 10.1016/j.jhep.2014.08.052. Epub 2014 Sep 10. PMID:

25218788; PMCID: PMC4586075.

201: Jain AK, Naimi S, Jain S. Assessment of Antimicrobial Prescribing Pattern

in the Outpatient Department of Ophthalmology in a Tertiary Care Hospital of

Western Uttar Pradesh, India. Nepal J Ophthalmol. 2018 Jul;10(20):130-138. doi:

10.3126/nepjoph.v10i2.23014. PMID: 31056555.

202: Ciccone EJ, Kabugho L, Baguma E, Muhindo R, Juliano JJ, Mulogo E, Boyce RM.

Rapid Diagnostic Tests to Guide Case Management of and Improve Antibiotic

Stewardship for Pediatric Acute Respiratory Illnesses in Resource-Constrained

Settings: a Prospective Cohort Study in Southwestern Uganda. Microbiol Spectr.

2021 Dec 22;9(3):e0169421. doi: 10.1128/Spectrum.01694-21. Epub 2021 Nov 24.

Erratum in: Microbiol Spectr. 2022 Feb 23;10(1):e0044322. doi:

10.1128/spectrum.00443-22. PMID: 34817224; PMCID: PMC8612158.

203: Kelesidis T, Braykov N, Uslan DZ, Morgan DJ, Gandra S, Johannsson B,

Schweizer ML, Weisenberg SA, Young H, Cantey J, Perencevich E, Septimus E,

Srinivasan A, Laxminarayan R. Indications and Types of Antibiotic Agents Used in

6 Acute Care Hospitals, 2009-2010: A Pragmatic Retrospective Observational

Study. Infect Control Hosp Epidemiol. 2016 Jan;37(1):70-9. doi:

10.1017/ice.2015.226. Epub 2015 Oct 12. PMID: 26456803; PMCID: PMC7011437.

204: Saqib A, Sarwar MR, Sarfraz M, Iftikhar S. Causality and preventability

assessment of adverse drug events of antibiotics among inpatients having

different lengths of hospital stay: a multicenter, cross-sectional study in

Lahore, Pakistan. BMC Pharmacol Toxicol. 2018 Jun 25;19(1):34. doi:

10.1186/s40360-018-0222-5. PMID: 29941052; PMCID: PMC6019808.

205: Lee YC, Huang YJ, Hung MC, Hung SC, Hsiao CY, Cho HL, Lai LF, Tong SH, Wang

JT. Risk factors associated with the development of seizures among adult

patients treated with ertapenem: A matched case-control study. PLoS One. 2017

Jul 31;12(7):e0182046. doi: 10.1371/journal.pone.0182046. PMID: 28759588; PMCID:

PMC5536326.

206: Sharma M, Damlin A, Pathak A, Stålsby Lundborg C. Antibiotic Prescribing

among Pediatric Inpatients with Potential Infections in Two Private Sector

Hospitals in Central India. PLoS One. 2015 Nov 5;10(11):e0142317. doi:

10.1371/journal.pone.0142317. PMID: 26540104; PMCID: PMC4634959.

207: Hagedoorn NN, Borensztajn DM, Nijman R, Balode A, von Both U, Carrol ED,

Eleftheriou I, Emonts M, van der Flier M, de Groot R, Herberg J, Kohlmaier B,

Lim E, Maconochie I, Martinon-Torres F, Nieboer D, Pokorn M, Strle F, Tsolia M,

Yeung S, Zavadska D, Zenz W, Vermont C, Levin M, Moll HA; PERFORM consortium.

Variation in antibiotic prescription rates in febrile children presenting to

emergency departments across Europe (MOFICHE): A multicentre observational

study. PLoS Med. 2020 Aug 19;17(8):e1003208. doi: 10.1371/journal.pmed.1003208.

PMID: 32813708; PMCID: PMC7444592.

208: Quirke M, Saunders J, O'Sullivan R, Wakai A. The management of cellulitis

in emergency departments: antibiotic-prescribing practices and adherence to

practice guidelines in Ireland. Eur J Emerg Med. 2016 Jun;23(3):173-8. doi:

10.1097/MEJ.0000000000000245. PMID: 25647040.

209: Mor A, Antonsen S, Kahlert J, Holsteen V, Jørgensen S, Holm-Pedersen J,

Sørensen HT, Pedersen O, Ehrenstein V. Prenatal exposure to systemic

antibacterials and overweight and obesity in Danish schoolchildren: a prevalence

study. Int J Obes (Lond). 2015 Oct;39(10):1450-5. doi: 10.1038/ijo.2015.129.

Epub 2015 Jul 16. PMID: 26178293.

210: Ono A, Aoyagi K, Muraki Y, Asai Y, Tsuzuki S, Koizumi R, Azuma T, Kusama Y,

Ohmagari N. Trends in healthcare visits and antimicrobial prescriptions for

acute infectious diarrhea in individuals aged 65 years or younger in Japan from

2013 to 2018 based on administrative claims database: a retrospective

observational study. BMC Infect Dis. 2021 Sep 21;21(1):983. doi:

10.1186/s12879-021-06688-2. PMID: 34548008; PMCID: PMC8454076.

211: Baidya S, Hazra A, Datta S, Das AK. A study of antimicrobial use in

children admitted to pediatric medicine ward of a tertiary care hospital. Indian

J Pharmacol. 2017 Jan-Feb;49(1):10-15. doi: 10.4103/0253-7613.201034. PMID:

28458416; PMCID: PMC5351220.

212: Boussetta A, Kharbach N, Abdellatif A, Karray A, Jellouli M, Gargah T.

Predictive factors of urinary tract infections caused by extended-spectrum

β-lactamase-producing Escherichia coli in children: a prospective Tunisian

study. Tunis Med. 2023 Feb 11;101(2):285-291. English. PMID: 37682274; PMCID:

PMC11138556.

213: Bloechliger M, Schlagenhauf P, Toovey S, Schnetzler G, Tatt I, Tomianovic

D, Jick SS, Meier CR. Malaria chemoprophylaxis regimens: a descriptive drug

utilization study. Travel Med Infect Dis. 2014 Nov-Dec;12(6 Pt B):718-25. doi:

10.1016/j.tmaid.2014.05.006. Epub 2014 Jun 2. PMID: 24934849.

214: Hashash JG, Chintamaneni P, Ramos Rivers CM, Koutroubakis IE, Regueiro MD,

Baidoo L, Swoger JM, Barrie A, Schwartz M, Dunn MA, Binion DG. Patterns of

Antibiotic Exposure and Clinical Disease Activity in Inflammatory Bowel Disease:

A 4-year Prospective Study. Inflamm Bowel Dis. 2015 Nov;21(11):2576-82. doi:

10.1097/MIB.0000000000000534. PMID: 26296061.

215: Fazylov VC, Sitnikov IG, Malyshev NA, Silina EV, Shevshenko SB, Eganyan GA,

Korsantiya BM, Groppa LG. The Effect of Antiviral Therapy on the Incidence of

Bacterial Aggravations and Administration of Systemic Antibiotics in Patients

with Acute Respiratory Viral Infections and Influenza (Results of International

Cohort Observational Study). Antibiot Khimioter. 2016;61(11-12):39-47. English,

Russian. PMID: 29558059.

216: Lin HC, Lin HL, Wang LH, Hsu CY, Hsueh YM. Outcome of nonadherence to

antimicrobial treatment guidelines in ambulatory patients with acute cystitis: a

nationwide population-based study. Intern Med. 2014;53(17):1933-9. doi:

10.2169/internalmedicine.53.1308. Epub 2014 Sep 1. PMID: 25175125.

217: Demoré B, Le Govic D, Thilly N, Boivin JM, Pulcini C. Reliability of self-

reported recent antibiotic use among the general population: a cross-sectional

study. Clin Microbiol Infect. 2017 Jul;23(7):486.e7-486.e12. doi:

10.1016/j.cmi.2017.01.006. Epub 2017 Jan 19. PMID: 28110051.

218: Mazuel M, Moulier V, Bourrel AS, Guillier C, Tazi A, Jarreau PH, Chollat C.

Systematic culture of central catheters and infections related to catheters in a

neonatal intensive care unit: an observational study. Sci Rep. 2024 Apr

15;14(1):8647. doi: 10.1038/s41598-024-59371-2. PMID: 38622221; PMCID:

PMC11018835.

219: Agiro A, Gautam S, Wall E, Hackell J, Helm M, Barron J, Zaoutis T, Fleming-

Dutra KE, Hicks LA, Rosenberg A. Variation in Outpatient Antibiotic Dispensing

for Respiratory Infections in Children by Clinician Specialty and Treatment

Setting. Pediatr Infect Dis J. 2018 Dec;37(12):1248-1254. doi:

10.1097/INF.0000000000002004. PMID: 30408006.

220: Copp HL, Hanley J, Saigal CS, Saperston K; NIDDK Urologic Diseases in

America Project. Acute health care utilization and outcomes for outpatient-

treated urinary tract infections in children. J Pediatr Urol. 2016

Aug;12(4):234.e1-5. doi: 10.1016/j.jpurol.2016.05.016. Epub 2016 May 31. PMID:

27312876.

221: Schuijt TJ, Boss DS, Musson REA, Demir AY. Influence of point-of-care

C-reactive protein testing on antibiotic prescription habits in primary care in

the Netherlands. Fam Pract. 2018 Mar 27;35(2):179-185. doi:

10.1093/fampra/cmx081. PMID: 28973636.

222: Bhadury A , Roy UK , Ghosh T , Barman D , Mandal P . Assessment of

Prescribing Pattern and Safety Profile of Drugs Used in Intranasal Route in

Paediatric Age Group of Patients in a Tertiary Care Hospital. Kathmandu Univ Med

J (KUMJ). 2021 Jan.-Mar;19(73):62-68. PMID: 34812160.

223: Thaulow CM, Blix HS, Eriksen BH, Ask I, Myklebust TÅ, Berild D. Using a

period incidence survey to compare antibiotic use in children between a

university hospital and a district hospital in a country with low antimicrobial

resistance: a prospective observational study. BMJ Open. 2019 May

27;9(5):e027836. doi: 10.1136/bmjopen-2018-027836. PMID: 31138583; PMCID:

PMC6549646.

224: Launay E, Levieux K, Levy C, Dubos F, Martinot A, Vrignaud B, Lepage F,

Cohen R, Grimprel E, Hanf M, Angoulvant F, Gras-Le Guen C; GPIP. Compliance with

the current recommendations for prescribing antibiotics for paediatric

community-acquired pneumonia is improving: data from a prospective study in a

French network. BMC Pediatr. 2016 Aug 12;16(1):126. doi:

10.1186/s12887-016-0661-3. PMID: 27520057; PMCID: PMC4983061.

225: Shankar-Hari M, Donnelly A, Pinto R, Salih Z, McKenzie C, Terblanche M,

Adhikari NK. The influence of statin exposure on inflammatory markers in

patients with early bacterial infection: pilot prospective cohort study. BMC

Anesthesiol. 2014 Nov 19;14:106. doi: 10.1186/1471-2253-14-106. PMID: 25484622;

PMCID: PMC4256798.

226: Aamir M, Khan JA, Shakeel F, Shareef R, Shah N. Drug utilization in

neonatal setting of Pakistan: focus on unlicensed and off label drug

prescribing. BMC Pediatr. 2018 Jul 25;18(1):242. doi: 10.1186/s12887-018-1211-y.

PMID: 30045715; PMCID: PMC6060516.

227: Thompson CN, Phan MV, Hoang NV, Minh PV, Vinh NT, Thuy CT, Nga TT, Rabaa

MA, Duy PT, Dung TT, Phat VV, Nga TV, Tu le TP, Tuyen HT, Yoshihara K, Jenkins

C, Duong VT, Phuc HL, Tuyet PT, Ngoc NM, Vinh H, Chinh NT, Thuong TC, Tuan HM,

Hien TT, Campbell JI, Chau NV, Thwaites G, Baker S. A prospective multi-center

observational study of children hospitalized with diarrhea in Ho Chi Minh City,

Vietnam. Am J Trop Med Hyg. 2015 May;92(5):1045-52. doi: 10.4269/ajtmh.14-0655.

Epub 2015 Mar 23. PMID: 25802437; PMCID: PMC4426562.

228: Ansari AS, de Lusignan S, Hinton W, Munro N, McGovern A. The association

between diabetes, level of glycaemic control and eye infection: Cohort database

study. Prim Care Diabetes. 2017 Oct;11(5):421-429. doi:

10.1016/j.pcd.2017.05.009. Epub 2017 Jun 23. PMID: 28648963.

229: Di Mario S, Gagliotti C, Buttazzi R, Cisbani L, Di Girolamo C, Brambilla A,

Moro ML; regional working group “Progetto ProBA-Progetto Bambini e

Antibiotici-2014”. Observational pre-post study showed that a quality

improvement project reduced paediatric antibiotic prescribing rates in primary

care. Acta Paediatr. 2018 Oct;107(10):1805-1809. doi: 10.1111/apa.14381. Epub

2018 May 25. PMID: 29723913.

230: Holm A, Siersma V, Bjerrum L, Cordoba G. Availability of point-of-care

culture and microscopy in general practice - does it lead to more appropriate

use of antibiotics in patients with suspected urinary tract infection? Eur J Gen

Pract. 2020 Dec;26(1):175-181. doi: 10.1080/13814788.2020.1853697. PMID:

33356665; PMCID: PMC7781897.

231: Aas CF, Vold JH, Skurtveit S, Odsbu I, Chalabianloo F, Lim AG, Johansson

KA, Fadnes LT. Uptake and predictors of direct-acting antiviral treatment for

hepatitis C among people receiving opioid agonist therapy in Sweden and Norway:

a drug utilization study from 2014 to 2017. Subst Abuse Treat Prev Policy. 2020

Jun 30;15(1):44. doi: 10.1186/s13011-020-00286-2. PMID: 32605625; PMCID:

PMC7325258.

232: Lejone TI, Ringera I, Cheleboi M, Wagner S, Muhairwe J, Klimkait T,

Labhardt ND. The Treatment Cascade in Children With Unsuppressed Viral Load-A

Reality Check in Rural Lesotho, Southern Africa. J Acquir Immune Defic Syndr.

2018 Mar 1;77(3):250-256. doi: 10.1097/QAI.0000000000001597. PMID: 29189416.

233: Montecatine-Alonso E, Gil-Navarro MV, Fernández-Llamazares CM, Fernández-

Polo A, Soler-Palacín P, Llorente-Gutiérrez J, Gómez-Travecedo Calvo MT,

Esquivel-Mora MD, Pérez-Rodrigo I, Cisneros JM, Goycochea-Valdivia WA, Neth O;

Paediatric Antimicrobial Defined Daily Dose Study Group (KiDDDs). Antimicrobial

defined daily dose adjusted by weight: a proposal for antibiotic consumption

measurement in children. Enferm Infecc Microbiol Clin (Engl Ed). 2019

May;37(5):301-306. English, Spanish. doi: 10.1016/j.eimc.2018.07.011. Epub 2018

Sep 27. PMID: 30268590.

234: Chalmers JD, Akram AR, Singanayagam A, Wilcox MH, Hill AT. Risk factors for

Clostridium difficile infection in hospitalized patients with community-acquired

pneumonia. J Infect. 2016 Jul;73(1):45-53. doi: 10.1016/j.jinf.2016.04.008. Epub

2016 Apr 19. PMID: 27105657.

235: Zhao YJ, Wen JQ, Cheng K, Ming YZ, She XG, Liu H, Liu L, Ye QF, Ding BN.

Late, severe, noninfectious diarrhea after renal transplantation: high-risk

factors, therapy, and prognosis. Transplant Proc. 2013 Jul-Aug;45(6):2226-32.

doi: 10.1016/j.transproceed.2013.02.131. PMID: 23953533.

236: Hubert D, Dehillotte C, Munck A, David V, Baek J, Mely L, Dominique S,

Ramel S, Danner Boucher I, Lefeuvre S, Reynaud Q, Colomb-Jung V, Bakouboula P,

Lemonnier L. Retrospective observational study of French patients with cystic

fibrosis and a Gly551Asp-CFTR mutation after 1 and 2years of treatment with

ivacaftor in a real-world setting. J Cyst Fibros. 2018 Jan;17(1):89-95. doi:

10.1016/j.jcf.2017.07.001. Epub 2017 Jul 12. PMID: 28711222.

237: Adomi M, Iwagami M, Kawahara T, Hamada S, Iijima K, Yoshie S, Ishizaki T,

Tamiya N. Factors associated with long-term urinary catheterisation and its

impact on urinary tract infection among older people in the community: a

population-based observational study in a city in Japan. BMJ Open. 2019 Jun

19;9(6):e028371. doi: 10.1136/bmjopen-2018-028371. PMID: 31221889; PMCID:

PMC6589038.

238: European Pregnancy and Paediatric HIV Cohort Collaboration (EPPICC) Study

Group in EuroCoord. Time to Switch to Second-line Antiretroviral Therapy in

Children With Human Immunodeficiency Virus in Europe and Thailand. Clin Infect

Dis. 2018 Feb 1;66(4):594-603. doi: 10.1093/cid/cix854. PMID: 29029056; PMCID:

PMC5796645.

239: Shayo GA, Moshiro C, Aboud S, Bakari M, Mugusi FM. Acceptability and

adherence to Isoniazid preventive therapy in HIV-infected patients clinically

screened for latent tuberculosis in Dar es Salaam, Tanzania. BMC Infect Dis.

2015 Aug 26;15:368. doi: 10.1186/s12879-015-1085-7. PMID: 26306511; PMCID:

PMC4549887.

240: Spradling PR, Xing J, Rupp LB, Moorman AC, Gordon SC, Lu M, Teshale EH,

Boscarino JA, Schmidt MA, Daida YG, Holmberg SD; Chronic Hepatitis Cohort Study

(CHeCS) Investigators. Low Uptake of Direct-acting Antiviral Therapy Among

Hepatitis C Patients With Advanced Liver Disease and Access to Care, 2014-2017.

J Clin Gastroenterol. 2021 Jan;55(1):77-83. doi: 10.1097/MCG.0000000000001344.

PMID: 32250999; PMCID: PMC10711731.

241: Liu CH, Wang JL, Su CP, Chuang JH, Chang CH, Lai MS. Oseltamivir use and

outcomes during the 2009 influenza A H1N1 pandemic in Taiwan. BMC Public Health.

2013 Jul 12;13:646. doi: 10.1186/1471-2458-13-646. PMID: 23849163; PMCID:

PMC3733801.

242: Cantarutti A, Rea F, Donà D, Cantarutti L, Passarella A, Scamarcia A,

Lundin R, Damiani V, Giaquinto C, Corrao G. Preventing recurrent acute otitis

media with Streptococcus salivarius 24SMB and Streptococcus oralis 89a five

months intermittent treatment: An observational prospective cohort study. Int J

Pediatr Otorhinolaryngol. 2020 May;132:109921. doi:

10.1016/j.ijporl.2020.109921. Epub 2020 Feb 5. PMID: 32062496.

243: Hooiveld M, van de Groep T, Verheij TJ, van der Sande MA, Verheij RA,

Tacken MA, van Essen GA. Prescription of antiviral drugs during the 2009

influenza pandemic: an observational study using electronic medical files of

general practitioners in the Netherlands. BMC Pharmacol Toxicol. 2013 Oct

21;14:55. doi: 10.1186/2050-6511-14-55. PMID: 24143932; PMCID: PMC3854647.

244: Eythorsson E, Sigurdsson S, Hrafnkelsson B, Erlendsdóttir H, Haraldsson Á,

Kristinsson KG. Impact of the 10-valent pneumococcal conjugate vaccine on

antimicrobial prescriptions in young children: a whole population study. BMC

Infect Dis. 2018 Oct 4;18(1):505. doi: 10.1186/s12879-018-3416-y. PMID:

30286726; PMCID: PMC6172799.

245: Magidson JF, Iyer HS, Regenauer KS, Grelotti DJ, Dietrich JJ, Courtney I,

Tshabalala G, Orrell C, Gray GE, Bangsberg DR, Katz IT. Recreational ART use

among individuals living with HIV/AIDS in South Africa: Examining longitudinal

ART initiation and viral suppression. Drug Alcohol Depend. 2019 May

1;198:192-198. doi: 10.1016/j.drugalcdep.2019.02.009. Epub 2019 Mar 22. PMID:

30953938; PMCID: PMC6644059.

246: Craxì A, Piccinino F, Ciancio A, Iannacone C, Deodato B, Golotta C, Ascione

A. Real-world outcomes in patients with chronic hepatitis C: primary results of

the PROBE study. Eur J Gastroenterol Hepatol. 2014 Apr;26(4):388-95. doi:

10.1097/MEG.0000000000000039. PMID: 24569819.

247: Kuo SC, Chen YT, Lee YT, Fan NW, Chen SJ, Li SY, Liu CJ, Chen TL, Chen TJ,

Fung CP. Association between recent use of fluoroquinolones and rhegmatogenous

retinal detachment: a population-based cohort study. Clin Infect Dis. 2014

Jan;58(2):197-203. doi: 10.1093/cid/cit708. Epub 2013 Oct 28. PMID: 24170197.

248: van Hecke O, Fuller A, Bankhead C, Jenkins-Jones S, Francis N, Moore M,

Butler C, Wang K. Antibiotic exposure and 'response failure' for subsequent

respiratory tract infections: an observational cohort study of UK preschool

children in primary care. Br J Gen Pract. 2019 Aug 29;69(686):e638-e646. doi:

10.3399/bjgp19X705089. PMID: 31405831; PMCID: PMC6692084.

249: Fortanier AC, Venekamp RP, Stellato RK, Sanders EAM, Damoiseaux RAMJ, Hoes

AW, Schilder AM. Outpatient antibiotic use in Dutch infants after 10-valent

pneumococcal vaccine introduction: a time-series analysis. BMJ Open. 2018 Jun

30;8(6):e020619. doi: 10.1136/bmjopen-2017-020619. PMID: 29961011; PMCID:

PMC6042577.

250: Poon LM, Jin J, Chee YL, Ding Y, Lee YM, Chng WJ, Chai LY, Tan LK, Hsu LY.

Risk factors for adverse outcomes and multidrug-resistant Gram-negative

bacteraemia in haematology patients with febrile neutropenia in a Singaporean

university hospital. Singapore Med J. 2012 Nov;53(11):720-5. PMID: 23192498.

251: Berthe-Aucejo A, Girard D, Lorrot M, Bellettre X, Faye A, Mercier JC, Brion

F, Bourdon O, Prot-Labarthe S. Evaluation of frequency of paediatric oral liquid

medication dosing errors by caregivers: amoxicillin and josamycin. Arch Dis

Child. 2016 Apr;101(4):359-64. doi: 10.1136/archdischild-2015-309426. Epub 2016

Jan 4. PMID: 26729746.

252: Jiwa M, Krejany CJ, Kanjo E, Leeb A, Peters IJ. Symptom profile of patients

receiving antibiotics for upper respiratory tract infections in general

practice: an observational study using smartphone technology. Fam Pract. 2019

Oct 8;36(5):560-567. doi: 10.1093/fampra/cmy134. PMID: 30649332.

253: Abou-Shaaban M, Ali AA, Rao PG, Majid A. Drug utilization review of

cephalosporins in a secondary care hospital in United Arab Emirates. Int J Clin

Pharm. 2016 Dec;38(6):1367-1371. doi: 10.1007/s11096-016-0392-4. Epub 2016 Nov

5. PMID: 27817172.

254: Ruzicka DJ, Imai K, Takahashi K, Naito T. Greater burden of chronic

comorbidities and co-medications among people living with HIV versus people

without HIV in Japan: A hospital claims database study. J Infect Chemother. 2019

Feb;25(2):89-95. doi: 10.1016/j.jiac.2018.10.006. Epub 2018 Nov 3. PMID:

30396821.

255: Li J, Kang-Birken SL, Mathews SK, Kenner CE, Fitzgibbons LN. Role of rapid

diagnostics for viral respiratory infections in antibiotic prescribing decision

in the emergency department. Infect Control Hosp Epidemiol. 2019

Sep;40(9):974-978. doi: 10.1017/ice.2019.166. Epub 2019 Jun 28. PMID: 31250772.

256: Lepelletier D, Pinaud V, Le Conte P, Bourigault C, Asseray N, Ballereau F,

Caillon J, Ferron C, Righini C, Batard E, Potel G; French PTA Study Group. Is

there an association between prior anti-inflammatory drug exposure and

occurrence of peritonsillar abscess (PTA)? A national multicenter prospective

observational case-control study. Eur J Clin Microbiol Infect Dis. 2017

Jan;36(1):57-63. doi: 10.1007/s10096-016-2770-1. Epub 2016 Sep 7. PMID:

27604832.

257: Piovani D, Clavenna A, Bonati M; PeFAB group. Review of Italian primary

care paediatricians identifies 38 commonly prescribed drugs for children. Acta

Paediatr. 2014 Dec;103(12):e532-7. doi: 10.1111/apa.12783. Epub 2014 Oct 2.

PMID: 25164591.

258: Micallef C, Aliyu SH, Santos R, Brown NM, Rosembert D, Enoch DA.

Introduction of an antifungal stewardship programme targeting high-cost

antifungals at a tertiary hospital in Cambridge, England. J Antimicrob

Chemother. 2015;70(6):1908-11. doi: 10.1093/jac/dkv040. Epub 2015 Feb 25. PMID:

25722302.

259: Eisenberger U, Budde K, Lehner F, Sommerer C, Reinke P, Witzke O, Wüthrich

RP, Stahl R, Heller K, Suwelack B, Mühlfeld A, Hauser IA, Nadalin S, Porstner M,

Arns W; ZEUS Study Investigators. Histological findings to five years after

early conversion of kidney transplant patients from cyclosporine to everolimus:

an analysis from the randomized ZEUS study. BMC Nephrol. 2018 Jun 28;19(1):154.

doi: 10.1186/s12882-018-0950-1. PMID: 29954336; PMCID: PMC6025714.

260: Haenssgen MJ, Charoenboon N, Zanello G, Mayxay M, Reed-Tsochas F, Lubell Y,

Wertheim H, Lienert J, Xayavong T, Khine Zaw Y, Thepkhamkong A, Sithongdeng N,

Khamsoukthavong N, Phanthavong C, Boualaiseng S, Vongsavang S, Wibunjak K, Chai-

In P, Thavethanutthanawin P, Althaus T, Greer RC, Nedsuwan S, Wangrangsimakul T,

Limmathurotsakul D, Elliott E, Ariana P. Antibiotic knowledge, attitudes and

practices: new insights from cross-sectional rural health behaviour surveys in

low-income and middle-income South-East Asia. BMJ Open. 2019 Aug

20;9(8):e028224. doi: 10.1136/bmjopen-2018-028224. PMID: 31434769; PMCID:

PMC6707701.

261: McIsaac WJ, Moineddin R, Gágyor I, Mazzulli T. External validation study of

a clinical decision aid to reduce unnecessary antibiotic prescriptions in women

with acute cystitis. BMC Fam Pract. 2017 Oct 2;18(1):89. doi:

10.1186/s12875-017-0660-y. PMID: 28969592; PMCID: PMC5625594.

262: O'Brien K, Bellis TW, Kelson M, Hood K, Butler CC, Edwards A. Clinical

predictors of antibiotic prescribing for acutely ill children in primary care:

an observational study. Br J Gen Pract. 2015 Sep;65(638):e585-92. doi:

10.3399/bjgp15X686497. PMID: 26324495; PMCID: PMC4540398.

263: Zhang Z, Zhan X, Zhou H, Sun F, Zhang H, Zwarenstein M, Liu Q, Li Y, Yan W.

Antibiotic prescribing of village doctors for children under 15 years with upper

respiratory tract infections in rural China: A qualitative study. Medicine

(Baltimore). 2016 Jun;95(23):e3803. doi: 10.1097/MD.0000000000003803. Erratum

in: Medicine (Baltimore). 2016 Jul 18;95(28):e0916. doi:

10.1097/01.md.0000489580.04709.16. PMID: 27281082; PMCID: PMC4907660.

264: Palmu AA, Rinta-Kokko H, Nohynek H, Nuorti JP, Jokinen J. Impact of

National Ten-Valent Pneumococcal Conjugate Vaccine Program on Reducing

Antimicrobial Use and Tympanostomy Tube Placements in Finland. Pediatr Infect

Dis J. 2018 Jan;37(1):97-102. doi: 10.1097/INF.0000000000001810. PMID: 29088026.

265: Lau WC, Murray M, El-Turki A, Saxena S, Ladhani S, Long P, Sharland M, Wong

IC, Hsia Y. Impact of pneumococcal conjugate vaccines on childhood otitis media

in the United Kingdom. Vaccine. 2015 Sep 22;33(39):5072-9. doi:

10.1016/j.vaccine.2015.08.022. Epub 2015 Aug 19. PMID: 26297875.

266: Waade RB, Molden E, Martinsen MI, Hermann M, Ranhoff AH. Psychotropics and

weak opioid analgesics in plasma samples of older hip fracture patients -

detection frequencies and consistency with drug records. Br J Clin Pharmacol.

2017 Jul;83(7):1397-1404. doi: 10.1111/bcp.13244. Epub 2017 Mar 9. PMID:

28268245; PMCID: PMC5465332.

267: D'Haens G, Reinisch W, Colombel JF, Panes J, Ghosh S, Prantera C, Lindgren

S, Hommes DW, Huang Z, Boice J, Huyck S, Cornillie F; ENCORE investigators.

Five-year Safety Data From ENCORE, a European Observational Safety Registry for

Adults With Crohn's Disease Treated With Infliximab [Remicade®] or Conventional

Therapy. J Crohns Colitis. 2017 Jun 1;11(6):680-689. doi: 10.1093/ecco-

jcc/jjw221. PMID: 28025307.

268: Weterings V, Veenemans J, van Rijen M, Kluytmans J. Prevalence of nasal

carriage of methicillin-resistant Staphylococcus aureus in patients at hospital

admission in The Netherlands, 2010-2017: an observational study. Clin Microbiol

Infect. 2019 Nov;25(11):1428.e1-1428.e5. doi: 10.1016/j.cmi.2019.03.012. Epub

2019 Mar 27. PMID: 30928560.

269: Gunnlaugsdottir MR, Linnet K, Jonsson JS, Blondal AB. Encouraging rational

antibiotic prescribing behaviour in primary care - prescribing practice among

children aged 0-4 years 2016-2018: an observational study. Scand J Prim Health

Care. 2021 Sep;39(3):373-381. doi: 10.1080/02813432.2021.1958506. Epub 2021 Aug

4. PMID: 34348560; PMCID: PMC8475099.

270: Mohialdin D, Abu-Farha R, ALkhawaldeh R, Zawiah M, Abu Hammour K. Audit of

adherence to international guidelines (IDSA) in the treatment of infectious

meningitis in pediatric patients in Jordan. Curr Med Res Opin. 2024

Mar;40(3):423-430. doi: 10.1080/03007995.2024.2314738. Epub 2024 Feb 13. PMID:

38308446.

271: Miller K, McGraw MA, Tomsey A, Hegde GG, Shang J, O'Neill JM, Venkat A.

Pharmacist addition to the post-ED visit review of discharge antimicrobial

regimens. Am J Emerg Med. 2014 Oct;32(10):1270-4. doi:

10.1016/j.ajem.2014.07.033. Epub 2014 Aug 2. PMID: 25171797.

272: Manyando C, Njunju EM, Chileshe J, Siziya S, Shiff C. Rapid diagnostic

tests for malaria and health workers' adherence to test results at health

facilities in Zambia. Malar J. 2014 May 2;13:166. doi: 10.1186/1475-2875-13-166.

PMID: 24885996; PMCID: PMC4026818.

273: Hu HH, Chiou CC, Cheng MF, Chen YS, Sheu SK, Wu TC, Huang IF. The clinical

outcomes of antimicrobial therapy in pediatric patients with nontyphoid

salmonellosis with different levels of severity. Clin Pediatr (Phila). 2014

Sep;53(10):967-74. doi: 10.1177/0009922814540792. Epub 2014 Jul 7. PMID:

25006111.

274: Lisspers K, Johansson G, Jansson C, Larsson K, Stratelis G, Hedegaard M,

Ställberg B. Improvement in COPD management by access to asthma/COPD clinics in

primary care: data from the observational PATHOS study. Respir Med. 2014

Sep;108(9):1345-54. doi: 10.1016/j.rmed.2014.06.002. Epub 2014 Jun 17. PMID:

25002194.

275: Samuelsson A, Isaksson B, Hanberger H, Olhager E. Late-onset neonatal

sepsis, risk factors and interventions: an analysis of recurrent outbreaks of

Serratia marcescens, 2006-2011. J Hosp Infect. 2014 Jan;86(1):57-63. doi:

10.1016/j.jhin.2013.09.017. Epub 2013 Oct 23. PMID: 24332914.

276: Aldeyab MA, McElnay JC, Scott MG, Lattyak WJ, Darwish Elhajji FW, Aldiab

MA, Magee FA, Conlon G, Kearney MP. A modified method for measuring antibiotic

use in healthcare settings: implications for antibiotic stewardship and

benchmarking. J Antimicrob Chemother. 2014 Apr;69(4):1132-41. doi:

10.1093/jac/dkt458. Epub 2013 Nov 11. PMID: 24222612.

277: Kim DY, Yoon KT, Kim W, Lee JI, Hong SH, Lee D, Jang JW, Choi JW, Kim I,

Paik YH. Estimation of direct medical cost related to the management of chronic

hepatitis C and its complications in South Korea. Medicine (Baltimore). 2016

Jul;95(30):e3896. doi: 10.1097/MD.0000000000003896. PMID: 27472670; PMCID:

PMC5265806.

278: Tian SY, Silverman ED, Pullenayegum E, Brown PE, Beyene J, Feldman BM.

Comparative Effectiveness of Mycophenolate Mofetil for the Treatment of

Juvenile-Onset Proliferative Lupus Nephritis. Arthritis Care Res (Hoboken). 2017

Dec;69(12):1887-1894. doi: 10.1002/acr.23215. Epub 2017 Nov 2. PMID: 28182833.

279: Charani E, Gharbi M, Hickson M, Othman S, Alfituri A, Frost G, Holmes A.

Lack of weight recording in patients being administered narrow therapeutic index

antibiotics: a prospective cross-sectional study. BMJ Open. 2015 Apr

2;5(4):e006092. doi: 10.1136/bmjopen-2014-006092. PMID: 25838504; PMCID:

PMC4390734.

280: Jamal Mohamed T, Teeraananchai S, Kerr S, Phongsamart W, Nik Yusoff NK,

Hansudewechakul R, Ly PS, Nguyen LV, Sudjaritruk T, Lumbiganon P, Do VC,

Kurniati N, Kumarasamy N, Wati DK, Fong MS, Nallusamy R, Kariminia A, Sohn AH.

Short Communication: Impact of Viral Load Use on Treatment Switch in Perinatally

HIV-Infected Children in Asia. AIDS Res Hum Retroviruses. 2017

Mar;33(3):230-233. doi: 10.1089/AID.2016.0039. Epub 2016 Oct 31. PMID: 27758114;

PMCID: PMC5333561.

281: Silfwerbrand E, Verma S, Sjökvist C, Stålsby Lundborg C, Sharma M.

Diagnose-Specific Antibiotic Prescribing Patterns at Otorhinolaryngology

Inpatient Departments of Two Private Sector Healthcare Facilities in Central

India: A Five-Year Observational Study. Int J Environ Res Public Health. 2019

Oct 23;16(21):4074. doi: 10.3390/ijerph16214074. PMID: 31652748; PMCID:

PMC6862163.

282: Bedouch P, Sylvoz N, Charpiat B, Juste M, Roubille R, Rose FX, Bosson JL,

Conort O, Allenet B; French Society of Clinical Pharmacy's Act-IP© Group. Trends

in pharmacists' medication order review in French hospitals from 2006 to 2009:

analysis of pharmacists' interventions from the Act-IP© website observatory. J

Clin Pharm Ther. 2015 Feb;40(1):32-40. doi: 10.1111/jcpt.12214. Epub 2014 Oct

10. PMID: 25303720.

283: Mouloudi E, Massa E, Piperidou M, Papadopoulos S, Iosifidis E, Roilides I,

Theodoridou T, Kydona C, Fouzas I, Imvrios G, Papanikolaou V, Gritsi-Gerogianni

N. Tigecycline for treatment of carbapenem-resistant Klebsiella pneumoniae

infections after liver transplantation in the intensive care unit: a 3-year

study. Transplant Proc. 2014 Nov;46(9):3219-21. doi:

10.1016/j.transproceed.2014.09.160. PMID: 25420864.

284: Posteraro B, Tumbarello M, De Pascale G, Liberto E, Vallecoccia MS, De

Carolis E, Di Gravio V, Trecarichi EM, Sanguinetti M, Antonelli M. (1,3)-β-d-

Glucan-based antifungal treatment in critically ill adults at high risk of

candidaemia: an observational study. J Antimicrob Chemother. 2016

Aug;71(8):2262-9. doi: 10.1093/jac/dkw112. Epub 2016 Apr 28. PMID: 27125554.

285: Çelik Ekinci S, Yenilmez E, Akengin Öcal G, Sönmezer MÇ, Tarakçı A, Aygün

C, Akdağ D, Seyman D, Aşık C, Zerdali E, Yılmaz Karadağ F, Kaya Ş, Çelik M,

Çifci Ş, Yıldız İE, Çölkesen F, Akgül F, Aldemir Ö, Bozdağ M, Özer D, Hızmalı L,

Canbolat Ünlü E, Gür Altunay D, Şahin A, Ünlü G, Gençalioğlu AE, Tekin Şahin S,

Özdemir Y, Ünlü S, Singil S, Altıntaş J, Akkaya Işık S, Gül Ö, Tuna N, Şimşek S,

Özgüler M, Elbir Kılıç P, Işık ME, Karakuş A, Kıratlı K, Yardımcı AC, Volkan S,

Olçar Y, Çakır Y, Özer Yılmaz N, Karaayvaz S, Batırel A, Duran ZC, Raşa HK, Köse

Ş. Surgical Antimicrobial Prophylaxis Compliance in Turkey: Data from the

Prospective, Observational, Multicenter Survey Including 7,978 Surgical

Patients. Surg Infect (Larchmt). 2024 Apr;25(3):231-239. doi:

10.1089/sur.2023.243. PMID: 38588521.

286: Howitz MF, Harboe ZB, Ingels H, Valentiner-Branth P, Mølbak K, Djurhuus BD.

A nationwide study on the impact of pneumococcal conjugate vaccination on

antibiotic use and ventilation tube insertion in Denmark 2000-2014. Vaccine.

2017 Oct 13;35(43):5858-5863. doi: 10.1016/j.vaccine.2017.09.006. Epub 2017 Sep

18. PMID: 28928078.

287: Neilly MDJ, Guthrie B, Hernandez Santiago V, Vadiveloo T, Donnan PT,

Marwick CA. Has primary care antimicrobial use really been increasing?

Comparison of changes in different prescribing measures for a complete

geographic population 1995-2014. J Antimicrob Chemother. 2017 Oct

1;72(10):2921-2930. doi: 10.1093/jac/dkx220. PMID: 29091203.

288: Costenaro P, Penazzato M, Lundin R, Rossi G, Massavon W, Patel D, Nabachwa

S, Franceschetto G, Morelli E, Bilardi D, Nannyonga MM, Atzori A, Mastrogiacomo

ML, Mazza A, Putoto G, Giaquinto C. Predictors of Treatment Failure in HIV-

Positive Children Receiving Combination Antiretroviral Therapy: Cohort Data From

Mozambique and Uganda. J Pediatric Infect Dis Soc. 2015 Mar;4(1):39-48. doi:

10.1093/jpids/piu032. Epub 2014 May 3. PMID: 26407356.

289: Yang P, Huang G, Du L, Ye Z, Hu K, Wang C, Qi J, Liang L, Wu L, Cao Q,

Kijlstra A. Long-Term Efficacy and Safety of Interferon Alpha-2a in the

Treatment of Chinese Patients with Behçet's Uveitis Not Responding to

Conventional Therapy. Ocul Immunol Inflamm. 2019;27(1):7-14. doi:

10.1080/09273948.2017.1384026. Epub 2017 Oct 17. PMID: 29040035.

290: Ibeneme GC, Nwaneri AC, Ibeneme SC, Ezenduka P, Strüver V, Fortwengel G,

Okoye IJ. Mothers' perception of recovery and satisfaction with patent medicine

dealers' treatment of childhood febrile conditions in rural communities. Malar

J. 2016 Jun 28;15:336. doi: 10.1186/s12936-016-1384-5. PMID: 27352902; PMCID:

PMC4924311.

291: Gauld NJ, Zeng IS, Ikram RB, Thomas MG, Buetow SA. Treatment of

uncomplicated cystitis: analysis of prescribing in New Zealand. N Z Med J. 2016

Jul 1;129(1437):55-63. PMID: 27362599.

292: Angoulvant F, Pereira M, Perreaux F, Soussan V, Pham LL, Trieu TV, Cojocaru

B, Guedj R, Cohen R, Alberti C, Gajdos V. Impact of unlabeled French antibiotic

guidelines on antibiotic prescriptions for acute respiratory tract infections in

7 Pediatric Emergency Departments, 2009-2012. Pediatr Infect Dis J. 2014

Mar;33(3):330-3. doi: 10.1097/INF.0000000000000125. PMID: 24168976.

293: Martinez KA, Rood M, Jhangiani N, Boissy A, Rothberg MB. Antibiotic

Prescribing for Respiratory Tract Infections and Encounter Length: An

Observational Study of Telemedicine. Ann Intern Med. 2019 Feb 19;170(4):275-277.

doi: 10.7326/M18-2042. Epub 2018 Oct 2. PMID: 30285078.

**Non-prescription use of antibiotics children [title]**

*Publish or Perish 8.2.3944.8118 (basic report)
WinPosix (x64) edition, running on WinPosix 10.0.22000 (x64)*

**Search terms**

**Title words:** Non-prescription use of antibiotics children
**Years:** all

**Data retrieval**

**Data source:** Scopus
**Search date:** 2024-07-01 16:43:36 +0300
**Cache date:** 2024-07-01 13:42:07 +0300
**Search result:** [0] No error

***Important:*** *This data source returns only one author per article; this affects the calculation of per-author metrics.*

**Metrics**

**Reference date:** 2024-07-01 13:42:07 +0300
**Publication years:** 2007-2024
**Citation years:** 17 (2007-2024)
**Papers:** 8
**Citations:** 59
**Citations/year:** 3.47 (acc1=4, acc2=2, acc5=1, acc10=0, acc20=0)
**Citations/paper:** 7.38
**Authors/paper:** 1.00/1.0/1 (mean/median/mode)
**Age-weighted citation rate:** 11.00 (sqrt=3.32), 11.00/author
**Hirsch h-index:** 4 (a=3.69, m=0.24, 59 cites=100.0% coverage)
**Egghe g-index:** 7 (g/h=1.75, 59 cites=100.0% coverage)
**PoP hI,norm:** 4
**PoP hI,annual:** 0.24
**Fassin hA-index:** 2

**Results**

1. J. Chang (2018) **Non-prescription use of antibiotics among children in urban China: a cross-sectional survey of knowledge, attitudes, and practices**. *Expert Review of Anti-Infective Therapy* 16(2), pp. 163-172, ISSN 1478-7210, doi:10.1080/14787210.2018.1425616, cited by 37 (6.17 per year)

2. C.C. Chen (2011) **Non-adherence to antibiotic prescription guidelines in treating urinary tract infection of children: A population-based study in Taiwan**. *Journal of Evaluation in Clinical Practice* 17(6), pp. 1030-1035, ISSN 1356-1294, doi:10.1111/j.1365-2753.2010.01469.x, cited by 13 (1.00 per year)

3. M.T. Amin (2022) **Over prescription of antibiotics in children with acute upper respiratory tract infections: A study on the knowledge, attitude and practices of non-specialized physicians in Egypt**. *PLoS ONE* 17(11), ISSN 1932-6203, doi:10.1371/journal.pone.0277308, cited by 5 (2.50 per year)

4. Y. Zhu (2021) **Non-prescription antibiotic use for cough among Chinese children under 5 years of age: A community-based cross-sectional study**. *BMJ Open* 11(12), ISSN 2044-6055, doi:10.1136/bmjopen-2021-051372, cited by 4 (1.33 per year)

5. M.M. Bezie (2024) **Factors associated with the use of antibiotics for children presenting with illnesses with fever and cough obtained from prescription and non-prescription sources: a cross-sectional study of data for 37 sub-Saharan African countries**. *BMC Public Health* 24(1), ISSN 1471-2458, doi:10.1186/s12889-024-18490-1

6. S. Mattila (2023) **Point-of-care testing for respiratory pathogens does not change antibiotic prescriptions in children: Findings of an RCT**. *Quaderni ACP* 30(3), ISSN 2039-1374, doi:10.53141/PEQACP.2023.3.N1

7. G. Mas-Dalmau (2023) **A trial-based cost-effectiveness analysis of antibiotic prescription strategies for non-complicated respiratory tract infections in children**. *BMC Pediatrics* 23(1), ISSN 1471-2431, doi:10.1186/s12887-023-04235-3

8. M. Amado (2007) **The prescription of antibiotics in the non-complicated acute otitis media in children can be restricted in a safe way to the cases that do not respond to the symptomatic treatment**. *FMC Formacion Medica Continuada en Atencion Primaria* 14(3), p. 182, ISSN 1134-2072, doi:10.1016/S1134-2072(07)71586-4

**Self-medication antibiotics children [title]**

*Publish or Perish 8.2.3944.8118 (basic report)
WinPosix (x64) edition, running on WinPosix 10.0.22000 (x64)*

**Search terms**

**Title words:** Self-medication antibiotics children
**Years:** all

**Data retrieval**

**Data source:** Scopus
**Search date:** 2024-07-01 16:48:03 +0300
**Cache date:** 2024-07-01 13:46:04 +0300
**Search result:** [0] No error

***Important:*** *This data source returns only one author per article; this affects the calculation of per-author metrics.*

**Metrics**

**Reference date:** 2024-07-01 13:46:04 +0300
**Publication years:** 2000-2024
**Citation years:** 24 (2000-2024)
**Papers:** 16
**Citations:** 293
**Citations/year:** 12.21 (acc1=9, acc2=8, acc5=4, acc10=1, acc20=0)
**Citations/paper:** 18.31
**Authors/paper:** 1.00/1.0/1 (mean/median/mode)
**Age-weighted citation rate:** 48.74 (sqrt=6.98), 48.74/author
**Hirsch h-index:** 7 (a=5.98, m=0.29, 281 cites=95.9% coverage)
**Egghe g-index:** 16 (g/h=2.29, 293 cites=100.0% coverage)
**PoP hI,norm:** 7
**PoP hI,annual:** 0.29
**Fassin hA-index:** 4

**Results**

1. P. Bi (2000) **Family self-medication and antibiotics abuse for children and juveniles in a Chinese city**. *Social Science and Medicine* 50(10), pp. 1445-1450, ISSN 0277-9536, doi:10.1016/S0277-9536(99)00304-4, cited by 121 (5.04 per year)

2. G.A.E. Ekambi (2019) **Knowledge, practices and attitudes on antibiotics use in Cameroon: Self-medication and prescription survey among children, adolescents and adults in private pharmacies**. *PLoS ONE* 14(2), ISSN 1932-6203, doi:10.1371/journal.pone.0212875, cited by 66 (13.20 per year)

3. C. Sun (2019) **Influence of leftover antibiotics on self-medication with antibiotics for children: A cross-sectional study from three Chinese provinces**. *BMJ Open* 9(12), ISSN 2044-6055, doi:10.1136/bmjopen-2019-033679, cited by 34 (6.80 per year)

4. F. Bert (2022) **Antibiotics Self Medication among Children: A Systematic Review**. *Antibiotics* 11(11), ISSN 2079-6382, doi:10.3390/antibiotics11111583, cited by 18 (9.00 per year)

5. T.L. Mukattash (2020) **Parental self-medication of antibiotics for children in Jordan**. *Journal of Pharmaceutical Health Services Research* 11(1), pp. 75-80, ISSN 1759-8885, doi:10.1111/jphs.12331, cited by 16 (4.00 per year)

6. J. Xu (2020) **Parental self-medication with antibiotics for children promotes antibiotic over-prescribing in clinical settings in China**. *Antimicrobial Resistance and Infection Control* 9(1), ISSN 2047-2994, doi:10.1186/s13756-020-00811-9, cited by 14 (3.50 per year)

7. A. Jasim (2014) **Parental self medication of antibiotics for children in Baghdad city**. *International Journal of Pharmacy and Pharmaceutical Sciences* 6(10), pp. 485-489, ISSN 0975-1491, cited by 12 (1.20 per year)

8. J. Wu (2021) **Prevalence of antibiotic self-medication behavior and related factors among children aged 0 to 5 years**. *Expert Review of Anti-Infective Therapy* 19(9), pp. 1157-1164, ISSN 1478-7210, doi:10.1080/14787210.2021.1882303, cited by 7 (2.33 per year)

9. P. Paulsamy (2023) **Parental health-seeking behavior on self-medication, antibiotic use, and antimicrobial resistance in children**. *Saudi Pharmaceutical Journal* 31(9), ISSN 1319-0164, doi:10.1016/j.jsps.2023.101712, cited by 3 (3.00 per year)

10. J. Nazari (2022) **Prevalence and determinants of self-medication consumption of antibiotics in children in Iran: A population-based cross-sectional study, 2018–19**. *PLoS ONE* 17(12), ISSN 1932-6203, doi:10.1371/journal.pone.0278843, cited by 1 (0.50 per year)

11. Y.R. Kadam (2018) **Parental use of antibiotics as self medication to their school going children: A cross sectional study**. *Journal of Krishna Institute of Medical Sciences University* 7(1), pp. 16-24, ISSN 2231-4261, cited by 1 (0.17 per year)

12. F.K. Hashmi (2024) **Parents’ behaviour toward antibiotic self-medication in children and incidence of resistance: a cross-sectional study from Punjab, Pakistan**. *Family Medicine and Primary Care Review* 26(1), pp. 39-50, ISSN 1734-3402, doi:10.5114/fmpcr.2024.134701

13. W. Qu (2023) **Self-Medication with Antibiotics Among Children in China: A Cross-Sectional Study of Parents’ Knowledge, Attitudes, and Practices**. *Infection and Drug Resistance* 16, pp. 7683-7694, ISSN 1178-6973, doi:10.2147/IDR.S431034

14. D.A.E. Pitaloka (2023) **Development and Validation of Questionnaire to Measure Parents’ Knowledge, Attitude, and Practice on Self-Medication of Children with Antibiotics in Bandung, Indonesia**. *Infection and Drug Resistance* 16, pp. 6111-6120, ISSN 1178-6973, doi:10.2147/IDR.S426313

15. D. Pei (2023) **The Role of Uncertainty and Negative Emotion in Chinese Parents’ Self-Medication of Children with Antibiotics**. *International Journal of Environmental Research and Public Health* 20(16), ISSN 1661-7827, doi:10.3390/ijerph20166603

16. H.B. Janjua (2023) **The Factors Leading to Parental Self Medication of Antibiotics in Children**. *Medical Forum Monthly* 34(1), pp. 60-63, ISSN 1029-385X

**self-medication antibiotics children**

*Publish or Perish 8.2.3944.8118 (basic report)
WinPosix (x64) edition, running on WinPosix 10.0.22000 (x64)*

**Search terms**

**Keywords:** self-medication antibiotics children
**Years:** all

**Data retrieval**

**Data source:** PubMed
**Search date:** 2024-07-01 16:52:53 +0300
**Cache date:** 2024-07-01 13:50:53 +0300
**Search result:** [0] No error

***Important:*** *This data source does not provide citation counts.*

**Metrics**

**Reference date:** 2024-07-01 13:50:53 +0300
**Publication years:** 1976-2024
**Citation years:** 48 (1976-2024)
**Papers:** 106
**Citations:** 0
**Citations/year:** 0.00 (acc1=0, acc2=0, acc5=0, acc10=0, acc20=0)
**Citations/paper:** 0.00
**Authors/paper:** 5.14/5.0/5 (mean/median/mode)
**Age-weighted citation rate:** 0.00 (sqrt=0.00), 0.00/author
**Hirsch h-index:** 0 (a=0.00, m=0.00, 0 cites=0.0% coverage)
**Egghe g-index:** 0 (g/h=0.00, 0 cites=0.0% coverage)
**PoP hI,norm:** 0
**PoP hI,annual:** 0.00
**Fassin hA-index:** 0

**Results**

Maike Maria Lamshöft, Edwin Liheluka, Greta Ginski, John P A Lusingu, Daniel Minja, Samwel Gesase, Joyce Mbwana, George Gesase, Lydia Rautman, Wibke Loag, Jürgen May, Denise Dekker, Ralf Krumkamp (2024) **Understanding pre-hospital disease management of fever and diarrhoea in children-Care pathways in rural Tanzania.**. *Tropical medicine & international health : TM & IH*, ISSN 1365-3156, doi:10.1111/tmi.14022

Hasnae Elhaddadi, Amal Hamami, Anane Sara, Aziza Elouali, Abdeladim Babakhouya, Maria Rkain (2024) **Prevalence and Determinants of the Use of Antibiotics by Self-Medication in the Pediatric Population in Oujda, Morocco.**. *Cureus* 16(5), ISSN 2168-8184, doi:10.7759/cureus.60126

Theopista Lotto, Sabine Renggli, Eliangiringa Kaale, Honorati Masanja, Beatrice Ternon, Laurent Arthur Décosterd, Valérie D'Acremont, Blaise Genton, Alexandra V Kulinkina (2024) **Prevalence and predictors of residual antibiotics in children's blood in community settings in Tanzania.**. *Clinical microbiology and infection : the official publication of the European Society of Clinical Microbiology and Infectious Diseases*, ISSN 1469-0691, doi:10.1016/j.cmi.2024.05.004

Dan Waitzberg, Francisco Guarner, Iva Hojsak, Gianluca Ianiro, D Brent Polk, Harry Sokol (2024) **Can the Evidence-Based Use of Probiotics (Notably Saccharomyces boulardii CNCM I-745 and Lactobacillus rhamnosus GG) Mitigate the Clinical Effects of Antibiotic-Associated Dysbiosis?**. *Advances in therapy* 41(3), pp. 901-914, ISSN 1865-8652, doi:10.1007/s12325-024-02783-3

Lusajo Shitindi, Omary Issa, Baraka P Poyongo, Pius Gerald Horumpende, Godeliver A Kagashe, Raphael Z Sangeda (2023) **Comparison of knowledge, attitude, practice and predictors of self-medication with antibiotics among medical and non-medical students in Tanzania.**. *Frontiers in pharmacology* 14, p. 1301561, ISSN 1663-9812, doi:10.3389/fphar.2023.1301561

Bintou Diarra, Ibréhima Guindo, Boī Koné, Maīmouna Dembélé, Ibrahim Cissé, Souleymane Thiam, Kadidia Konaté, Mamadou Tékété, Almoustapha Maīga, Oumou Maīga, Lassina Timbiné, Abdoulaye Djimde (2024) **High frequency of antimicrobial resistance in Salmonella and Escherichia coli causing diarrheal diseases at the Yirimadio community health facility, Mali.**. *BMC microbiology* 24(1), p. 35, ISSN 1471-2180, doi:10.1186/s12866-024-03198-4

Wenjie Qu, Xinyu Wang, Yufei Liu, Jinfeng Mao, Mengchi Liu, Yaqin Zhong, Bella Gao, Miaomiao Zhao, Yuexia Gao (2023) **Self-Medication with Antibiotics Among Children in China: A Cross-Sectional Study of Parents' Knowledge, Attitudes, and Practices.**. *Infection and drug resistance* 16, pp. 7683-7694, ISSN 1178-6973, doi:10.2147/IDR.S431034

Petruța Tarciuc, Alina Duduciuc, Sergiu Ioachim Chirila, Valeria Herdea, Oana Rosu, Andreea Varga, Ileana Ioniuc, Smaranda Diaconescu (2023) **Assessing the Effects of Medical Information on Parental Self-Medication Behaviors for Children's Health: A Comparative Analysis.**. *Medicina (Kaunas, Lithuania)* 59(12), ISSN 1648-9144, doi:10.3390/medicina59122093

Miradije Imeri, Shaip Krasniqi, Lul Raka, Isme Humolli, Kreshnik Hoti, Zana Imeri, Valbona Zhjeqi (2023) **Evaluation of parents' attitudes and practices related to antibiotic use for their children in Kosovo: a cross-sectional survey.**. *Journal of pharmaceutical policy and practice* 16(1), p. 168, ISSN 2052-3211, doi:10.1186/s40545-023-00676-4

Dian Ayu Eka Pitaloka, Ikhwan Yuda Kusuma, Ariani Insyirah, Anisa Nabilah Oktariani, Hening Pratiwi, Nayla Majida Alfarafisa (2023) **Development and Validation of Questionnaire to Measure Parents' Knowledge, Attitude, and Practice on Self-Medication of Children with Antibiotics in Bandung, Indonesia.**. *Infection and drug resistance* 16, pp. 6111-6120, ISSN 1178-6973, doi:10.2147/IDR.S426313

Di Pei, Gary Kreps, Xiaoquan Zhao (2023) **The Role of Uncertainty and Negative Emotion in Chinese Parents' Self-Medication of Children with Antibiotics.**. *International journal of environmental research and public health* 20(16), ISSN 1660-4601, doi:10.3390/ijerph20166603

Premalatha Paulsamy, Krishnaraju Venkatesan, Shadia Hamoud Alshahrani, Maha Hamed Mohamed Ali, Kousalya Prabahar, Vinoth Prabhu Veeramani, Nahid Khalil Elfaki, Rasha Elsayed Ahmed, Hala Ahmed Elsayes, Yahya Hussein Ahmed Abdalla, Osman Babiker Osmsn Mohammed, Absar Ahmed Qureshi, Friyal Alqahtani, Sirajudeen Shaik Alavudeen (2023) **Parental health-seeking behavior on self-medication, antibiotic use, and antimicrobial resistance in children.**. *Saudi pharmaceutical journal : SPJ : the official publication of the Saudi Pharmaceutical Society* 31(9), p. 101712, ISSN 1319-0164, doi:10.1016/j.jsps.2023.101712

Grace Mambula, Deborah Nanjebe, Aurelia Munene, Ousmane Guindo, Aichatou Salifou, Abdoul-Aziz Mamaty, Susan Rattigan, Sally Ellis, Nathalie Khavessian, Rob W van der Pluijm, Caroline Marquer, Irene Aicha Adehossi, Céline Langendorf (2023) **Practices and challenges related to antibiotic use in paediatric treatment in hospitals and health centres in Niger and Uganda: a mixed methods study.**. *Antimicrobial resistance and infection control* 12(1), p. 67, ISSN 2047-2994, doi:10.1186/s13756-023-01271-7

Bingqing Bi, Jiangmei Qin, Lifang Zhang, Chunmei Lin, Shugang Li, Yanchun Zhang (2023) **Systematic Review and Meta-Analysis of Factors Influencing Self-Medication in Children.**. *Inquiry : a journal of medical care organization, provision and financing* 60, p. 2147483647, ISSN 1945-7243, doi:10.1177/00469580231159744

Bo Yan, Zhenke He, Shixin Dong, Hailati Akezhuoli, Xin Xu, Xiaomin Wang, Xudong Zhou (2023) **The moderating effect of parental skills for antibiotic identification on the link between parental skills for antibiotic use and inappropriate antibiotic use for children in China.**. *BMC public health* 23(1), p. 156, ISSN 1471-2458, doi:10.1186/s12889-023-15099-8

Javad Nazari, Nahid Chezani-Sharahi, Babak Eshrati, Ali Yadegari, Mobin Naghshbandi, Hamidreza Movahedi, Rahmatollah Moradzadeh (2022) **Prevalence and determinants of self-medication consumption of antibiotics in children in Iran: A population-based cross-sectional study, 2018-19.**. *PloS one* 17(12), ISSN 1932-6203, doi:10.1371/journal.pone.0278843

Fabrizio Bert, Christian Previti, Francesco Calabrese, Giacomo Scaioli, Roberta Siliquini (2022) **Antibiotics Self Medication among Children: A Systematic Review.**. *Antibiotics (Basel, Switzerland)* 11(11), ISSN 2079-6382, doi:10.3390/antibiotics11111583

Nan Christine Wang (2022) **Pre-Visit Use of Non-Prescribed Antibiotics among Child Patients in China: Prevalence, Predictors, and Association with Physicians' Prescribing of Antibiotics at Medical Visits.**. *Antibiotics (Basel, Switzerland)* 11(11), ISSN 2079-6382, doi:10.3390/antibiotics11111553

Jhon Camacho Cruz, Carolina Zambrano Perez, Maria Carolina Sánchez Cabrera, Estefania Robledo Lopez, Pablo Vásquez Hoyos, Diana Rojas Rojas, Andrea Ortiz Montaña (2022) **Factors associated with self-medication of antibiotics by caregivers in pediatric patients attending the emergency department: a case-control study.**. *BMC pediatrics* 22(1), p. 520, ISSN 1471-2431, doi:10.1186/s12887-022-03572-z

Redson Biswick Machongo, Alinane Linda Nyondo Mipando (2022) **"I don't hesitate to use the left-over antibiotics for my child" practices and experiences with antibiotic use among caregivers of paediatric patients at Zomba central hospital in Malawi.**. *BMC pediatrics* 22(1), p. 466, ISSN 1471-2431, doi:10.1186/s12887-022-03528-3

Lilian Nkinda, Manase Kilonzi, Fatuma F Felix, Ritah Mutagonda, David T Myemba, Dorkasi L Mwakawanga, Upendo Kibwana, Belinda J Njiro, Harrieth P Ndumwa, Rogers Mwakalukwa, Gerald Makuka, Samson W Kubigwa, Alphonce I Marealle, Wigilya P Mikomangwa, Godfrey Sambayi, Peter P Kunambi, Betty A Maganda, Nathanael Sirili, Rashid Mfaume, Arapha Bashir Nshau, George M Bwire, Robert Scherpbier, Elevanie Nyankesha, Pacifique Ndayishimiye (2022) **Drivers of irrational use of antibiotics among children: a mixed-method study among prescribers and dispensers in Tanzania.**. *BMC health services research* 22(1), p. 961, ISSN 1472-6963, doi:10.1186/s12913-022-08359-7

Richard Nyeko, Felix Otim, Evelyn Miriam Obiya, Catherine Abala (2022) **Pre-hospital exposures to antibiotics among children presenting with fever in northern Uganda: a facility-based cross-sectional study.**. *BMC pediatrics* 22(1), p. 322, ISSN 1471-2431, doi:10.1186/s12887-022-03375-2

Nandini Sharma, Saurav Basu, Subhanwita Manna, Pragya Sharma, Shivani Rao, Kushagr Duggal, Harpreet Kaur, Pawan Kumar, Shikha T Malik (2022) **Health-Seeking Behaviour for Childhood Ailments in Caregivers of Under-Five Children in an Urban Resettlement Colony in Delhi, India.**. *Cureus* 14(4), ISSN 2168-8184, doi:10.7759/cureus.24404

Gillian A Levine, Julia Bielicki, Günther Fink (2022) **Cumulative Antibiotic Exposure in the First 5 Years of Life: Estimates for 45 Low- and Middle-Income Countries From Demographic and Health Survey Data.**. *Clinical infectious diseases : an official publication of the Infectious Diseases Society of America* 75(9), pp. 1537-1547, ISSN 1537-6591, doi:10.1093/cid/ciac225

Jing Yuan, Wandi Du, Zhiping Li, Qiao Deng, Guo Ma (2021) **Prevalence and Risk Factors of Self-Medication Among the Pediatric Population in China: A National Survey.**. *Frontiers in public health* 9, p. 770709, ISSN 2296-2565, doi:10.3389/fpubh.2021.770709

Asma Ben Mabrouk, Fatma Larbi Ammari, Amina Werdani, Nesrine Jemmali, Jihene Chelli, Houcem Elomma Mrabet, Ahmed Rassas, Mohamed Habib Sfar, Sana El Mhamdi, Bahri Mahjoub (2022) **Parental self-medication with antibiotics in a Tunisian pediatric center.**. *Therapie* 77(4), pp. 477-485, ISSN 1958-5578, doi:10.1016/j.therap.2021.10.007

Aisha F Badr, Raneyah A Humedi, Nada A Alfarsi, Haifa A Alghamdi (2021) **Rapid antigen detection test (RADT) for pharyngitis diagnosis in children: Public and pharmacist perception.**. *Saudi pharmaceutical journal : SPJ : the official publication of the Saudi Pharmaceutical Society* 29(7), pp. 677-681, ISSN 1319-0164, doi:10.1016/j.jsps.2021.04.029

Naseem Amin Dhedhi, Hiba Ashraf, Naila Baig Ansari, Sundus Iftikhar (2021) **Self-medication among people visiting outpatient clinics of a Tertiary care hospital, Karachi.**. *Journal of family medicine and primary care* 10(2), pp. 773-779, ISSN 2249-4863, doi:10.4103/jfmpc.jfmpc_1887_20

Maram Khazen, Nurit Guttman (2021) **'Nesef Doctora'-When mothers are considered to be 'half-doctors': Self-medication with antibiotics and gender roles in the Arab society in Israel.**. *Sociology of health & illness* 43(2), pp. 408-423, ISSN 1467-9566, doi:10.1111/1467-9566.13229

Leesa Lin, Stephan Harbarth, James R Hargreaves, Xudong Zhou, Leah Li (2021) **Large-scale survey of parental antibiotic use for paediatric upper respiratory tract infections in China: implications for stewardship programmes and national policy.**. *International journal of antimicrobial agents* 57(4), p. 106302, ISSN 1872-7913, doi:10.1016/j.ijantimicag.2021.106302

Jianxiong Wu, Fengjie Yang, Heping Yang, Guopeng Zhang, Ketao Mu, Jie Feng, Jing Wang, Xiaoxv Yin (2021) **Prevalence of antibiotic self-medication behavior and related factors among children aged 0 to 5 years.**. *Expert review of anti-infective therapy* 19(9), pp. 1157-1164, ISSN 1744-8336, doi:10.1080/14787210.2021.1882303

Janet Sultana, Gianluca Trifirò, Valentina Ientile, Andrea Fontana, Francesco Rossi, Annalisa Capuano, Carmen Ferrajolo (2020) **Traceability of Pediatric Antibiotic Purchasing Pathways in Italy: A Nationwide Real-World Drug Utilization Analysis.**. *Frontiers in pharmacology* 11, p. 1232, ISSN 1663-9812, doi:10.3389/fphar.2020.01232

Jiayao Xu, Xiaomin Wang, Kai Sing Sun, Leesa Lin, Xudong Zhou (2020) **Parental self-medication with antibiotics for children promotes antibiotic over-prescribing in clinical settings in China.**. *Antimicrobial resistance and infection control* 9(1), p. 150, ISSN 2047-2994, doi:10.1186/s13756-020-00811-9

Vera A Appiah, George A Pesewu, Fleischer C N Kotey, Alahaman Nana Boakye, Samuel Duodu, Edem M A Tette, Mame Y Nyarko, Eric S Donkor (2020) **Staphylococcus aureus Nasal Colonization among Children with Sickle Cell Disease at the Children's Hospital, Accra: Prevalence, Risk Factors, and Antibiotic Resistance.**. *Pathogens (Basel, Switzerland)* 9(5), ISSN 2076-0817, doi:10.3390/pathogens9050329

Vincenzo De Sanctis, Ashraf T Soliman, Shahina Daar, Salvatore Di Maio, Rania Elalaily, Bernadette Fiscina, Christos Kattamis (2020) **Prevalence, attitude and practice of self-medication among adolescents and the paradigm of dysmenorrhea self-care management in different countries.**. *Acta bio-medica : Atenei Parmensis* 91(1), pp. 182-192, ISSN 2531-6745, doi:10.23750/abm.v91i1.9242

Yannan Xu, Jingjing Lu, Chenhui Sun, Xiaomin Wang, Yanhong Jessika Hu, Xudong Zhou (2020) **A cross-sectional study of antibiotic misuse among Chinese children in developed and less developed provinces.**. *Journal of infection in developing countries* 14(2), pp. 129-137, ISSN 1972-2680, doi:10.3855/jidc.11938

Monika Kamati, Brian Godman, Dan Kibuule (2019) **Prevalence of Self-Medication for Acute Respiratory Infections in Young Children in Namibia: Findings and Implications.**. *Journal of research in pharmacy practice* 8(4), pp. 220-224, ISSN 2319-9644, doi:10.4103/jrpp.JRPP_19_121

Chenhui Sun, Yanhong Jessika Hu, Xiaomin Wang, Jingjing Lu, Leesa Lin, Xudong Zhou (2019) **Influence of leftover antibiotics on self-medication with antibiotics for children: a cross-sectional study from three Chinese provinces.**. *BMJ open* 9(12), ISSN 2044-6055, doi:10.1136/bmjopen-2019-033679

Maja Farkaš, Daniela Glažar Ivče, Senka Stojanović, Martina Mavrinac, Vladimir Mićović, Arjana Tambić Andrašević (2019) **Parental Knowledge and Awareness Linked to Antibiotic Use and Resistance: Comparison of Urban and Rural Population in Croatia.**. *Microbial drug resistance (Larchmont, N.Y.)* 25(10), pp. 1430-1436, ISSN 1931-8448, doi:10.1089/mdr.2018.0424

Grace-Ange Elong Ekambi, Cécile Okalla Ebongue, Ida Calixte Penda, Emmanuel Nnanga Nga, Emmanuel Mpondo Mpondo, Carole Else Eboumbou Moukoko (2019) **Knowledge, practices and attitudes on antibiotics use in Cameroon: Self-medication and prescription survey among children, adolescents and adults in private pharmacies.**. *PloS one* 14(2), ISSN 1932-6203, doi:10.1371/journal.pone.0212875

Jing Cheng, Jing Chai, Yehuan Sun, Debin Wang (2019) **Antibiotics use for upper respiratory tract infections among children in rural Anhui: children's presentations, caregivers' management, and implications for public health policy.**. *Journal of public health policy* 40(2), pp. 236-252, ISSN 1745-655X, doi:10.1057/s41271-019-00161-w

Calixte Ida Penda, Else Carole Eboumbou Moukoko, Julien Franck Ngomba Youmba, Emmanuel Mpondo Mpondo (2018) **Characterization of pharmaceutical medication without a medical prescription in children before hospitalization in a resource-limited setting, Cameroon.**. *The Pan African medical journal* 30, p. 302, ISSN 1937-8688, doi:10.11604/pamj.2018.30.302.16321

Ifeoma M Ezeonu, Ntun W Ntun, Kenneth O Ugwu (2017) **Intestinal candidiasis and antibiotic usage in children: case study of Nsukka, South Eastern Nigeria.**. *African health sciences* 17(4), pp. 1178-1184, ISSN 1729-0503, doi:10.4314/ahs.v17i4.27

Verica Ivanovska, Bistra Angelovska, Liset van Dijk, Milka Zdravkovska, Hubert G Leufkens, Aukje K Mantel-Teeuwisse (2018) **Change in parental knowledge, attitudes and practice of antibiotic use after a national intervention programme.**. *European journal of public health* 28(4), pp. 724-729, ISSN 1464-360X, doi:10.1093/eurpub/ckx240

Jie Chang, Bing Lv, Shan Zhu, Jiale Yu, Yu Zhang, Dan Ye, Muhammad Majid Aziz, Caijun Yang, Yu Fang (2018) **Non-prescription use of antibiotics among children in urban China: a cross-sectional survey of knowledge, attitudes, and practices.**. *Expert review of anti-infective therapy* 16(2), pp. 163-172, ISSN 1744-8336, doi:10.1080/14787210.2018.1425616

Raghda M El-Hawy, Mohamed I Ashmawy, Menna M Kamal, Hager A Khamis, Naglaa M Abo El-Hamed, Gehad I Eladely, Mayar H Abdo, Yosra Hashem, Marwa Ramadan, Dalia A Hamdy (2017) **Studying the knowledge, attitude and practice of antibiotic misuse among Alexandria population.**. *European journal of hospital pharmacy : science and practice* 24(6), pp. 349-354, ISSN 2047-9964, doi:10.1136/ejhpharm-2016-001032

Helena C Maltezou, Xanthi Dedoukou, Hara Asimaki, Ioanna Kontou, Loukia Ioannidou, Konstantina Mitromara, Kalliopi Theodoridou, Panos Katerelos, Maria Theodoridou (2017) **Consumption of antibiotics by children in Greece: a cross-sectional study.**. *International journal of pediatrics & adolescent medicine* 4(3), pp. 108-111, ISSN 2352-6467, doi:10.1016/j.ijpam.2017.04.002

Béatrice Demoré, Lucie Mangin, Gianpiero Tebano, Céline Pulcini, Nathalie Thilly (2017) **Public knowledge and behaviours concerning antibiotic use and resistance in France: a cross-sectional survey.**. *Infection* 45(4), pp. 513-520, ISSN 1439-0973, doi:10.1007/s15010-017-1015-2

Ruili Li, Feng Xiao, Xiaoguo Zheng, Huimin Yang, Lihong Wang, Delu Yin, Tao Yin, Qianqian Xin, Bowen Chen (2016) **Antibiotic misuse among children with diarrhea in China: results from a national survey.**. *PeerJ* 4, ISSN 2167-8359, doi:10.7717/peerj.2668

Anita Kotwani, Chand Wattal, P C Joshi, Kathleen Holloway (2016) **Knowledge and perceptions on antibiotic use and resistance among high school students and teachers in New Delhi, India: A qualitative study.**. *Indian journal of pharmacology* 48(4), pp. 365-371, ISSN 1998-3751, doi:10.4103/0253-7613.186208

K A Akinlade, J O Akinyemi, O I Fawole (2015) **Knowledge of hazards of antibiotics self-medication by mothers for under-fives in rural community of South-west Nigeria.**. *African journal of medicine and medical sciences* 44(4), pp. 303-309, ISSN 0309-3913

Caroline Saint-Martin, Lukshe Kanagaratnam, Paul de Boissieu, Brahim Azzouz, Malak Abou Taam, Thierry Trenque (2016) **[Adverse drug reactions in pediatrics: Experience of a regional pharmacovigilance center].**. *Therapie* 71(5), pp. 467-473, ISSN 0040-5957, doi:10.1016/j.therap.2016.04.001

Salvatore Italia, Helmut Brand, Joachim Heinrich, Dietrich Berdel, Andrea von Berg, Silke Britta Wolfenstetter (2015) **Utilization of self-medication and prescription drugs among 15-year-old children from the German GINIplus birth cohort.**. *Pharmacoepidemiology and drug safety* 24(11), pp. 1133-1143, ISSN 1099-1557, doi:10.1002/pds.3829

Eglė Pavydė, Vincentas Veikutis, Asta Mačiulienė, Vytautas Mačiulis, Kęstutis Petrikonis, Edgaras Stankevičius (2015) **Public Knowledge, Beliefs and Behavior on Antibiotic Use and Self-Medication in Lithuania.**. *International journal of environmental research and public health* 12(6), pp. 7002-7016, ISSN 1660-4601, doi:10.3390/ijerph120607002

Milica Paut Kusturica, Zdenko Tomic, Zoran Bukumiric, Ljiljana Ninkovic, Ana Tomas, Nebojsa Stilinovic, Ana Sabo (2015) **Home pharmacies in Serbia: an insight into self-medication practice.**. *International journal of clinical pharmacy* 37(2), pp. 373-378, ISSN 2210-7711, doi:10.1007/s11096-015-0071-x

Emel Peker, Erkan M Sahin, Naci Topaloğlu, Ayşegül Uludağ, Hasre Ağaoğlu, Selen Güngör (2016) **Knowledge, attitude and behavior of mothers related to acute respiratory infections.**. *Minerva pediatrica* 68(2), pp. 114-120, ISSN 1827-1715

Nathalie Eckel, Giselle Sarganas, Ingrid-Katharina Wolf, Hildtraud Knopf (2014) **Pharmacoepidemiology of common colds and upper respiratory tract infections in children and adolescents in Germany.**. *BMC pharmacology & toxicology* 15, p. 44, ISSN 2050-6511, doi:10.1186/2050-6511-15-44

Maria J B Cruz, Lays F N Dourado, Emerson C Bodevan, Renata A Andrade, Delba F Santos (2014) **Medication use among children 0-14 years old: population baseline study.**. *Jornal de pediatria* 90(6), pp. 608-615, ISSN 1678-4782, doi:10.1016/j.jped.2014.03.004

Miao Yu, Genming Zhao, Cecilia Stålsby Lundborg, Yipin Zhu, Qi Zhao, Biao Xu (2014) **Knowledge, attitudes, and practices of parents in rural China on the use of antibiotics in children: a cross-sectional study.**. *BMC infectious diseases* 14, p. 112, ISSN 1471-2334, doi:10.1186/1471-2334-14-112

Verica Ivanovska, Milka Zdravkovska, Golubinka Bosevska, Bistra Angelovska (2013) **Antibiotics for upper respiratory infections: public knowledge, beliefs and self-medication in the Republic of Macedonia.**. *Prilozi (Makedonska akademija na naukite i umetnostite. Oddelenie za medicinski nauki)* 34(2), pp. 59-70, ISSN 1857-9345

Abdulaziz H Abobotain, Haytham A Sheerah, Faiza N Alotaibi, Abdulaziz U Joury, Rowayda M Mishiddi, Amna R Siddiqui, Abdulaziz Bin Saeed (2013) **Socio-demographic determinants of antibiotic misuse in children. A survey from the central region of Saudi Arabia.**. *Saudi medical journal* 34(8), pp. 832-840, ISSN 1658-3175

Lucie Ecker, Theresa J Ochoa, Martha Vargas, Luis J Del Valle, Joaquim Ruiz (2013) **Factors affecting caregivers' use of antibiotics available without a prescription in Peru.**. *Pediatrics* 131(6), ISSN 1098-4275, doi:10.1542/peds.2012-1970

Uchenna Ekwochi, Josephat M Chinawa, Ikechukwu Obi, Herbert A Obu, Samuel Agwu (2013) **Use and/or misuse of antibiotics in management of diarrhea among children in Enugu, Southeast Nigeria.**. *Journal of tropical pediatrics* 59(4), pp. 314-316, ISSN 1465-3664, doi:10.1093/tropej/fmt016

Rudi Emerson de Lima Procópio, Ingrid Reis da Silva, Mayra Kassawara Martins, João Lúcio de Azevedo, Janete Magali de Araújo (2012) **Antibiotics produced by Streptomyces.**. *The Brazilian journal of infectious diseases : an official publication of the Brazilian Society of Infectious Diseases* 16(5), pp. 466-471, ISSN 1678-4391, doi:10.1016/j.bjid.2012.08.014

Herbert A Obu, Josephat M Chinawa, Agozie C Ubesie, Christopher B Eke, Ikenna K Ndu (2012) **Paracetamol use (and/or misuse) in children in Enugu, South-East, Nigeria.**. *BMC pediatrics* 12, p. 103, ISSN 1471-2431, doi:10.1186/1471-2431-12-103

Aris Widayati, Sri Suryawati, Charlotte de Crespigny, Janet E Hiller (2015) **Beliefs about the use of nonprescribed antibiotics among people in Yogyakarta City, Indonesia: a qualitative study based on the theory of planned behavior.**. *Asia-Pacific journal of public health* 27(2), ISSN 1941-2479, doi:10.1177/1010539512445052

Josta de Jong, Jens H J Bos, Tjalling W de Vries, Lolkje T W de Jong-van den Berg (2012) **Antibiotic use in children and the use of medicines by parents.**. *Archives of disease in childhood* 97(6), pp. 578-581, ISSN 1468-2044, doi:10.1136/archdischild-2011-301150

Thi Hoan Le, Ellinor Ottosson, Thi Kim Chuc Nguyen, Bao Giang Kim, Peter Allebeck (2011) **Drug use and self-medication among children with respiratory illness or diarrhea in a rural district in Vietnam: a qualitative study.**. *Journal of multidisciplinary healthcare* 4, pp. 329-336, ISSN 1178-2390, doi:10.2147/JMDH.S22769

Kazeem Adeola Oshikoya, Henry Chukwura, Olisamedua Fidelis Njokanma, Idowu Odunayo Senbanjo, Iyabo Ojo (2011) **Incidence and cost estimate of treating pediatric adverse drug reactions in Lagos, Nigeria.**. *Sao Paulo medical journal = Revista paulista de medicina* 129(3), pp. 153-164, ISSN 1806-9460, doi:10.1590/s1516-31802011000300006

K D Mwambete, R Andrew (2010) **Knowledge on management of fever among mothers of undertens in Dar es Salaam, Tanzania.**. *East African journal of public health* 7(2), pp. 177-181, ISSN 0856-8960, doi:10.4314/eajph.v7i2.64721

Ganchimeg Togoobaatar, Nayu Ikeda, Moazzam Ali, Munkhbayarlakh Sonomjamts, Sarangerel Dashdemberel, Rintaro Mori, Kenji Shibuya (2010) **Survey of non-prescribed use of antibiotics for children in an urban community in Mongolia.**. *Bulletin of the World Health Organization* 88(12), pp. 930-936, ISSN 1564-0604, doi:10.2471/BLT.10.079004

Timothy F Landers, Yu-Hui Ferng, Jennifer Wong McLoughlin, Angela E Barrett, Elaine Larson (2010) **Antibiotic identification, use, and self-medication for respiratory illnesses among urban Latinos.**. *Journal of the American Academy of Nurse Practitioners* 22(9), pp. 488-495, ISSN 1745-7599, doi:10.1111/j.1745-7599.2010.00539.x

G Akello-Ayebare, J M Richters, A M Polderman, L G Visser (2010) **Healthcare-seeking strategies among displaced children in war-ridden northern Uganda: the case of malaria.**. *Annals of tropical medicine and parasitology* 104(5), pp. 369-376, ISSN 1364-8594, doi:10.1179/136485910X12743554760342

Grace Pfaffenbach, Francis S V Tourinho, Fabio Bucaretchi (2010) **Self-medication among children and adolescents.**. *Current drug safety* 5(4), pp. 324-328, ISSN 2212-3911, doi:10.2174/157488610792246028

Mukandu Basua Babintu Leyka, Prof Mylène Baum, Maiga Diadié, Mbela Kiyombo, Bavon Mupenda (2009) **[Education for self-administered antibiotic therapy: a pragmatic and ethical alternative for the treatment of STDs for the street youth of Kinshasa in the Democratic Republic of the Congo (RDC)].**. *Sante (Montrouge, France)* 19(4), pp. 217-225, ISSN 1157-5999, doi:10.1684/san.2009.0149

Mabrook Mohanna (2010) **Self-medication with Antibiotic in Children in Sana'a City, Yemen.**. *Oman medical journal* 25(1), pp. 41-43, ISSN 2070-5204, doi:10.5001/omj.2010.10

Francis S V Tourinho, Fábio Bucaretchi, Celso Stephan, Ricardo Cordeiro (2008) **Home medicine chests and their relationship with self-medication in children and adolescents.**. *Jornal de pediatria* 84(5), pp. 416-422, ISSN 1678-4782, doi:10.2223/JPED.1831

Arch G Mainous, Vanessa A Diaz, Mark Carnemolla (2008) **Factors affecting Latino adults' use of antibiotics for self-medication.**. *Journal of the American Board of Family Medicine : JABFM* 21(2), pp. 128-134, ISSN 1557-2625, doi:10.3122/jabfm.2008.02.070149

S E D Nsimba (2007) **Assessing the performance, practices and roles of drug sellers/dispensers and mothers'/guardians' behaviour for common childhood conditions in Kibaha district, Tanzania.**. *Tropical doctor* 37(4), pp. 197-201, ISSN 0049-4755, doi:10.1258/004947507782333099

Francis S V T Pereira, Fábio Bucaretchi, Celso Stephan, Ricardo Cordeiro (2007) **Self-medication in children and adolescents.**. *Jornal de pediatria* 83(5), pp. 453-458, ISSN 0021-7557, doi:10.2223/JPED.1703

Nguyen Quynh Hoa, Ann Ohman, Cecilia Stålsby Lundborg, Nguyen Thi Kim Chuc (2007) **Drug use and health-seeking behavior for childhood illness in Vietnam--a qualitative study.**. *Health policy (Amsterdam, Netherlands)* 82(3), pp. 320-329, ISSN 0168-8510, doi:10.1016/j.healthpol.2006.10.005

Hoang L Phuong, Peter J de Vries, Nico Nagelkerke, Phan T Giao, Le Q Hung, Tran Q Binh, Tran T Thanh Nga, Nguyen V Nam, Piet A Kager (2006) **Acute undifferentiated fever in Binh Thuan province, Vietnam: imprecise clinical diagnosis and irrational pharmaco-therapy.**. *Tropical medicine & international health : TM & IH* 11(6), pp. 869-879, ISSN 1360-2276, doi:10.1111/j.1365-3156.2006.01636.x

Elaine L Larson, Joann Dilone, Magaly Garcia, Janice Smolowitz (2006) **Factors which influence Latino community members to self-prescribe antibiotics.**. *Nursing research* 55(2), pp. 94-102, ISSN 0029-6562, doi:10.1097/00006199-200603000-00004

C C Ezechukwu, I Egbuonu, J O Chukwuka (2005) **Drug treatment of common childhood symptoms in Nnewi: what mothers do?**. *Nigerian journal of clinical practice* 8(1), pp. 1-3, ISSN 1119-3077

Nuzhat Yasmeen, Mohammad Riaz Khan (2005) **Spectrum of common childhood skin diseases: a single centre experience.**. *JPMA. The Journal of the Pakistan Medical Association* 55(2), pp. 60-63, ISSN 0030-9982

Neeta Parimi, Lexley M Pinto Pereira, P Prabhakar (2004) **Caregivers' practices, knowledge and beliefs of antibiotics in paediatric upper respiratory tract infections in Trinidad and Tobago: a cross-sectional study.**. *BMC family practice* 5, p. 28, ISSN 1471-2296, doi:10.1186/1471-2296-5-28

M Debré, M Clairicia, F Bonnaud, O Jubin, D Thiébaux-Boucard (2004) **[Feasibility of administering Tegeline at home. Retrospective study of efficacy, safety and tolerance].**. *Presse medicale (Paris, France : 1983)* 33(10), pp. 682-688, ISSN 0755-4982, doi:10.1016/s0755-4982(04)98714-3

Norbert Anyama, R O Adome (2003) **Community pharmaceutical care: an 8-month critical review of two pharmacies in Kampala.**. *African health sciences* 3(2), pp. 87-93, ISSN 1729-0503

Michael A Borg, Elizabeth Anne Scicluna (2002) **Over-the-counter acquisition of antibiotics in the Maltese general population.**. *International journal of antimicrobial agents* 20(4), pp. 253-257, ISSN 0924-8579, doi:10.1016/s0924-8579(02)00194-2

Daniel J Edwards, Peter B Richman, Keith Bradley, Barnet Eskin, Mark Mandell (2002) **Parental use and misuse of antibiotics: are there differences in urban vs. suburban settings?**. *Academic emergency medicine : official journal of the Society for Academic Emergency Medicine* 9(1), pp. 22-26, ISSN 1069-6563, doi:10.1111/j.1553-2712.2002.tb01162.x

A Henry, C Crowther (2000) **Patterns of medication use during and prior to pregnancy: the MAP study.**. *The Australian & New Zealand journal of obstetrics & gynaecology* 40(2), pp. 165-172, ISSN 0004-8666, doi:10.1111/j.1479-828x.2000.tb01140.x

J Halfvarsson, N Heijne, P Ljungman, M N Ham, G Holmgren, G Tomson (2000) **Knowing when but not how!--mothers' perceptions and use of antibiotics in a rural area of Viet Nam.**. *Tropical doctor* 30(1), pp. 6-10, ISSN 0049-4755, doi:10.1177/004947550003000105

P Bi, S Tong, K A Parton (2000) **Family self-medication and antibiotics abuse for children and juveniles in a Chinese city.**. *Social science & medicine (1982)* 50(10), pp. 1445-1450, ISSN 0277-9536, doi:10.1016/s0277-9536(99)00304-4

L Sombrero, M E Sunico, B Quiambao, M Lucero, S Gatchalian, M Leinonen, P Ruutu (1999) **Reliability of parental history of antibiotic use for Filipino children admitted with acute lower respiratory tract infection.**. *The American journal of tropical medicine and hygiene* 60(3), pp. 397-399, ISSN 0002-9637, doi:10.4269/ajtmh.1999.60.397

K Khalil, S R Khan, K Mazhar, B Kaijser, G B Lindblom (1998) **Occurrence and susceptibility to antibiotics of Shigella species in stools of hospitalized children with bloody diarrhea in Pakistan.**. *The American journal of tropical medicine and hygiene* 58(6), pp. 800-803, ISSN 0002-9637, doi:10.4269/ajtmh.1998.58.800

O Brändli, T Luterbacher, N Egli (1997) **[When and why are antibiotics indicated in airway infections (except pneumonia)?].**. *Praxis* 86(18), pp. 737-740, ISSN 1661-8157

(1997) **Multicenter study on self-medication and self-prescription in six Latin American countries. Drug Utilization Research Group, Latin America.**. *Clinical pharmacology and therapeutics* 61(4), pp. 488-493, ISSN 0009-9236, doi:10.1016/S0009-9236(97)90199-5

P A Oriol Torón, S Lou Arnal, M J Blasco Pérez-Aramendía, A I Sediles Cabello, I Pérez Ramírez (1994) **[Self health care when faced with acute pathology in childhood].**. *Atencion primaria* 14(2), pp. 616-618, ISSN 0212-6567

G A Oni, D A Schumann, E A Oke (1991) **Diarrhoeal disease morbidity, risk factors and treatments in a low socioeconomic area of Ilorin, Kwara State, Nigeria.**. *Journal of diarrhoeal diseases research* 9(3), pp. 250-257, ISSN 0253-8768

K Osinusi, C O Oyejide (1990) **Child care practices with respect to acute respiratory tract infection in a poor, urban community in Nigeria.**. *Reviews of infectious diseases* 12, ISSN 0162-0886, doi:10.1093/clinids/12.supplement_8.s1039

M Catalano, M A Almiron, A M Romeo, E Caruso, P Murtagh, J Harisiadi (1990) **Comparison between parental report and results of microbiologic agar assay for presence of antibiotic in urine of Argentinian children with acute lower respiratory tract infection.**. *Reviews of infectious diseases* 12, ISSN 0162-0886, doi:10.1093/clinids/12.supplement_8.s998

S J Utsalo, V Onoyom-Ita, M Ifeanyi-Chukwu, J O Akpan (1990) **Home medication and microbiological profile in chronic otitis media in some Nigerian children.**. *The Central African journal of medicine* 36(11), pp. 278-283, ISSN 0008-9176

A M Basu (1990) **Cultural influences on health care use: two regional groups in India.**. *Studies in family planning* 21(5), pp. 275-286, ISSN 0039-3665

H Haak, A P Hardon (1988) **Indigenised pharmaceuticals in developing countries: widely used, widely neglected.**. *Lancet (London, England)* 2(8611), pp. 620-621, ISSN 0140-6736, doi:10.1016/s0140-6736(88)90652-6

A Bamisaiye, T O Johnson (1988) **Planning PHC for a community: a baseline survey provides essential data.**. *Tropical doctor* 18(1), pp. 36-37, ISSN 0049-4755, doi:10.1177/004947558801800114

J Whyte, E Greenan (1976) **Pattern and quality of recording pre-admission drug treatment in paediatric patients.**. *British medical journal* 1(6001), pp. 61-63, ISSN 0007-1447, doi:10.1136/bmj.1.6001.61

**non-prescription antibiotics children**

*Publish or Perish 8.2.3944.8118 (basic report)
WinPosix (x64) edition, running on WinPosix 10.0.22000 (x64)*

**Search terms**

**Keywords:** non-prescription antibiotics children
**Years:** all

**Data retrieval**

**Data source:** PubMed
**Search date:** 2024-07-01 16:53:48 +0300
**Cache date:** 2024-07-01 13:51:48 +0300
**Search result:** [0] No error

***Important:*** *This data source does not provide citation counts.*

**Metrics**

**Reference date:** 2024-07-01 13:51:48 +0300
**Publication years:** 1995-2024
**Citation years:** 29 (1995-2024)
**Papers:** 23
**Citations:** 0
**Citations/year:** 0.00 (acc1=0, acc2=0, acc5=0, acc10=0, acc20=0)
**Citations/paper:** 0.00
**Authors/paper:** 5.43/5.0/4 (mean/median/mode)
**Age-weighted citation rate:** 0.00 (sqrt=0.00), 0.00/author
**Hirsch h-index:** 0 (a=0.00, m=0.00, 0 cites=0.0% coverage)
**Egghe g-index:** 0 (g/h=0.00, 0 cites=0.0% coverage)
**PoP hI,norm:** 0
**PoP hI,annual:** 0.00
**Fassin hA-index:** 0

**Results**

Meklit Melaku Bezie, Zufan Alamrie Asmare, Hiwot Altaye Asebe, Afework Alemu Lombebo, Bezawit Melak Fentie, Angwach Abrham Asnake, Beminate Lemma Seifu (2024) **Factors associated with the use of antibiotics for children presenting with illnesses with fever and cough obtained from prescription and non-prescription sources: a cross-sectional study of data for 37 sub-Saharan African countries.**. *BMC public health* 24(1), p. 1089, ISSN 1471-2458, doi:10.1186/s12889-024-18490-1

S Obaro, F Hassan-Hanga, N Medugu, R Olaosebikan, G Olanipekun, B Jibir, S Gambo, Theresa Ajose, Carissa Duru, B Ebruke, H D Davies (2023) **Comparison of bacterial culture with BioFire® FilmArray® multiplex PCR screening of archived cerebrospinal fluid specimens from children with suspected bacterial meningitis in Nigeria.**. *BMC infectious diseases* 23(1), p. 641, ISSN 1471-2334, doi:10.1186/s12879-023-08645-7

Kiara Olmeda, Barbara W Trautner, Lindsey Laytner, Juanita Salinas, Stephanie Marton, Larissa Grigoryan (2023) **Prevalence and Predictors of Using Antibiotics without a Prescription in a Pediatric Population in the United States.**. *Antibiotics (Basel, Switzerland)* 12(3), ISSN 2079-6382, doi:10.3390/antibiotics12030491

Yao Zhu, Xuewen Tang, Rui Yan, Zhujun Shao, Yang Zhou, Xuan Deng, Shuying Luo, Hanqing He (2021) **Non-prescription antibiotic use for cough among Chinese children under 5 years of age: a community-based cross-sectional study.**. *BMJ open* 11(12), ISSN 2044-6055, doi:10.1136/bmjopen-2021-051372

Aslınur Albayrak, Nazmi Mutlu Karakaş, Bensu Karahalil (2021) **Evaluation of parental knowledge, attitudes and practices regarding antibiotic use in acute upper respiratory tract infections in children under 18 years of age: a cross-sectional study in Turkey.**. *BMC pediatrics* 21(1), p. 554, ISSN 1471-2431, doi:10.1186/s12887-021-03020-4

Asma Ben Mabrouk, Fatma Larbi Ammari, Amina Werdani, Nesrine Jemmali, Jihene Chelli, Houcem Elomma Mrabet, Ahmed Rassas, Mohamed Habib Sfar, Sana El Mhamdi, Bahri Mahjoub (2022) **Parental self-medication with antibiotics in a Tunisian pediatric center.**. *Therapie* 77(4), pp. 477-485, ISSN 1958-5578, doi:10.1016/j.therap.2021.10.007

Winfried V Kern, Karel Kostev (2021) **Prevalence of and Factors Associated with Antibiotic Prescriptions in Patients with Acute Lower and Upper Respiratory Tract Infections-A Case-Control Study.**. *Antibiotics (Basel, Switzerland)* 10(4), ISSN 2079-6382, doi:10.3390/antibiotics10040455

Leesa Lin, Stephan Harbarth, James R Hargreaves, Xudong Zhou, Leah Li (2021) **Large-scale survey of parental antibiotic use for paediatric upper respiratory tract infections in China: implications for stewardship programmes and national policy.**. *International journal of antimicrobial agents* 57(4), p. 106302, ISSN 1872-7913, doi:10.1016/j.ijantimicag.2021.106302

Nam Vinh Nguyen, Nga Thi Thuy Do, Chuc Thi Kim Nguyen, Toan Khanh Tran, Phuc Dang Ho, Hanh Hong Nguyen, Huong Thi Lan Vu, Heiman F L Wertheim, H Rogier van Doorn, Sonia Lewycka (2020) **Community-level consumption of antibiotics according to the AWaRe (Access, Watch, Reserve) classification in rural Vietnam.**. *JAC-antimicrobial resistance* 2(3), ISSN 2632-1823, doi:10.1093/jacamr/dlaa048

Ra'fat Ali, Abdullah Shadeed, Hasan Fitian, Sa'ed H Zyoud (2020) **The difficulties experienced during the preparation and administration of oral drugs by parents at home: a cross-sectional study from Palestine.**. *BMC pediatrics* 20(1), p. 198, ISSN 1471-2431, doi:10.1186/s12887-020-02105-w

Yannan Xu, Jingjing Lu, Chenhui Sun, Xiaomin Wang, Yanhong Jessika Hu, Xudong Zhou (2020) **A cross-sectional study of antibiotic misuse among Chinese children in developed and less developed provinces.**. *Journal of infection in developing countries* 14(2), pp. 129-137, ISSN 1972-2680, doi:10.3855/jidc.11938

Eyosait Mekonnen Koji, Gebremedhin Beedemariam Gebretekle, Tinsae Alemayehu Tekle (2019) **Practice of over-the-counter dispensary of antibiotics for childhood illnesses in Addis Ababa, Ethiopia: a simulated patient encounter study.**. *Antimicrobial resistance and infection control* 8, p. 119, ISSN 2047-2994, doi:10.1186/s13756-019-0571-x

Calixte Ida Penda, Else Carole Eboumbou Moukoko, Julien Franck Ngomba Youmba, Emmanuel Mpondo Mpondo (2018) **Characterization of pharmaceutical medication without a medical prescription in children before hospitalization in a resource-limited setting, Cameroon.**. *The Pan African medical journal* 30, p. 302, ISSN 1937-8688, doi:10.11604/pamj.2018.30.302.16321

Fadare Joseph Olusesan, Olatunya Oladele Simeon, Ogundare Ezra Olatunde, Oluwayemi Isaac Oludare, Agaja Oyinkansola Tolulope (2017) **Prescription audit in a paediatric sickle cell clinic in South-West Nigeria: A cross-sectional retrospective study.**. *Malawi medical journal : the journal of Medical Association of Malawi* 29(4), pp. 285-289, ISSN 1995-7270, doi:10.4314/mmj.v29i4.1

Jie Chang, Bing Lv, Shan Zhu, Jiale Yu, Yu Zhang, Dan Ye, Muhammad Majid Aziz, Caijun Yang, Yu Fang (2018) **Non-prescription use of antibiotics among children in urban China: a cross-sectional survey of knowledge, attitudes, and practices.**. *Expert review of anti-infective therapy* 16(2), pp. 163-172, ISSN 1744-8336, doi:10.1080/14787210.2018.1425616

Handan Boztepe, Handan Özdemir, Çiğdem Karababa, Özlem Yıldız (2016) **Administration of oral medication by parents at home.**. *Journal of clinical nursing* 25(21), pp. 3345-3353, ISSN 1365-2702, doi:10.1111/jocn.13460

Emel Peker, Erkan M Sahin, Naci Topaloğlu, Ayşegül Uludağ, Hasre Ağaoğlu, Selen Güngör (2016) **Knowledge, attitude and behavior of mothers related to acute respiratory infections.**. *Minerva pediatrica* 68(2), pp. 114-120, ISSN 1827-1715

Verica Ivanovska, Milka Zdravkovska, Golubinka Bosevska, Bistra Angelovska (2013) **Antibiotics for upper respiratory infections: public knowledge, beliefs and self-medication in the Republic of Macedonia.**. *Prilozi (Makedonska akademija na naukite i umetnostite. Oddelenie za medicinski nauki)* 34(2), pp. 59-70, ISSN 1857-9345

Djanilson Barbosa Dos Santos, Mauricio Lima Barreto, Helena Lutescia Luna Coelho (2011) **Use of prescribed and non-prescribed medications among children living in poor areas in the city of Salvador, Bahia State, Brazil.**. *Cadernos de saude publica* 27(10), pp. 2032-2040, ISSN 1678-4464, doi:10.1590/s0102-311x2011001000016

Ganchimeg Togoobaatar, Nayu Ikeda, Moazzam Ali, Munkhbayarlakh Sonomjamts, Sarangerel Dashdemberel, Rintaro Mori, Kenji Shibuya (2010) **Survey of non-prescribed use of antibiotics for children in an urban community in Mongolia.**. *Bulletin of the World Health Organization* 88(12), pp. 930-936, ISSN 1564-0604, doi:10.2471/BLT.10.079004

D Price, M Thomas, G Mitchell, C Niziol, R Featherstone (2003) **Improvement of asthma control with a breath-actuated pressurised metred dose inhaler (BAI): a prescribing claims study of 5556 patients using a traditional pressurised metred dose inhaler (MDI) or a breath-actuated device.**. *Respiratory medicine* 97(1), pp. 12-19, ISSN 0954-6111, doi:10.1053/rmed.2002.1426

P T Ngoc, J P Deschamps (1998) **[Global approach to diarrhea in children in Vietnam: the experience of Ho Chi Minh City no. 1 pediatric hospital].**. *Sante publique (Vandoeuvre-les-Nancy, France)* 10(1), pp. 87-98, ISSN 0995-3914

N Hawkins, J Golding (1995) **A survey of the administration of drugs to young infants. The Alspac Survey Team. Avon Longitudinal Study of Pregnancy and Childhood.**. *British journal of clinical pharmacology* 40(1), pp. 79-82, ISSN 0306-5251, doi:10.1111/j.1365-2125.1995.tb04539.x

Hinari(109)

((TitleCombined:(Non-prescription)) OR (TitleCombined:(without OR prescription)) OR (self-prescription) OR (self-medication) OR (over the counter)) AND ((Antibiotics) OR (Antibacterials) OR (Antiinfectives)) AND ((child) OR (Children) OR (pediatrics) OR (under five) OR (under eighteen) OR (school age)) AND (adolescents)

Google scholar=3

allintitle: non-prescription antibiotics children

Citation tracking=2 studies
